# Burden of chronic kidney disease and its attributable risk factors in 204 countries and territories, 1990–2021: Results from the Global Burden of Disease study 2021

**DOI:** 10.1097/MD.0000000000050111

**Published:** 2026-08-07

**Authors:** Wuyuan Tao, Chunlan Chen, Jinfeng Zhang, Xiangyue Jiang, Liuxia Deng

**Affiliations:** a Department of Emergency, Bao’an District People’s Hospital of Shenzhen, 118 Longjing 2nd Road, Shenzhen, Guangdong, China; b Department of Nephrology, Bao’an District People’s Hospital of Shenzhen, 118 Longjing 2nd Road, Shenzhen, Guangdong, China.

**Keywords:** chronic kidney disease, DALYs, Global Burden of Disease, mortality, prevalence

## Abstract

Chronic kidney disease (CKD) remains a major contributor to global mortality and morbidity. This study reports the global, regional, and national burden of CKD and its attributable risk factors from 1990 to 2021. Data were systematically analyzed using the Global Burden of Disease 2021 study. CKD prevalence, deaths, and disability-adjusted life years (DALYs), along with their attributable risk factors, were retrieved for 204 countries and territories from 1990 to 2021. Temporal trends were assessed using the percentage change in age‑standardized rates, with stratified analyses by Global Burden of Disease super‑region, country, age, sex, and sociodemographic index quintiles. In 2021, CKD affected 673.7 million people worldwide, resulting in 1.53 million deaths and causing 44.45 million DALYs. Key findings include: first, the global age-standardized prevalence rate of CKD was 8006 per 100,000 people, a 4.5% decrease since 1990. However, the age-standardized mortality rate increased by 24.6% to 18.5 per 100,000. Second, the highest CKD prevalence was observed in Central Asia, Southeast Asia, and South Asia, while the lowest was found in Western Europe and Eastern Sub-Saharan Africa. Third, high blood glucose, hypertension, and obesity were identified as the leading contributors to CKD-related DALYs globally. The global burden of CKD continues to rise, with mortality and DALYs increasing particularly in low- and middle-income countries. Urgent public health strategies targeting high blood glucose, hypertension, and obesity are critical to reducing the rising global burden of CKD.

## 1. Introduction

In 2017, the global prevalence of chronic kidney disease (CKD) was estimated to be 9.1%, affecting approximately 700 million people, making CKD one of the most significant global public health issues today^.[1]^ By 2040, CKD is projected to become the fifth leading cause of death worldwide, marking it as one of the most challenging noncommunicable diseases (NCDs) globally^.[2]^ The causes of CKD are multifaceted, with metabolic disorders such as diabetes, hypertension, and obesity being key contributors, closely linked to global aging populations and unhealthy lifestyles.^[3]^ In recent years, the burden of CKD has continued to rise globally, especially in low- and middle-income countries, where the high incidence and mortality rates of CKD pose significant challenges to healthcare systems and severely reduce the quality of life for patients.^[4]^

The increasing global burden of CKD is reflected not only in its rising prevalence but also in substantial increases in the age-standardized mortality rate (ASMR) and the age-standardized disability-adjusted life year (DALY) rate. As a frequent comorbidity of multiple chronic diseases, CKD often progresses rapidly from early to advanced stages, markedly reducing patient survival.^[5]^ Recent epidemiological data indicate that CKD has distinct distribution characteristics across different age groups and genders, closely related to socioeconomic factors, environmental conditions, and disparities in healthcare resource allocation.^[6]^ While the prevalence of CKD has declined in some high-income countries, the rate of increase in CKD burden in many low- and middle-income countries far exceeds the global average.^[7]^

To address the growing global health threat of CKD, the World Health Organization, along with national governments and healthcare institutions, has been actively promoting policy interventions, including early screening, disease management, and lifestyle improvements.^[8]^ However, despite these efforts, the global burden of CKD continues to rise, posing substantial challenges to healthcare systems, particularly in resource-limited regions. In light of this, accurately assessing global CKD trends and its attributable risk factors is crucial to providing policymakers with evidence-based guidance, ensuring the effective allocation of resources and implementing appropriate preventive and intervention measures to mitigate this burden.

This study uses data from the Global Burden of Disease (GBD) 2021 study to assess global and regional trends in CKD from 1990 to 2021 and to examine its association with key attributable risk factors. By analyzing age-standardized incidence, prevalence, mortality, and DALY rates, we aim to characterize CKD trajectories across different sociodemographic contexts, thereby providing evidence to guide global prevention and management strategies. Compared to previous GBD‑based CKD studies that used earlier data releases, our study incorporates the latest GBD 2021 data, which introduces a counterfactual risk analysis framework that addresses unexplained heterogeneity across different input datasets and supplements traditional relative risk estimates.^[9]^ This methodological improvement, together with an extended observation period, enhances the reliability of our attributable burden estimates.

## 2. Methods

### 2.1. Data sources and methodology

This study is a secondary analysis of CKD burden data from the GBD 2021 study, which estimated the burden of 371 diseases and injuries and 87 risk factors across 204 countries and territories within 21 regions from 1990 to 2021.^[10]^ The GBD 2021 study synthesizes heterogeneous primary data from diverse sources, including cross-sectional surveys, vital registration systems, and scientific literature. To ensure comparability and consistency of global estimates, the GBD 2021 study employed advanced modeling techniques, including spatiotemporal Gaussian process regression and DisMod-MR2.1, a Bayesian meta-regression tool that imputes missing values by leveraging covariate information and applying spatiotemporal smoothing.^[11]^

We extracted data on the incidence, prevalence, mortality, and DALYs for CKD (cause ID: 589) from the Global Health Data Exchange. These data were used to analyze the status and temporal trends of the CKD burden. This study used publicly available, de-identified data from the GBD 2021 study. As the analysis was based exclusively on aggregated population-level data, it was exempt from requirements for ethics approval and informed consent.

### 2.2. The definition of CKD

CKD is defined as a persistent estimated glomerular filtration rate of < 60 mL/ (min/1.73 m^2^), albuminuria (urine albumin-to-creatinine ratio ≥ 30 mg/g), or other markers of kidney damage for at least 3 months.^[12]^ Following systematic recalibration of serum creatinine values across all data sources using population-specific distributions for comparability, the estimated glomerular filtration rate was calculated according to age using the CKD-epidemiology collaboration (≥ 18 years) or Schwartz (< 18 years) equation (Table S1, Supplemental Digital Content 1).

Notably, most population-based studies incorporated in the GBD study are cross-sectional, providing only single-timepoint measurements of kidney function. To reconcile this with the clinical duration requirement, the GBD framework applies a microsimulation prediction model for chronicity adjustment. For each individual identified with abnormal kidney function, this model estimates a probability weight (ranging from 0–1) that the abnormality represents chronic, persistent kidney dysfunction. This weight is generated based on the severity of the index measurement and individual covariates such as age and comorbidities.^[13]^

In the GBD 2021 classification, CKD corresponds to the International Classification of Diseases, 10th Revision, with codes D63.1, E08.2-E08.29, E10.2-E10.29, E11.2-E11.29, E12.2, E13.2-E13.29, E14.2, I12-I13.9, N02-N08.8, N15.0, N17-N19, P96.0-P96.0, and to International Classification of Diseases, 9th Revision, with codes 249.4 to 249.41, 250.4 to 250.49, 285.21, 403 to 404.93, 581 to 587.91.^[10]^

### 2.3. Statistical analysis

To analyze the global and regional burden of CKD, we assessed temporal changes in prevalence, mortality, and DALYs from 1990 to 2021 at the global, regional, and national levels using descriptive statistics. Age-standardized rates were calculated to enable comparisons across regions and over time, and percentage changes between 1990 and 2021 were used to quantify long-term trends. Regional analyses covered 21 GBD regions, while national analyses examined the CKD burden in 204 countries.

To better characterize population-specific patterns of CKD burden, we performed stratified analyses by age and gender. Age-standardized rates were calculated to describe trends in prevalence, mortality, and DALYs across age groups and between sexes. We further identified age-specific peaks for each indicator and compared sex-specific differences within these age groups to reveal variations in CKD burden across demographic subpopulations.

To illustrate the link between disease burden and socioeconomic development, we analyzed the association between the sociodemographic index (SDI) and CKD burden. The SDI is a composite indicator incorporating per capita income, educational attainment, and fertility rate.^[14]^ Through SDI stratification, we revealed the variation in CKD burden across different SDI levels.

To investigate the relationship between CKD burden and key risk factors, we employed comparative risk assessment methods to estimate the contribution of specific risk factors. We evaluated all CKD‑related risk factors available in the GBD 2021 framework, including behavioral, metabolic, and environmental risk factors (Table S2, Supplemental Digital Content 2). Among these, 3 metabolic risk factors (high blood glucose, elevated systolic blood pressure, and high body mass index) were identified as the leading contributors to CKD‑related DALYs globally and are therefore the focus of this report. These 3 risk factors were selected based on established causal links to CKD, data availability, and their dominant contribution to the overall attributable burden. The comparative risk assessment framework was applied following GBD 2021 standard procedures: for each risk factor, we defined a theoretical minimum risk exposure level, estimated the population exposure distribution, calculated the population attributable fraction using the relative risk function and the counterfactual exposure distribution, and then multiplied population attributable fraction by total CKD‑related DALYs to obtain the attributable burden.^[9]^ The analysis results were stratified by global, regional, gender, and age categories.

All estimates from the GBD 2021 study are presented with 95% uncertainty intervals, defined as the 2.5th and 97.5th percentiles of 1000 draws from the posterior distribution of each estimate.^[10]^ These intervals represent the comprehensive uncertainty propagated through the complete modeling pipeline. Regions with sparse underlying data (particularly certain sub-Saharan African regions) are characterized by substantially wider uncertainty intervals and should therefore be interpreted with corresponding caution.

All analyses were performed using R software (version 4.2.2, Posit PBC, Boston, Massachusetts), and results were visualized with tools such as ggplot2 to ensure the rigor of the analytical process and the clarity of result presentation.

## 3. Results

### 3.1. Global level

In 2021, there were 673.7 million global cases of CKD, with an age‑standardized prevalence rate of 8006 per 100,000 people, and 44.45 million DALYs, with an age‑standardized rate of 529.6 per 100,000 people; both indicators showed a decreasing trend since 1990 (Table 1). However, CKD caused 1.53 million deaths in 2021, with an ASMR of 18.5 per 100,000, reflecting a 24.6% increase since 1990.

**Table 1:** Prevalent cases, deaths, and DALYs for CKD in 2021, and percentage change in ASRs per 100 000, by GBD region, from 1990 to 2021.

	Prevalence (95% UI)			Deaths (95% UI)			DALYs (95% UI)		
	No, in millions (95% UI)	ASRs per 100 000 (95% UI)	Percentage change in ASRs from 1990 to 2021	No. in thousands (95% UI)	ASRs per 100 000 (95% UI)	Percentage change in ASRs from 1990 to 2021	No. in thousands (95% UI)	ASRs per 100 000 (95% UI)	Percentage change in ASRs from 1990 to 2021
Global	673.7 (629.1–722.4)	8006 (7482.1–8575.6)	−4.5 (−6.2–−3)	1527.6 (1389.4–1638.9)	18.5 (16.7–19.9)	24.6 (2.4–43.3)	44,453.7 (40,840.8–48,508.5)	529.6 (486.2–577.4)	−22.1 (−28 –−14.7)
East Asia	122.8 (113.6–132.1)	6258.1 (5823.1–6729.8)	2.4 (1.6–3.2)	217.3 (178–259.1)	11.1 (9.2–13.2)	51.6 (37.5–66.7)	6486.2 (5538.1–7597.4)	322.4 (275.4–377.3)	−2.9 (−23.8–24.9)
Oceania	0.8 (0.7–0.8)	7950.7 (7408.8–8567.7)	−0.7 (−1.5–0.2)	1.5 (1.3–1.9)	21.6 (18–26.4)	20.8 (−23.7–50.5)	65.8 (55.6–77.8)	699 (597.9–821.3)	15.7 (−4.2–41.2)
Southeast Asia	73.6 (67.8–79.7)	10,474.6 (9718.9–11,301.7)	−11.7 (−13.5–−10.2)	170 (148.9–190.5)	28.5 (24.8–31.9)	−8.3 (−14.1–−3)	5703.3 (5028.7–6329)	948.4 (838.7–1090.4)	6 (−10.1–23.7)
Central Asia	9.4 (8.7–10)	10,698.2 (10,022.9–11,348.1)	−0.8 (−1.9–0.1)	9.4 (8.3–10.5)	12.1 (10.7–13.5)	2.4 (−22.2–33.4)	427.6 (377.5–490.7)	493.2 (436–565.5)	90.8 (82.4–100)
Central Europe	11.5 (10.8–12.1)	6205.2 (5841–6596.2)	3.1 (0.5–5.2)	21.6 (19.4–24)	9.4 (8.4–10.5)	−22.5 (−39.2–−5.5)	540 (472.2–612.7)	266.9 (233.4–306.2)	−13.4 (−23–−0.9)
Eastern Europe	27.3 (25.4––29.3)	9266.3 (8619.4–9989.5)	−0.6 (−2.5–1.6)	17.8 (16.1–19.9)	5.2 (4.7–5.8)	29 (18.4–37.6)	642.5 (562.9–727)	204.7 (180.4–232.1)	2.6 (−2.5–7.1)
Australasia	2.8 (2.6–3)	5910 (5548.2–6301.6)	−7.5 (−8.5–−6.6)	6 (5–6.5)	9.6 (8.2–10.5)	65 (26.9–89.5)	113.7 (101.5–125.3)	216.6 (193.2–239.2)	−−30.1 (−41.9–−18.5)
Southern Latin America	4.9 (4.5–5.2)	5970.4 (5543.1–6415)	−3.7 (−4.7–−2.8)	21.6 (19.5–23)	23.8 (21.6–25.4)	−10.4 (−18.3–-1.7)	441.1 (411.3–467.9)	515.2 (481.8–546.8)	−25.4 (−29.1–−22.3)
High-income Asia Pacific	29.3 (27.3–31)	7920.7 (7393.7–8457.4)	−2.6 (−4.1–−1)	63.5 (50.4–71)	9.7 (8.1–10.7)	147 (139.1–155.4)	63.5 (50.4–71)	9.7 (8.1–10.7)	147 (139.11–55.4)
Andean Latin America	3.7 (3.5–4)	5946.1 (5524.8–6371.5)	2.7 (0.7–4.4)	21.6 (17.9–25.8)	37.7 (31.3–44.9)	32 (7.7–63.3)	524.2 (434.4–625)	872.4 (723.5–1037.9)	−1.3 (−7.9–7.1)
Caribbean	3.5 (3.2–3.7)	6633 (6181.3–7112.4)	1 (−0.2–2.4)	13.9 (12.1–16.2)	25.8 (22.4–30.1)	13.7 (6.1–20.4)	385.3 (331.6–454.1)	735.8 (631–867.9)	3.2 (−1.7–8.5)
Western Europe	41.6 (39.1–43.9)	5226.2 (4924.4–5544.2)	0.7 (−0.3–1.8)	133.5 (109.6–148.2)	10.7 (8.9–11.8)	45.9 (33.3–59.9)	2361.3 (2067.1–2638.2)	241.7 (209.8–271.1)	51.3 (37.2–68)
High-income North America	42.5 (39.4–45.2)	7434.7 (6959.3–7911.4)	−2.4 (−3.4–−1.4)	143.7 (124.8–154.8)	20.6 (18.1–22)	−22.8 (−28.7–−18.6)	3121 (2864.2–3351.6)	508.8 (467–545.4)	0.2 (−13.2–13.9)
Tropical Latin America	19.3 (18–20.7)	7576.2 (7081–8107.5)	2.4 (0.7–4)	46.9 (42.5–49.4)	18.8 (17–19.9)	5 (−−0.5–9.1)	1311.9 (1220.7–1401.2)	517 (480.6–552.1)	12.5 (−2.1–26.6)
Central Latin America	22.1 (20.7–23.4)	8642.9 (8089.3–9163.7)	0.5 (−1.3–2.1)	104.4 (94.2–116.5)	42.4 (38.3–47)	−5.5 (−18.6–8.4)	2993.8 (2691.7–3371.7)	1171.1 (1054.8–1316.3)	−15.9 (−20.3–−−11.5)
South Asia	158.8 (147.2–171.6)	9565.3 (8903.7–10,265.7)	−2.9 (−5.7–0.1)	226 (192.5–264.2)	16.5 (14–19.3)	37.6 (18.9–57.5)	8443.3 (7372.3–9681.8)	540.6 (473.8–620.4)	43.6 (19.2–61.7)
North Africa and Middle East	49.7 (45.8–53.6)	9180 (8523.5–9830.1)	2.5 (1.7–3.3)	144.7 (126.2–162.7)	37.7 (32.7–42.4)	141.9 (101.3–186.6)	3926 (3427.2–4413.6)	846.6 (747.2–948)	30.2 (12.9–49.6)
Central Sub-Saharan Africa	6.8 (6.4–7.3)	9165.2 (8640.8–9709.7)	0.2 (−0.9–1.2)	21 (16.1–27.5)	43.7 (33.3–56.3)	25.2 (−11.7–78.6)	783.7 (622.6–996.5)	1124.7 (899–1436.1)	52.5 (37.6–68.7)
Eastern Sub-Saharan Africa	15 (13.7–16.4)	5821.3 (5404.5–6293.6)	3.2 (0.7–6.1)	60.9 (53.8–69.9)	40.1 (35.6–46)	17.7 (−−7.6–41.4)	2047.6 (1792–2374.2)	846.3 (753.1–940.5)	10.4 (0.8–17.8)
Western Sub-Saharan Africa	22.5 (20.9–24.2)	8324.3 (7782.3–8871)	0.5 (−0.6–1.8)	64.8 (53.5–76.1)	36.4 (31.1–42.8)	24.5 (10.2–33.5)	2437.3 (1992.7–2893.6)	930.7 (788.4–1081.1)	11.4 (−23.9–35.5)
Southern Sub-Saharan Africa	6 (5.5–6.4)	9037.9 (8440.6–9647.1)	2 (0.1–3.9)	1527.6 (1389.4–1638.9)	18.5 (16.7–19.9)	24.6 (2.4–43.3)	44,453.7 (40,840.8–48,508.5)	529.6 (486.2–577.4)	−22.1 (−28–−14.7)

ASR = age standardized rate, CKD = chronic kidney disease, DALY = disability-adjusted life year, GBD = Global Burden of Disease, UI = uncertainty interval.

### 3.2. Regional level

In 2021, the highest age-standardized prevalence rates of CKD were observed in Central Asia, Southeast Asia, South Asia, and Eastern Europe, while the lowest rates were reported in Western Europe and Eastern Sub-Saharan Africa (Table 1). Similarly, the highest mortality rates were found in Central Sub-Saharan Africa, Central Latin America, and Eastern Sub-Saharan Africa, and the lowest rates were found in Eastern Europe, Central Europe, Australasia, and the high-income Asia Pacific (Table 1). In terms of DALYs, the highest rates were observed in Central Latin America and Central Sub-Saharan Africa, while the lowest rates were reported in Eastern Europe, Australasia, high-income Asia Pacific, and Western Europe (Table 1). Figures S1, S2 and S3, Supplemental Digital Content 3 display the age-standardized prevalence, mortality, and DALY rates of CKD by sex for all regions in the 2021 GBD study.

From 1990 to 2021, the most significant increases in age-standardized CKD prevalence were observed in Eastern Sub-Saharan Africa and Central Europe, while the greatest decreases were seen in Southeast Asia and Australasia (Table 1). During the same period, the largest increases in age-standardized CKD mortality were reported in high-income Asia Pacific, North Africa, and the Middle East, while the largest decreases were observed in high-income North America and Central Europe (Table 1). From 1990 to 2021, the largest increases in age-standardized DALY rates occurred in Central Asia, Central Sub-Saharan Africa, and Western Europe, whereas the largest decreases were seen in Australasia, Southern Latin America, and Southern Sub-Saharan Africa (Table 1). Figures S4, S5 and S6, Supplemental Digital Content 4 show the percentage changes in age-standardized CKD point prevalence, mortality, and DALY rates by sex from 1990 to 2021.

The number of prevalent CKD cases increased from 351 million in 1990 to 673.7 million in 2021. East Asia, South Asia, and Southeast Asia had the highest number of prevalent cases in 1990, and these regions continued to have the highest number in 2021 (Table S3, Supplemental Digital Content 5). CKD-related deaths rose from 550,000 in 1990 to 1.53 million in 2021, with East Asia, South Asia, and Southeast Asia experiencing the highest fatalities in 2021 (Table S4, Supplemental Digital Content 6). DALYs attributable to CKD increased from 20.74 million in 1990 to 44.45 million in 2021, with East Asia, South Asia, and Southeast Asia contributing the most in 2021 (Table S5, Supplemental Digital Content 7).

### 3.3. National level

**Figure 1.**
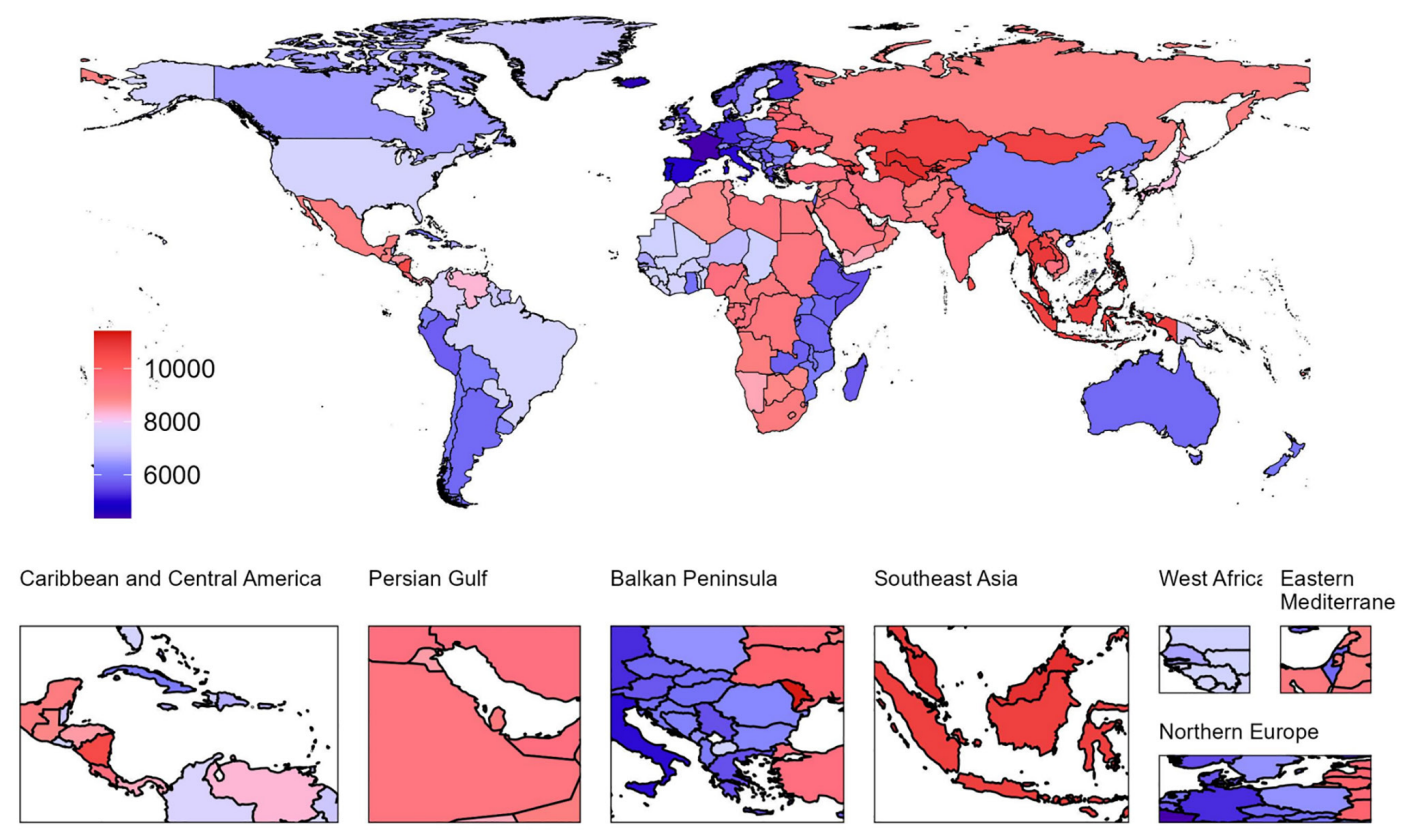
Age-standardized prevalence rate of CKD per 100,000 population by country in 2021. CKD = chronic kidney disease.

**Figure 2.**
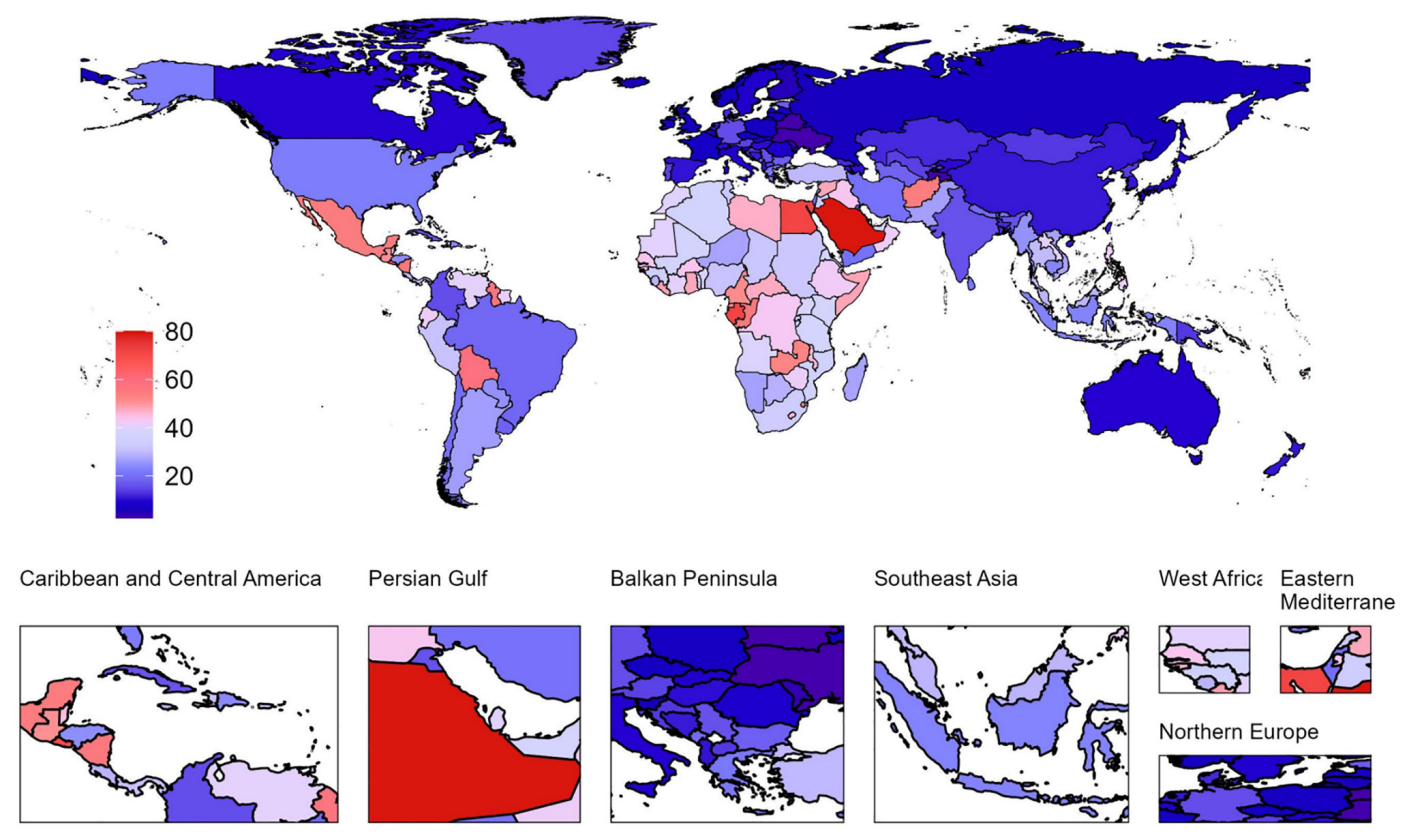
Age-standardized mortality rate of CKD per 100,000 population by country in 2021. CKD = chronic kidney disease.

In 2021, the national age-standardized prevalence rates of CKD ranged from 4368.8 to 11,411.6 per 100,000 population. Mauritius, the Republic of Moldova, and Uzbekistan had the highest rates, while France, Iceland, and Portugal had the lowest estimates (Fig. 1 and Table S3, Supplemental Digital Content 5). National mortality rates ranged from 2.4 to 80.1 per 100,000 population. Mauritius, Saudi Arabia, and American Samoa had the highest mortality rates, while Belarus, Ukraine, and Tajikistan had the lowest estimates (Fig. 2 and Table S4, Supplemental Digital Content 6). National DALY rates for CKD ranged from 145.1 to 2196.1 per 100,000 population. Mauritius, American Samoa, and El Salvador had the highest DALY rates, while Belarus, Iceland, San Marino, and Finland had the lowest rates (Fig. S7, Supplemental Digital Content 8 and Table S5, Supplemental Digital Content 7).

From 1990 to 2021, the largest increases in age‑standardized prevalence rates were observed in Guatemala, Cameroon, and the United Republic of Tanzania, while the largest decreases were seen in China, the Republic of Korea, and the United Kingdom (Table S3, Supplemental Digital Content 5). During the same period, the largest increases in ASMRs occurred in Ukraine, Belarus, and Uzbekistan, and the largest decreases were recorded in Poland, Kuwait, and Cyprus (Table S4, Supplemental Digital Content 6). For age‑standardized DALY rates, the largest increases were in El Salvador, Armenia, and Lesotho, while the largest decreases were in Kuwait, Poland, and the Republic of Korea (Table S5, Supplemental Digital Content 7).

### 3.4. Age and gender patterns

**Figure 3.**
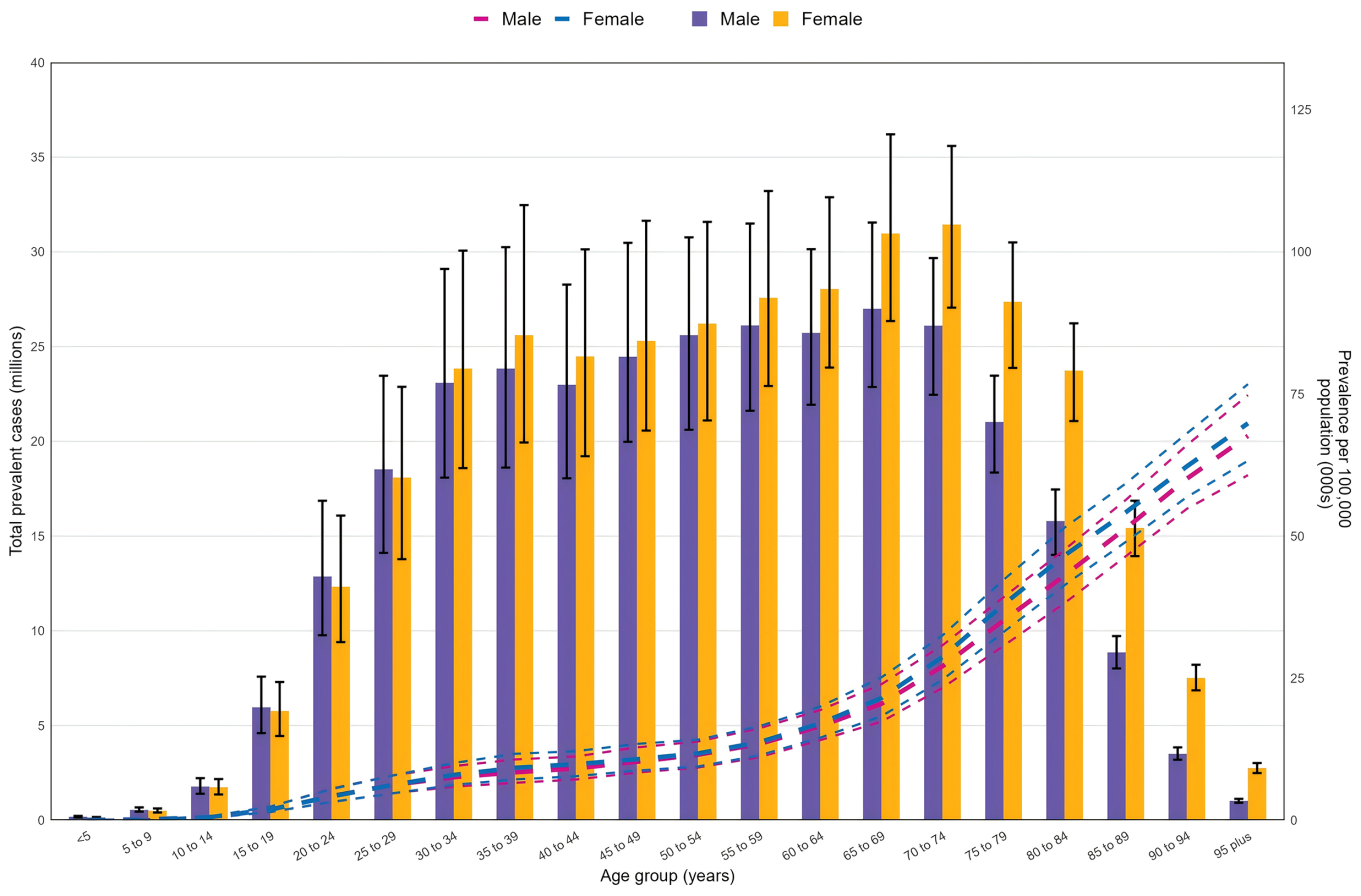
Global age- and sex-specific total prevalence cases and crude prevalence rate of CKD in 2021. CKD = chronic kidney disease.

In 2021, global CKD prevalence started rising in the 10 to 14 age group and peaked in individuals aged 95 and above. The number of prevalent cases peaked in the 70 to 74 age group and then declined with age. The number of CKD cases was higher among females in the 70 to 74 age group, and CKD was more common in females aged 74 and above (Fig. 3). In 2021, global CKD mortality rates were highest in the oldest age group (95 plus) and higher among females aged 84 and above. The highest number of male deaths occurred in the 70 to 74 age group, after which the number of deaths decreased with age. Among females, the highest number of deaths occurred in the 85 to 89 age group, followed by a decline with increasing age (Fig. S8, Supplemental Digital Content 9). In 2021, global DALY rates for CKD were highest in the oldest age group (95 plus). Additionally, the number of DALYs peaked in the 65 to 69 age group and subsequently declined with increasing age (Fig. S9, Supplemental Digital Content 10).

### 3.5. Association with the SDI

**Figure 4.**
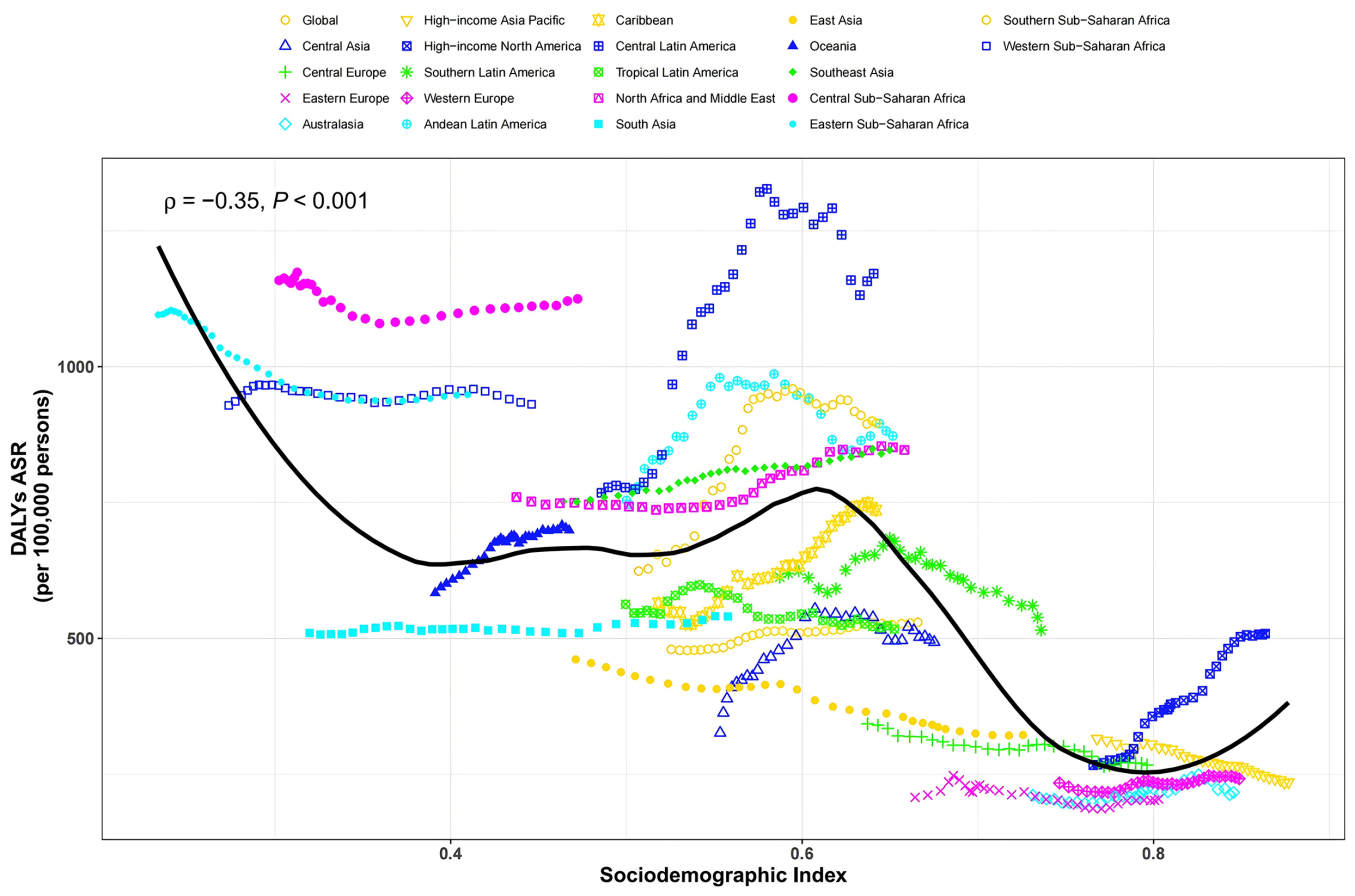
Age-standardized DALY rate of CKD by SDI level across 21 GBD regions, 1990 to 2021. CKD = chronic kidney disease, DALY = disability-adjusted life year, GBD = Global Burden of Disease, SDI = sociodemographic index.

At the regional level, we observed a W-shaped association between the SDI and age-standardized DALYs rates for CKD from 1990 to 2021. The age-standardized DALYs rate fluctuated with changes in SDI, peaking around an SDI of 0.1, then declining, and slowly increasing again between 0.4 and 0.6. After 0.6, the age-standardized DALY rate decreased exponentially with SDI until reaching approximately 0.8, after which it began to rise again. From 1990 to 2021, DALY rates in Central Sub-Saharan Africa, Central Latin America, Andean Latin America, North Africa, the Middle East, and Southeast Asia were higher than expected based on their SDI. In contrast, the burden in South Asia, Tropical Latin America, East Asia, Western Europe, Eastern Europe, and Australasia was lower than expected during the same period (Fig. 4).

At the national level, in 2021, the burden of CKD increased with socioeconomic development, peaking at an SDI of approximately 0.60, but then declined as the SDI reached around 0.93 (Fig. S10, Supplemental Digital Content 11). The burden in countries and regions such as Mauritius, American Samoa, El Salvador, Nauru, and Saudi Arabia was significantly higher than expected, whereas the burden in France, Finland, Iceland, and Italy was much lower than expected (Fig. S10, Supplemental Digital Content 11).

### 3.6. Risk factors

**Figure 5.**
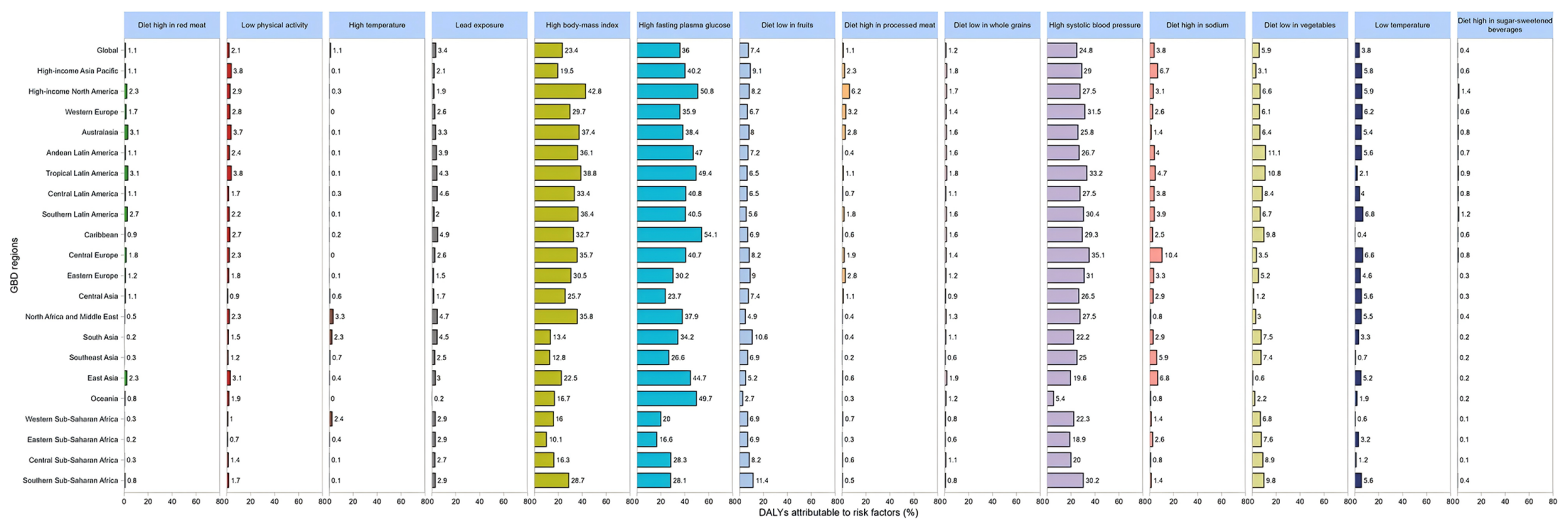
Percentage of CKD DALYs attributable to each risk factor across 21 GBD regions in 2021. CKD = chronic kidney disease, DALY = disability-adjusted life year, GBD = Global Burden of Disease.

The proportion of CKD-related DALYs attributable to individual risk factors varied across GBD regions. Globally, high fasting plasma glucose, high systolic blood pressure, and high body mass index were the leading contributors to CKD-related DALYs, accounting for 36.0%, 24.8%, and 23.4%, respectively (Fig. 5). The proportion of CKD DALYs caused by these 3 risk factors was higher among females (Figs. S11 and S12, Supplemental Digital Content 12).

The proportion of CKD-related DALYs attributable to individual risk factors also varied by age group. The proportion of DALYs attributable to high fasting plasma glucose increased with age until the 75 to 79 age group, then declined. The highest proportion of DALYs attributable to high body mass index occurred in the 95-plus age group. Additionally, CKD DALYs attributable to high systolic blood pressure increased with age, peaking in the 80 to 84 age group, where it accounted for 34.8% (Fig. S13, Supplemental Digital Content 13). Figures S14 and S15, Supplemental Digital Content 14 show the proportion of CKD-related DALYs caused by individual risk factors for males and females, respectively.

## 4. Discussion

Based on the data from this study, the global burden of CKD has significantly increased over the past few decades, consistent with the description by Deng et al.^[15]^ A notable paradox emerged: Between 1990 and 2021, the age‑standardized prevalence and DALY rates decreased, while the ASMR increased. Correspondingly, the absolute burden of these diseases increased continuously over this period. The shift in disease burden over this period is partly driven by demographic changes, namely a growing and aging global population, increasing the overall number of people susceptible to illness.^[16]^ Regional analysis showed a higher burden of CKD in Central Asia, Southeast Asia, and South Asia, while high-income countries such as Western Europe and North America had relatively lower burdens. The W‑shaped association between the SDI and CKD burden further supports these regional patterns: low‑SDI regions had higher‑than‑expected burden due to resource constraints, while high‑SDI regions achieved lower burden through effective health systems despite high metabolic risk prevalence.^[17]^

Multiple studies have highlighted the continuous increase in the global CKD burden, especially in low-income countries, where CKD has become a major public health issue.^[7,18]^ Compared to developed countries, the burden of CKD is more severe in developing countries, primarily due to a lack of healthcare resources, insufficient early disease screening, and inadequate control of major risk factors such as hypertension and diabetes. This study confirms that while some developed countries have reduced CKD prevalence through better diabetes and blood pressure management, low‑income countries face higher mortality rates due to delayed diagnosis and treatment.^[19]^ To close this gap, key policy actions include the integration of hypertension and diabetes screening into primary care, the task‑shifting of routine management to community health workers, and the provision of affordable generic medications.

This study highlights major regional disparities in CKD burden, with Central, Southeast, and South Asia showing notably higher prevalence and mortality rates. This finding is consistent with the study by Aashima et al, which identified Central Asia as having one of the highest CKD burdens globally, a trend closely linked to the high prevalence of hypertension and diabetes in the region.^[20]^ One study also noted that the lack of resources in low-income countries weakens early CKD screening capabilities, leading to higher mortality rates as many patients do not receive timely treatment.^[21]^ To address these disparities, future policy efforts should prioritize the allocation of basic medical infrastructure and equipment to resource‑limited regions.

Globally, diabetes, hypertension, and obesity are the primary risk factors driving the increase in CKD burden. Multiple studies have pointed out that the global prevalence of diabetes and hypertension has worsened the CKD burden, particularly in developing countries. These findings are consistent with the conclusions drawn by Li et al and Xie et al from their earlier analyses of CKD risk factors in previous GBD datasets.^[22,23]^ However, our study provides novel insights by extending the spatiotemporal scope and examining sex-based disparities in risk factors, offering enhanced practical significance for guiding CKD prevention. Hyperglycemia caused by diabetes accelerates CKD progression through chronic inflammatory responses and glomerular damage, while hypertension leads to increased glomerular pressure, resulting in microvascular disease in the kidneys and subsequent decline in kidney function.^[12,24]^ Additionally, obesity has become a significant driver of the rising CKD burden. Research has shown that obesity not only increases the risk of diabetes and hypertension but also directly affects kidney function, accelerating CKD progression.^[25]^ Given that the burden of CKD is largely due to modifiable risk factors, focusing on their control presents a key and feasible strategy for its reduction. Several initiatives have been developed to reduce exposure to these risk factors. For example, in 2013, the World Health Organization approved the Global Action Plan for the Prevention and Control of NCDs, aimed at reducing exposure to risk factors for NCDs, including CKD, with a focus on reducing hypertension, hyperglycemia, and obesity. Specific strategies include promoting healthy diets (reducing sodium and sugar intake), encouraging physical activity through community programs, and advocating for policies that limit the consumption of processed and sugary foods.^[26]^

This study demonstrates significant differences in the age and gender distribution of CKD, with higher prevalence in women over the age of 74 compared to men. This finding is consistent with another study, which indicated that postmenopausal women experience a decline in kidney protection due to reduced estrogen levels, thereby accelerating CKD progression.^[27]^ Additionally, the elderly population, especially those aged 75 and above, bears the highest burden of CKD. Globally, aging remains one of the primary drivers of the increasing CKD burden. As life expectancy continues to rise worldwide, the incidence of CKD in older adults is expected to grow. Studies have shown that older adults are more susceptible to chronic diseases such as diabetes and hypertension, which are major drivers of CKD. Furthermore, the natural decline in kidney function with age makes older adults more vulnerable to CKD.^[28]^ Future global health policies need to prioritize early screening for CKD and the management of risk factors such as diabetes and hypertension in the elderly population, for example, by incorporating annual urine albumin testing into routine geriatric care and ensuring access to affordable antihypertensive and glucose‑lowering medications.^[29,30]^

The rise in the global burden of CKD in 2021 may be partly due to the coronavirus disease 2019 pandemic. According to related studies, CKD patients have a significantly higher mortality rate from coronavirus disease 2019 compared to the general population.^[31]^ A meta-analysis indicated that the global reorganization and disruption of healthcare services had an adverse impact on CKD patients, particularly in low- and middle-income countries.^[32]^ A study pointed out that the redistribution of healthcare resources during the pandemic led to delays in dialysis and kidney transplant surgeries for CKD patients, which may have accelerated disease progression and increased mortality risk.^[33]^ The pandemic also impacted patient health behaviors, with home isolation and reduced physical activity exacerbating issues like obesity and hypertension, indirectly contributing to the rise in the CKD burden.^[34]^

To address the rising global CKD burden, countries should develop and implement multitiered prevention and management strategies. Early screening and intervention are key to preventing CKD progression. The literature suggests that early diagnosis and active intervention in CKD can significantly reduce the risk of progression to end-stage renal disease.^[35]^ Second, personalized management plans should be implemented for high-risk populations, such as those with diabetes, hypertension, or obesity, to control their underlying risk factors. Furthermore, public health policies should focus particularly on low- and middle-income countries to ensure that CKD patients in these regions have access to essential healthcare services and treatments.

However, our study has several limitations. First, it is critical to recognize a constraint inherent to our data source. Kidney dysfunction contributes to mortality both directly by causing progressive kidney disease and indirectly via its role in increasing the risk of fatal cardiovascular events. Because the GBD framework assesses causes of death and risk factors in separate analytical streams, our study (which analyzes CKD as a cause of death) does not quantify the burden attributable to impaired kidney function in its role as a risk factor.^[10]^ Second, our study relies on GBD modeled estimates, which are subject to limitations arising from sparse primary data, particularly in low‑ and middle‑income countries. Limited data availability necessitates the use of statistical models and extrapolations, which in turn produce wider confidence intervals and greater regional uncertainty. Moreover, the GBD often relies on single measurements from cross‑sectional studies, whereas the clinical definition of CKD requires persistent dysfunction, potentially overestimating early‑stage prevalence in these settings.^[36]^ Third, some risk factors, such as genetic susceptibility, though rare, were not accounted for in our estimates. Finally, differences in health systems, screening, and reporting mechanisms across regions may introduce bias when directly comparing CKD burden between different areas.

## 5. Conclusion

This study reveals a continuing upward trend in the global burden of CKD, particularly in low- and middle-income countries. While some high-income countries have reduced CKD prevalence and mortality through effective early screening and chronic disease management strategies, the absolute global burden continues to rise. In the future, global health systems will need to strengthen early screening for CKD and manage its major risk factors to address the anticipated increase in CKD burden.

## Acknowledgments

We thank the institute for Health Metrics and Evaluation for sharing valuable GBD data.

## Author contributions

**Conceptualization:** Wuyuan Tao, Liuxia Deng.

**Data curation:** Chunlan Chen.

**Formal analysis:** Jinfeng Zhang, Xiangyue Jiang.

**Software:** Jinfeng Zhang.

**Writing – original draft:** Wuyuan Tao, Xiangyue Jiang.

**Writing – review & editing:** Liuxia Deng.









































## References

[1] Cockwell P Fisher LA. The global burden of chronic kidney disease. Lancet. 2020;395:662–4. 32061314 10.1016/S0140-6736(19)32977-0

[2] Francis A Harhay MN Ong ACM. Chronic kidney disease and the global public health agenda: an international consensus. Nat Rev Nephrol. 2024;20:473–85. 38570631 10.1038/s41581-024-00820-6

[3] Scurt FG Ganz MJ Herzog C Bose K Mertens PR Chatzikyrkou C. Association of metabolic syndrome and chronic kidney disease. Obes Rev. 2024;25:e13649. 37783465 10.1111/obr.13649

[4] Jadoul M Aoun M Imani MM. The major global burden of chronic kidney disease. Lancet Glob Health. 2024;12:e342–3. 38365398 10.1016/S2214-109X(24)00050-0

[5] Evans M Lewis RD Morgan AR. A narrative review of chronic kidney disease in clinical practice: current challenges and future perspectives. Adv Ther. 2022;39:33–43. 34739697 10.1007/s12325-021-01927-z PMC8569052

[6] Kovesdy CP. Epidemiology of chronic kidney disease: an update 2022. Kidney Int Suppl (2011). 2022;12:7–11. 35529086 10.1016/j.kisu.2021.11.003 PMC9073222

[7] Tonelli M Dickinson JA. Early detection of CKD: implications for low-income, middle-income, and high-income countries. J Am Soc Nephrol. 2020;31:1931–40. 32839279 10.1681/ASN.2020030277 PMC7461685

[8] Burgos-Calderón R Depine SA Aroca-Martínez G. Population kidney health. a new paradigm for chronic kidney disease management. Int J Environ Res Public Health. 2021;18:6786. 34202623 10.3390/ijerph18136786 PMC8297314

[9] GBD 2021 Risk Factors Collaborators. Global burden and strength of evidence for 88 risk factors in 204 countries and 811 subnational locations, 1990-2021: a systematic analysis for the Global Burden of Disease Study 2021. Lancet. 2024;403:2162–203. 38762324 10.1016/S0140-6736(24)00933-4 PMC11120204

[10] GBD 2021 Diseases and Injuries Collaborators. Global incidence, prevalence, years lived with disability (YLDs), disability-adjusted life-years (DALYs), and healthy life expectancy (HALE) for 371 diseases and injuries in 204 countries and territories and 811 subnational locations, 1990-2021: a systematic analysis for the Global Burden of Disease Study 2021. Lancet. 2024;403:2133–61. 38642570 10.1016/S0140-6736(24)00757-8 PMC11122111

[11] GBD 2019 Mental Disorders Collaborators. Global, regional, and national burden of 12 mental disorders in 204 countries and territories, 1990-2019: a systematic analysis for the Global Burden of Disease Study 2019. Lancet Psychiatry. 2022;9:137–50. 35026139 10.1016/S2215-0366(21)00395-3 PMC8776563

[12] Burnier M Damianaki A. Hypertension as cardiovascular risk factor in chronic kidney disease. Circ Res. 2023;132:1050–63. 37053276 10.1161/CIRCRESAHA.122.321762

[13] GBD 2017 Risk Factor Collaborators. Global, regional, and national comparative risk assessment of 84 behavioural environmental and occupational, and metabolic risks or clusters of risks for 195 countries and territories, 1990-2017: a systematic analysis for the Global Burden of Disease Study 2017. Lancet. 2018;392:1923–94. 30496105 10.1016/S0140-6736(18)32225-6 PMC6227755

[14] Lu X Wang R Li J. Exposure-lag response of surface net solar radiation on lung cancer incidence: a global time-series analysis. Transl Lung Cancer Res. 2024;13:2524–37. 39507019 10.21037/tlcr-24-125 PMC11535824

[15] Deng L Guo S Liu Y. Global, regional, and national burden of chronic kidney disease and its underlying etiologies from 1990 to 2021: a systematic analysis for the Global Burden of Disease Study 2021. BMC Public Health. 2025;25:636. 39962443 10.1186/s12889-025-21851-z PMC11831764

[16] Guo J Jiao W Xia S. The global, regional, and national patterns of change in the burden of chronic kidney disease from 1990 to 2021. BMC Nephrol. 2025;26:136. 40082779 10.1186/s12882-025-04028-z PMC11907979

[17] Guo J Liu Z Wang P. Global, regional, and national burden inequality of chronic kidney disease, 1990-2021: a systematic analysis for the global burden of disease study 2021. Front Med (Lausanne). 2024;11:1501175. 39882527 10.3389/fmed.2024.1501175 PMC11774877

[18] GBD Chronic Kidney Disease Collaboration. Global, regional, and national burden of chronic kidney disease, 1990-2017: a systematic analysis for the Global Burden of Disease Study 2017. Lancet. 2020;395:709–33. 32061315 10.1016/S0140-6736(20)30045-3 PMC7049905

[19] Luyckx VA Cherney DZI Bello AK. Preventing CKD in Developed Countries. Kidney Int Rep. 2020;5:263–77. 32154448 10.1016/j.ekir.2019.12.003 PMC7056854

[20] Aashima Nanda M Sharma R Jani C. The burden of chronic kidney disease in Asia, 1990-2019: examination of estimates from global burden of disease 2019 study. Nephrology (Carlton). 2022;27:610–20. 35506615 10.1111/nep.14051

[21] Demir E Turkmen A Sever MS. Risk factors, pathogenesis, presentation and management of BK virus infection in kidney transplantation. Nephrol Dial Transplant. 2021;36:985–7. 34047340 10.1093/ndt/gfz214

[22] Li MJ Liu HY Zhang YQ Li SR Zhang JH Li R. Global burden of chronic kidney disease and its attributable risk factors (1990-2021): an analysis based on the global burden of disease study. Front Endocrinol (Lausanne). 2025;16:1563246. 40678313 10.3389/fendo.2025.1563246 PMC12267038

[23] Xie D Ma T Cui H. Global burden and influencing factors of chronic kidney disease due to type 2 diabetes in adults aged 20-59 years, 1990-2019. Sci Rep. 2023;13:20234. 37981642 10.1038/s41598-023-47091-y PMC10658077

[24] Kalyesubula R Conroy AL Calice-Silva V. Screening for kidney disease in low- and middle-income countries. Semin Nephrol. 2022;42:151315. 37001337 10.1016/j.semnephrol.2023.151315

[25] Hall JE do Carmo JM da Silva AA Wang Z Hall ME. Obesity, kidney dysfunction and hypertension: mechanistic links. Nat Rev Nephrol. 2019;15:367–85. 31015582 10.1038/s41581-019-0145-4 PMC7278043

[26] World Health Organization. Global action plan for the prevention and control of noncommunicable diseases 2013-2020. WHO; 2013

[27] Chesnaye NC Carrero JJ Hecking M Jager KJ. Differences in the epidemiology, management and outcomes of kidney disease in men and women. Nat Rev Nephrol. 2024;20:7–20. 37985869 10.1038/s41581-023-00784-z

[28] Kishi S Kadoya H Kashihara N. Treatment of chronic kidney disease in older populations. Nat Rev Nephrol. 2024;20:586–602. 38977884 10.1038/s41581-024-00854-w

[29] Chesnaye NC Ortiz A Zoccali C Stel VS Jager KJ. The impact of population ageing on the burden of chronic kidney disease. Nat Rev Nephrol. 2024;20:569–85. 39025992 10.1038/s41581-024-00863-9

[30] Portilla Franco ME Puente-García A Pérez-Belmonte LM Pérez N Ellingson S Tornero F. Comprehensive management of chronic kidney disease in the old population. Curr Med Res Opin. 2025;41:1451–64. 40888118 10.1080/03007995.2025.2551217

[31] Iryaningrum MR Supriyadi R Lawrensia S Henrina J Soetedjo NNM. Diabetes and mortality among patients with chronic kidney disease and COVID-19: a systematic review, meta-analysis, and meta-regression. Indian J Nephrol. 2022;32:327–33. 35967541 10.4103/ijn.ijn_293_21 PMC9364990

[32] Naicker S Yang CW Hwang SJ Liu B-C Chen J-H Jha V. The novel coronavirus 2019 epidemic and kidneys. Kidney Int. 2020;97:824–8. 32204907 10.1016/j.kint.2020.03.001 PMC7133222

[33] Geetha D Kronbichler A Rutter M. Impact of the COVID-19 pandemic on the kidney community: lessons learned and future directions. Nat Rev Nephrol. 2022;18:724–37. 36002770 10.1038/s41581-022-00618-4 PMC9400561

[34] Filgueira TO Castoldi A Santos LER. The relevance of a physical active lifestyle and physical fitness on immune defense: mitigating disease burden, with focus on COVID-19 consequences. Front Immunol. 2021;12:587146. 33613573 10.3389/fimmu.2021.587146 PMC7892446

[35] Yan Z Wang G Shi X. Advances in the progression and prognosis biomarkers of chronic kidney disease. Front Pharmacol. 2021;12:785375. 34992536 10.3389/fphar.2021.785375 PMC8724575

[36] De Broe ME Gharbi MB Zamd M Elseviers M. Why overestimate or underestimate chronic kidney disease when correct estimation is possible? Nephrol Dial Transplant. 2017;32(suppl_2): ii136–ii141. 28380639 10.1093/ndt/gfw267

