# Supplementary material for: Burden of chronic kidney disease and its attributable risk factors in 204 countries and territories, 1990–2021: Results from the Global Burden of Disease study 2021
Source: Medicine (Baltimore). 2026 Aug 7;105(32):e50111. doi: 10.1097/MD.0000000000050111 (PMC13456769; doi:10.1097/MD.0000000000050111)
Supplement: Supplementary file 1 [file medi-105-e50111-s001.docx]

**Table S1. CKD eGFR Equations**

| Equation | Formula |
| --- | --- |
| CKD-EPI  2009 | eGFR = 141 x min (S_Cr_/κ, 1)^α^ x max (S_Cr_  /κ, 1)-^1.209^ x 0.993^Age^ x 1.018 [if female] x 1.159 [if Black]  Κ is 0.7 for females and 0.9 for males  α is -0.329 for females and -0.411 for males, where min indicates the minimum of Scr/κ or 1, and max indicates the maximum of Scr/κ or 1 |
| Schwartz | eGFR = 0.413 x (height/Scr) if height is expressed in centimetres  OR  41.3 x (height/Scr) if height is expressed in metres |
