# Supplementary material for: Burden of chronic kidney disease and its attributable risk factors in 204 countries and territories, 1990–2021: Results from the Global Burden of Disease study 2021
Source: Medicine (Baltimore). 2026 Aug 7;105(32):e50111. doi: 10.1097/MD.0000000000050111 (PMC13456769; doi:10.1097/MD.0000000000050111)
Supplement: Supplementary file 2 [file medi-105-e50111-s002.docx]

**Table S2.** GBD 2021 Classification and Codes of Risk Factors

| REI ID | REI Name | Parent ID | Parent Name | Level | Sort Order | REI Type ID | REI Type |
| --- | --- | --- | --- | --- | --- | --- | --- |
| 169 | All risk factors | 169 | All risk factors | 0 | 1 | 1 | Risk |
| 171 | Etiologies | 171 | Etiologies | 0 | 1 | 2 | Etiology |
| 191 | Impairments | 191 | Impairments | 0 | 1 | 3 | Impairment |
| 362 | Injuries | 362 | Injuries | 0 | 1 | 4 | Injury n-codes |
| 202 | Environmental/occupational risks | 169 | All risk factors | 1 | 2 | 1 | Risk |
| 414 | Acinetobacter baumannii | 171 | Etiologies | 1 | 2 | 2 | Etiology |
| 192 | Anemia | 191 | Impairments | 1 | 2 | 3 | Impairment |
| 298 | Amputations | 362 | Injuries | 1 | 2 | 4 | Injury n-codes |
| 82 | Unsafe water, sanitation, and handwashing | 202 | Environmental/occupational risks | 2 | 3 | 1 | Risk |
| 185 | Adenovirus | 171 | Etiologies | 1 | 3 | 2 | Etiology |
| 205 | Mild anemia | 192 | Anemia | 2 | 3 | 3 | Impairment |
| 250 | Amputation of lower limbs, bilateral | 298 | Amputations | 2 | 3 | 4 | Injury n-codes |
| 83 | Unsafe water source | 82 | Unsafe water, sanitation, and handwashing | 3 | 4 | 1 | Risk |
| 182 | Aeromonas | 171 | Etiologies | 1 | 4 | 2 | Etiology |
| 432 | Moderate and Severe Anemia | 192 | Anemia | 2 | 4 | 3 | Impairment |
| 261 | Amputation of upper limbs, bilateral | 298 | Amputations | 2 | 4 | 4 | Injury n-codes |
| 84 | Unsafe sanitation | 82 | Unsafe water, sanitation, and handwashing | 3 | 5 | 1 | Risk |
| 620 | Aspergillus spp. | 171 | Etiologies | 1 | 5 | 2 | Etiology |
| 206 | Moderate anemia | 432 | Moderate and Severe Anemia | 3 | 5 | 3 | Impairment |
| 272 | Amputation of fingers (excluding thumb) | 298 | Amputations | 2 | 5 | 4 | Injury n-codes |
| 238 | No access to handwashing facility | 82 | Unsafe water, sanitation, and handwashing | 3 | 6 | 1 | Risk |
| 178 | Campylobacter | 171 | Etiologies | 1 | 6 | 2 | Etiology |
| 207 | Severe anemia | 432 | Moderate and Severe Anemia | 3 | 6 | 3 | Impairment |
| 283 | Amputation of lower limb, unilateral | 298 | Amputations | 2 | 6 | 4 | Injury n-codes |
| 85 | Air pollution | 202 | Environmental/occupational risks | 2 | 7 | 1 | Risk |
| 631 | Candida spp. | 171 | Etiologies | 1 | 7 | 2 | Etiology |
| 193 | Epilepsy | 191 | Impairments | 1 | 7 | 3 | Impairment |
| 293 | Amputation of upper limb, unilateral | 298 | Amputations | 2 | 7 | 4 | Injury n-codes |
| 380 | Particulate matter pollution | 85 | Air pollution | 3 | 8 | 1 | Risk |
| 415 | Chlamydia spp | 171 | Etiologies | 1 | 8 | 2 | Etiology |
| 194 | Guillain-BarrÃ© syndrome | 191 | Impairments | 1 | 8 | 3 | Impairment |
| 294 | Amputation of thumb | 298 | Amputations | 2 | 8 | 4 | Injury n-codes |
| 86 | Ambient particulate matter pollution | 380 | Particulate matter pollution | 4 | 9 | 1 | Risk |
| 173 | Cholera | 171 | Etiologies | 1 | 9 | 2 | Etiology |
| 195 | Hearing loss | 191 | Impairments | 1 | 9 | 3 | Impairment |
| 295 | Amputation of toe/toes | 298 | Amputations | 2 | 9 | 4 | Injury n-codes |
| 87 | Household air pollution from solid fuels | 380 | Particulate matter pollution | 4 | 10 | 1 | Risk |
| 622 | Citrobacter spp. | 171 | Etiologies | 1 | 10 | 2 | Etiology |
| 211 | Mild hearing loss | 195 | Hearing loss | 2 | 10 | 3 | Impairment |
| 299 | Burns | 362 | Injuries | 1 | 10 | 4 | Injury n-codes |
| 88 | Ambient ozone pollution | 85 | Air pollution | 3 | 11 | 1 | Risk |
| 183 | Clostridium difficile | 171 | Etiologies | 1 | 11 | 2 | Etiology |
| 212 | Moderate hearing loss | 195 | Hearing loss | 2 | 11 | 3 | Impairment |
| 251 | Lower airway burns | 299 | Burns | 2 | 11 | 4 | Injury n-codes |
| 404 | Nitrogen dioxide pollution | 85 | Air pollution | 3 | 12 | 1 | Risk |
| 632 | Coagulase negative staphylococci | 171 | Etiologies | 1 | 12 | 2 | Etiology |
| 213 | Moderately severe hearing loss | 195 | Hearing loss | 2 | 12 | 3 | Impairment |
| 296 | Burns, <20% total burned surface area without lower airway burns | 299 | Burns | 2 | 12 | 4 | Injury n-codes |
| 331 | Non-optimal temperature | 202 | Environmental/occupational risks | 2 | 13 | 1 | Risk |
| 180 | Cryptosporidium | 171 | Etiologies | 1 | 13 | 2 | Etiology |
| 214 | Severe hearing loss | 195 | Hearing loss | 2 | 13 | 3 | Impairment |
| 297 | Burns, >=20% total burned surface area or >= 10% burned surface area if head/neck or hands/wrist involved w/o lower airway burns | 299 | Burns | 2 | 13 | 4 | Injury n-codes |
| 337 | High temperature | 331 | Non-optimal temperature | 3 | 14 | 1 | Risk |
| 179 | Entamoeba | 171 | Etiologies | 1 | 14 | 2 | Etiology |
| 215 | Profound hearing loss | 195 | Hearing loss | 2 | 14 | 3 | Impairment |
| 300 | Fractures | 362 | Injuries | 1 | 14 | 4 | Injury n-codes |
| 338 | Low temperature | 331 | Non-optimal temperature | 3 | 15 | 1 | Risk |
| 416 | Enterobacter spp | 171 | Etiologies | 1 | 15 | 2 | Etiology |
| 216 | Complete hearing loss | 195 | Hearing loss | 2 | 15 | 3 | Impairment |
| 256 | Fracture of clavicle, scapula, or humerus | 300 | Fractures | 2 | 15 | 4 | Injury n-codes |
| 89 | Other environmental risks | 202 | Environmental/occupational risks | 2 | 16 | 1 | Risk |
| 176 | Enteropathogenic E coli | 171 | Etiologies | 1 | 16 | 2 | Etiology |
| 196 | Heart failure | 191 | Impairments | 1 | 16 | 3 | Impairment |
| 257 | Fracture of face bones | 300 | Fractures | 2 | 16 | 4 | Injury n-codes |
| 90 | Residential radon | 89 | Other environmental risks | 3 | 17 | 1 | Risk |
| 177 | Enterotoxigenic E coli | 171 | Etiologies | 1 | 17 | 2 | Etiology |
| 379 | Treated heart failure | 196 | Heart failure | 2 | 17 | 3 | Impairment |
| 258 | Fracture of foot bones except ankle | 300 | Fractures | 2 | 17 | 4 | Injury n-codes |
| 91 | Lead exposure | 89 | Other environmental risks | 3 | 18 | 1 | Risk |
| 417 | Escherichia coli | 171 | Etiologies | 1 | 18 | 2 | Etiology |
| 217 | Mild heart failure | 196 | Heart failure | 2 | 18 | 3 | Impairment |
| 259 | Fracture of hand (wrist and other distal part of hand) | 300 | Fractures | 2 | 18 | 4 | Injury n-codes |
| 126 | Occupational risks | 202 | Environmental/occupational risks | 2 | 19 | 1 | Risk |
| 630 | Group A Streptococcus | 171 | Etiologies | 1 | 19 | 2 | Etiology |
| 218 | Moderate heart failure | 196 | Heart failure | 2 | 19 | 3 | Impairment |
| 260 | Fracture of hip | 300 | Fractures | 2 | 19 | 4 | Injury n-codes |
| 127 | Occupational carcinogens | 126 | Occupational risks | 3 | 20 | 1 | Risk |
| 393 | Group B streptococcus | 171 | Etiologies | 1 | 20 | 2 | Etiology |
| 219 | Severe heart failure | 196 | Heart failure | 2 | 20 | 3 | Impairment |
| 262 | Fracture of patella, tibia or fibula, or ankle | 300 | Fractures | 2 | 20 | 4 | Injury n-codes |
| 150 | Occupational exposure to asbestos | 127 | Occupational carcinogens | 4 | 21 | 1 | Risk |
| 419 | Haemophilus influenzae | 171 | Etiologies | 1 | 21 | 2 | Etiology |
| 198 | Infertility | 191 | Impairments | 1 | 21 | 3 | Impairment |
| 263 | Fracture of pelvis | 300 | Fractures | 2 | 21 | 4 | Injury n-codes |
| 151 | Occupational exposure to arsenic | 127 | Occupational carcinogens | 4 | 22 | 1 | Risk |
| 187 | Influenza | 171 | Etiologies | 1 | 22 | 2 | Etiology |
| 220 | Primary infertility | 198 | Infertility | 2 | 22 | 3 | Impairment |
| 264 | Fracture of radius and/or ulna | 300 | Fractures | 2 | 22 | 4 | Injury n-codes |
| 152 | Occupational exposure to benzene | 127 | Occupational carcinogens | 4 | 23 | 1 | Risk |
| 420 | Klebsiella pneumoniae | 171 | Etiologies | 1 | 23 | 2 | Etiology |
| 221 | Secondary infertility | 198 | Infertility | 2 | 23 | 3 | Impairment |
| 265 | Fracture of skull | 300 | Fractures | 2 | 23 | 4 | Injury n-codes |
| 153 | Occupational exposure to beryllium | 127 | Occupational carcinogens | 4 | 24 | 1 | Risk |
| 421 | Legionella spp | 171 | Etiologies | 1 | 24 | 2 | Etiology |
| 197 | Developmental intellectual disability | 191 | Impairments | 1 | 24 | 3 | Impairment |
| 266 | Fracture of sternum and/or fracture of one or more ribs | 300 | Fractures | 2 | 24 | 4 | Injury n-codes |
| 154 | Occupational exposure to cadmium | 127 | Occupational carcinogens | 4 | 25 | 1 | Risk |
| 422 | Listeria monocytogenes | 171 | Etiologies | 1 | 25 | 2 | Etiology |
| 236 | Borderline intellectual disability | 197 | Developmental intellectual disability | 2 | 25 | 3 | Impairment |
| 267 | Fracture of vertebral column | 300 | Fractures | 2 | 25 | 4 | Injury n-codes |
| 155 | Occupational exposure to chromium | 127 | Occupational carcinogens | 4 | 26 | 1 | Risk |
| 626 | Morganella spp. | 171 | Etiologies | 1 | 26 | 2 | Etiology |
| 222 | Mild intellectual disability | 197 | Developmental intellectual disability | 2 | 26 | 3 | Impairment |
| 268 | Fracture of femur, other than femoral neck | 300 | Fractures | 2 | 26 | 4 | Injury n-codes |
| 156 | Occupational exposure to diesel engine exhaust | 127 | Occupational carcinogens | 4 | 27 | 1 | Risk |
| 423 | Mycoplasma | 171 | Etiologies | 1 | 27 | 2 | Etiology |
| 223 | Moderate intellectual disability | 197 | Developmental intellectual disability | 2 | 27 | 3 | Impairment |
| 301 | Head Injuries | 362 | Injuries | 1 | 27 | 4 | Injury n-codes |
| 158 | Occupational exposure to formaldehyde | 127 | Occupational carcinogens | 4 | 28 | 1 | Risk |
| 431 | Neisseria meningitidis | 171 | Etiologies | 1 | 28 | 2 | Etiology |
| 224 | Severe intellectual disability | 197 | Developmental intellectual disability | 2 | 28 | 3 | Impairment |
| 269 | Minor TBI | 301 | Head Injuries | 2 | 28 | 4 | Injury n-codes |
| 159 | Occupational exposure to nickel | 127 | Occupational carcinogens | 4 | 29 | 1 | Risk |
| 633 | Non-polio enteroviruses | 171 | Etiologies | 1 | 29 | 2 | Etiology |
| 225 | Profound intellectual disability | 197 | Developmental intellectual disability | 2 | 29 | 3 | Impairment |
| 270 | Moderate/Severe TBI | 301 | Head Injuries | 2 | 29 | 4 | Injury n-codes |
| 160 | Occupational exposure to polycyclic aromatic hydrocarbons | 127 | Occupational carcinogens | 4 | 30 | 1 | Risk |
| 174 | Non-typhoidal Salmonella | 171 | Etiologies | 1 | 30 | 2 | Etiology |
| 199 | Pelvic inflammatory disease | 191 | Impairments | 1 | 30 | 3 | Impairment |
| 302 | Spinal Injuries | 362 | Injuries | 1 | 30 | 4 | Injury n-codes |
| 161 | Occupational exposure to silica | 127 | Occupational carcinogens | 4 | 31 | 1 | Risk |
| 184 | Norovirus | 171 | Etiologies | 1 | 31 | 2 | Etiology |
| 226 | Moderate pelvic inflammatory disease | 199 | Pelvic inflammatory disease | 2 | 31 | 3 | Impairment |
| 276 | Spinal cord lesion at neck level | 302 | Spinal Injuries | 2 | 31 | 4 | Injury n-codes |
| 162 | Occupational exposure to sulfuric acid | 127 | Occupational carcinogens | 4 | 32 | 1 | Risk |
| 621 | Other Acinetobacter species | 171 | Etiologies | 1 | 32 | 2 | Etiology |
| 227 | Severe pelvic inflammatory disease | 199 | Pelvic inflammatory disease | 2 | 32 | 3 | Impairment |
| 277 | Spinal cord lesion below neck level | 302 | Spinal Injuries | 2 | 32 | 4 | Injury n-codes |
| 237 | Occupational exposure to trichloroethylene | 127 | Occupational carcinogens | 4 | 33 | 1 | Risk |
| 424 | Other bacterial and viral pathogens | 171 | Etiologies | 1 | 33 | 2 | Etiology |
| 200 | Blindness and vision loss | 191 | Impairments | 1 | 33 | 3 | Impairment |
| 303 | Minor Injuries | 362 | Injuries | 1 | 33 | 4 | Injury n-codes |
| 128 | Occupational asthmagens | 126 | Occupational risks | 3 | 34 | 1 | Risk |
| 623 | Other fungi | 171 | Etiologies | 1 | 34 | 2 | Etiology |
| 233 | Presbyopia | 200 | Blindness and vision loss | 2 | 34 | 3 | Impairment |
| 255 | Muscle and tendon injuries, including sprains and strains lesser dislocations | 303 | Minor Injuries | 2 | 34 | 4 | Injury n-codes |
| 129 | Occupational particulate matter, gases, and fumes | 126 | Occupational risks | 3 | 35 | 1 | Risk |
| 624 | Other gram-negative bacteria | 171 | Etiologies | 1 | 35 | 2 | Etiology |
| 229 | Moderate vision loss | 200 | Blindness and vision loss | 2 | 35 | 3 | Impairment |
| 273 | Foreign body in ear | 303 | Minor Injuries | 2 | 35 | 4 | Injury n-codes |
| 130 | Occupational noise | 126 | Occupational risks | 3 | 36 | 1 | Risk |
| 625 | Other Klebsiella species | 171 | Etiologies | 1 | 36 | 2 | Etiology |
| 230 | Severe vision loss | 200 | Blindness and vision loss | 2 | 36 | 3 | Impairment |
| 284 | Open wound(s) | 303 | Minor Injuries | 2 | 36 | 4 | Injury n-codes |
| 131 | Occupational injuries | 126 | Occupational risks | 3 | 37 | 1 | Risk |
| 627 | Other Mycobacterium species (non-TB, non-Leprosy) | 171 | Etiologies | 1 | 37 | 2 | Etiology |
| 231 | Blindness | 200 | Blindness and vision loss | 2 | 37 | 3 | Impairment |
| 288 | Contusion in any part of the body | 303 | Minor Injuries | 2 | 37 | 4 | Injury n-codes |
| 132 | Occupational ergonomic factors | 126 | Occupational risks | 3 | 38 | 1 | Risk |
| 634 | Other streptococcus species | 171 | Etiologies | 1 | 38 | 2 | Etiology |
| 291 | Superficial injury of any part of the body | 303 | Minor Injuries | 2 | 38 | 4 | Injury n-codes |
| 203 | Behavioral risks | 169 | All risk factors | 1 | 39 | 1 | Risk |
| 628 | Proteus spp. | 171 | Etiologies | 1 | 39 | 2 | Etiology |
| 304 | Other Injuries | 362 | Injuries | 1 | 39 | 4 | Injury n-codes |
| 92 | Child and maternal malnutrition | 203 | Behavioral risks | 2 | 40 | 1 | Risk |
| 427 | Pseudomonas aeruginosa | 171 | Etiologies | 1 | 40 | 2 | Etiology |
| 252 | Dislocation of hip | 304 | Other Injuries | 2 | 40 | 4 | Injury n-codes |
| 93 | Suboptimal breastfeeding | 92 | Child and maternal malnutrition | 3 | 41 | 1 | Risk |
| 190 | Respiratory syncytial virus | 171 | Etiologies | 1 | 41 | 2 | Etiology |
| 253 | Dislocation of knee | 304 | Other Injuries | 2 | 41 | 4 | Injury n-codes |
| 136 | Non-exclusive breastfeeding | 93 | Suboptimal breastfeeding | 4 | 42 | 1 | Risk |
| 181 | Rotavirus | 171 | Etiologies | 1 | 42 | 2 | Etiology |
| 254 | Dislocation of shoulder | 304 | Other Injuries | 2 | 42 | 4 | Injury n-codes |
| 137 | Discontinued breastfeeding | 93 | Suboptimal breastfeeding | 4 | 43 | 1 | Risk |
| 629 | Serratia spp. | 171 | Etiologies | 1 | 43 | 2 | Etiology |
| 274 | Foreign body in respiratory system | 304 | Other Injuries | 2 | 43 | 4 | Injury n-codes |
| 239 | Child growth failure | 92 | Child and maternal malnutrition | 3 | 44 | 1 | Risk |
| 175 | Shigella | 171 | Etiologies | 1 | 44 | 2 | Etiology |
| 275 | Foreign body in GI and urogenital system | 304 | Other Injuries | 2 | 44 | 4 | Injury n-codes |
| 94 | Child underweight | 239 | Child growth failure | 4 | 45 | 1 | Risk |
| 428 | Staphylococcus aureus | 171 | Etiologies | 1 | 45 | 2 | Etiology |
| 278 | Drowning and nonfatal submersion | 304 | Other Injuries | 2 | 45 | 4 | Injury n-codes |
| 240 | Child wasting | 239 | Child growth failure | 4 | 46 | 1 | Risk |
| 430 | Streptococcus pneumoniae | 171 | Etiologies | 1 | 46 | 2 | Etiology |
| 279 | Asphyxiation | 304 | Other Injuries | 2 | 46 | 4 | Injury n-codes |
| 241 | Child stunting | 239 | Child growth failure | 4 | 47 | 1 | Risk |
| 917 | Other viral etiologies of meningitis | 171 | Etiologies | 1 | 47 | 2 | Etiology |
| 280 | Crush injury | 304 | Other Injuries | 2 | 47 | 4 | Injury n-codes |
| 339 | Low birth weight and short gestation | 92 | Child and maternal malnutrition | 3 | 48 | 1 | Risk |
| 281 | Nerve injury | 304 | Other Injuries | 2 | 48 | 4 | Injury n-codes |
| 334 | Short gestation | 339 | Low birth weight and short gestation | 4 | 49 | 1 | Risk |
| 282 | Injury to eyes | 304 | Other Injuries | 2 | 49 | 4 | Injury n-codes |
| 335 | Low birth weight | 339 | Low birth weight and short gestation | 4 | 50 | 1 | Risk |
| 285 | Poisoning requiring urgent care | 304 | Other Injuries | 2 | 50 | 4 | Injury n-codes |
| 95 | Iron deficiency | 92 | Child and maternal malnutrition | 3 | 51 | 1 | Risk |
| 286 | Severe chest Injury | 304 | Other Injuries | 2 | 51 | 4 | Injury n-codes |
| 96 | Vitamin A deficiency | 92 | Child and maternal malnutrition | 3 | 52 | 1 | Risk |
| 287 | Internal hemorrhage in abdomen and pelvis | 304 | Other Injuries | 2 | 52 | 4 | Injury n-codes |
| 97 | Zinc deficiency | 92 | Child and maternal malnutrition | 3 | 53 | 1 | Risk |
| 289 | Effect of different environmental factors | 304 | Other Injuries | 2 | 53 | 4 | Injury n-codes |
| 98 | Tobacco | 203 | Behavioral risks | 2 | 54 | 1 | Risk |
| 290 | Complications following therapeutic procedures | 304 | Other Injuries | 2 | 54 | 4 | Injury n-codes |
| 99 | Smoking | 98 | Tobacco | 3 | 55 | 1 | Risk |
| 292 | Multiple fractures, dislocations, crashes, wounds, pains, and strains | 304 | Other Injuries | 2 | 55 | 4 | Injury n-codes |
| 332 | Chewing tobacco | 98 | Tobacco | 3 | 56 | 1 | Risk |
| 100 | Secondhand smoke | 98 | Tobacco | 3 | 57 | 1 | Risk |
| 102 | High alcohol use | 203 | Behavioral risks | 2 | 58 | 1 | Risk |
| 103 | Drug use | 203 | Behavioral risks | 2 | 59 | 1 | Risk |
| 110 | Dietary risks | 203 | Behavioral risks | 2 | 60 | 1 | Risk |
| 111 | Diet low in fruits | 110 | Dietary risks | 3 | 61 | 1 | Risk |
| 112 | Diet low in vegetables | 110 | Dietary risks | 3 | 62 | 1 | Risk |
| 333 | Diet low in legumes | 110 | Dietary risks | 3 | 63 | 1 | Risk |
| 113 | Diet low in whole grains | 110 | Dietary risks | 3 | 64 | 1 | Risk |
| 114 | Diet low in nuts and seeds | 110 | Dietary risks | 3 | 65 | 1 | Risk |
| 115 | Diet low in milk | 110 | Dietary risks | 3 | 66 | 1 | Risk |
| 116 | Diet high in red meat | 110 | Dietary risks | 3 | 67 | 1 | Risk |
| 117 | Diet high in processed meat | 110 | Dietary risks | 3 | 68 | 1 | Risk |
| 118 | Diet high in sugar-sweetened beverages | 110 | Dietary risks | 3 | 69 | 1 | Risk |
| 119 | Diet low in fiber | 110 | Dietary risks | 3 | 70 | 1 | Risk |
| 147 | Diet low in calcium | 110 | Dietary risks | 3 | 71 | 1 | Risk |
| 121 | Diet low in seafood omega-3 fatty acids | 110 | Dietary risks | 3 | 72 | 1 | Risk |
| 122 | Diet low in omega-6 polyunsaturated fatty acids | 110 | Dietary risks | 3 | 73 | 1 | Risk |
| 123 | Diet high in trans fatty acids | 110 | Dietary risks | 3 | 74 | 1 | Risk |
| 124 | Diet high in sodium | 110 | Dietary risks | 3 | 75 | 1 | Risk |
| 135 | Intimate partner violence | 203 | Behavioral risks | 2 | 76 | 1 | Risk |
| 381 | Sexual violence against children and bullying | 203 | Behavioral risks | 2 | 77 | 1 | Risk |
| 134 | Sexual violence against children | 381 | Sexual violence against children and bullying | 3 | 78 | 1 | Risk |
| 363 | Bullying victimization | 381 | Sexual violence against children and bullying | 3 | 79 | 1 | Risk |
| 170 | Unsafe sex | 203 | Behavioral risks | 2 | 80 | 1 | Risk |
| 125 | Low physical activity | 203 | Behavioral risks | 2 | 81 | 1 | Risk |
| 104 | Metabolic risks | 169 | All risk factors | 1 | 82 | 1 | Risk |
| 105 | High fasting plasma glucose | 104 | Metabolic risks | 2 | 83 | 1 | Risk |
| 367 | High LDL cholesterol | 104 | Metabolic risks | 2 | 84 | 1 | Risk |
| 107 | High systolic blood pressure | 104 | Metabolic risks | 2 | 85 | 1 | Risk |
| 108 | High body-mass index | 104 | Metabolic risks | 2 | 86 | 1 | Risk |
| 109 | Low bone mineral density | 104 | Metabolic risks | 2 | 87 | 1 | Risk |
| 341 | Kidney dysfunction | 104 | Metabolic risks | 2 | 88 | 1 | Risk |
