# Supplementary material for: Burden of chronic kidney disease and its attributable risk factors in 204 countries and territories, 1990–2021: Results from the Global Burden of Disease study 2021
Source: Medicine (Baltimore). 2026 Aug 7;105(32):e50111. doi: 10.1097/MD.0000000000050111 (PMC13456769; doi:10.1097/MD.0000000000050111)
Supplement: Supplementary file 3 [file medi-105-e50111-s005.docx]

**Table S3.** Prevalent cases of chronic kidney disease in 1990 and 2021 and the percentage change in the age-standardised rates (ASRs) per 100,000, by location.

|  | **1990** | | **2021** | | **Percentage change in the ASRs per 100,000** |
| --- | --- | --- | --- | --- | --- |
|  | **No (95% UI)** | **ASRs per 100,000 (95% UI)** | **No (95% UI)** | **ASRs per 100,000 (95% UI)** |  |
| **Global** | 350962674 (326973785,376155723) | 8072.8 (7560.4,8634.1) | 673722703 (629095119,722364096) | 8006 (7482.1,8575.6) | -0.8 (-1.9,0.1) |
| **Andean Latin America** | 1465459 (1365798,1575973) | 5766.6 (5377.8,6181) | 3743589 (3478926,4028408) | 5946.1 (5524.8,6371.5) | 3.1 (0.5,5.2) |
| Bolivia (Plurinational State of) | 242900 (225810,260389) | 6093.3 (5681.1,6496.9) | 628320 (584306,676494) | 6177.1 (5740.2,6632.4) | 1.4 (-1.2,4) |
| Ecuador | 392453 (366304,421830) | 5893.4 (5492,6356.8) | 1073132 (996177,1149397) | 6249.3 (5811.7,6689.2) | 6 (2.2,9.9) |
| Peru | 830105 (773577,894482) | 5621.6 (5237.5,6016) | 2042137 (1897519,2195297) | 5732.7 (5325.1,6154.3) | 2 (-1.3,4.6) |
| **Australasia** | 1399739 (1315064,1481429) | 6084.4 (5719.4,6456.3) | 2810777 (2637789,2995329) | 5910 (5548.2,6301.6) | -2.9 (-5.7,0.1) |
| Australia | 1165447 (1094777,1238029) | 6076.8 (5714.8,6452.5) | 2353593 (2211156,2523412) | 5877.9 (5525.9,6272.1) | -3.3 (-6.5,0.4) |
| New Zealand | 234292 (217327,250752) | 6128.4 (5708.2,6546.6) | 457184 (423466,488353) | 6072.6 (5649.2,6499) | -0.9 (-3.3,1.6) |
| **Caribbean** | 1867236 (1741663,2000227) | 6480.2 (6037.6,6925.8) | 3468705 (3235452,3720720) | 6633 (6181.3,7112.4) | 2.4 (0.7,4) |
| Antigua and Barbuda | 3729 (3474,3994) | 6700.1 (6253.5,7179.1) | 7270 (6762,7815) | 6956.3 (6479.9,7455.7) | 3.8 (0.8,6.7) |
| Bahamas | 12715 (11806,13734) | 6542.7 (6098.9,7002.9) | 28177 (26096,30221) | 6759.3 (6258.6,7239.1) | 3.3 (0.7,6.1) |
| Barbados | 17997 (16818,19169) | 6459.9 (6051.7,6909.2) | 28988 (27004,31010) | 6676.8 (6215.3,7161.7) | 3.4 (0.7,6) |
| Belize | 7609 (7071,8133) | 6629.9 (6188.8,7130.9) | 24923 (23248,26780) | 6996.9 (6519.9,7476.1) | 5.5 (1.7,9.4) |
| Bermuda | 4041 (3755,4358) | 6209.7 (5792.5,6656.9) | 6571 (6087,7048) | 6114.2 (5679.3,6583.5) | -1.5 (-4.2,1.2) |
| Cuba | 670575 (622916,717810) | 6258.5 (5824.6,6706.2) | 1023168 (949443,1092062) | 6157.3 (5728.3,6614.3) | -1.6 (-4.8,1.5) |
| Dominica | 4263 (3961,4572) | 6991.4 (6509.2,7479.9) | 5745 (5329,6134) | 7303.9 (6813,7818.6) | 4.5 (1.7,7.2) |
| Dominican Republic | 299732 (277339,324408) | 6357.9 (5927.6,6822.6) | 710332 (660993,766986) | 6688.8 (6211.1,7193.2) | 5.2 (2.4,8.3) |
| Grenada | 5042 (4684,5387) | 6983.1 (6481.8,7464) | 8182 (7614,8784) | 7279.5 (6800.4,7818.7) | 4.2 (1.5,7.1) |
| Guyana | 34192 (31637,36857) | 6803.9 (6325.9,7280.5) | 48899 (45521,52822) | 7062.5 (6579,7608.2) | 3.8 (0.6,7) |
| Haiti | 274957 (254724,296317) | 6959.2 (6473,7441.6) | 662956 (613153,717829) | 7123.7 (6614.8,7647.9) | 2.4 (0.1,4.4) |
| Jamaica | 122884 (115047,131333) | 6456.9 (6045.3,6921.3) | 209554 (194578,224704) | 6708.1 (6219,7188.8) | 3.9 (0.8,7) |
| Puerto Rico | 241664 (224964,259466) | 6681.7 (6222.4,7166.2) | 380144 (353031,405700) | 6833 (6356.4,7333.3) | 2.3 (-0.9,5.2) |
| Saint Kitts and Nevis | 2582 (2409,2774) | 7034.2 (6570.2,7551.8) | 4826 (4470,5192) | 6962.4 (6470,7455.7) | -1 (-3.8,1.5) |
| Saint Lucia | 6820 (6312,7305) | 6901.6 (6410.1,7371.5) | 15679 (14634,16864) | 6954.1 (6489.8,7468.2) | 0.8 (-2,3.3) |
| Saint Vincent and the Grenadines | 5218 (4816,5601) | 6544.4 (6043.5,7001.3) | 9229 (8590,9910) | 6902.6 (6435.8,7413.5) | 5.5 (2.5,8.4) |
| Suriname | 19697 (18356,21176) | 6671.6 (6215.3,7151.6) | 42747 (39950,45625) | 6878.3 (6435.8,7347.1) | 3.1 (0,6.1) |
| Trinidad and Tobago | 64179 (59428,68941) | 6674.1 (6204.2,7119.3) | 124244 (115206,133282) | 7009.5 (6525,7524.9) | 5 (1.8,8.2) |
| United States Virgin Islands | 6113 (5650,6602) | 6554.3 (6091.8,7043.7) | 9684 (8946,10346) | 6791.6 (6302.6,7286) | 3.6 (1.2,6.2) |
| **Central Asia** | 5534240 (5182702,5905060) | 10649.1 (10005.7,11309.3) | 9356434 (8704146,9969948) | 10698.2 (10022.9,11348.1) | 0.5 (-1.3,2.1) |
| Armenia | 300635 (278865,323273) | 10265.9 (9580.8,10974) | 404070 (378068,430935) | 10245 (9598,10928) | -0.2 (-4,3.9) |
| Azerbaijan | 593465 (554684,636804) | 10594.8 (9927.5,11288.4) | 1146469 (1073053,1221894) | 10696 (10019,11338.2) | 1 (-2,4.4) |
| Georgia | 632971 (590879,679007) | 10617.5 (9919.4,11342.7) | 544204 (506648,579897) | 10551.1 (9851.3,11224) | -0.6 (-4.5,3.5) |
| Kazakhstan | 1471284 (1368347,1573180) | 10730.4 (10056.3,11412) | 1952569 (1812225,2090896) | 10662.4 (9928.1,11394.4) | -0.6 (-3.2,1.9) |
| Kyrgyzstan | 349912 (326466,372023) | 10650.2 (9986.3,11306.1) | 554675 (518240,592588) | 10147.7 (9422.6,10780.3) | -4.7 (-7.7,-1.7) |
| Mongolia | 146040 (136801,156132) | 11239.6 (10569.3,11958.8) | 287978 (267255,309250) | 10667.7 (9937.1,11381.2) | -5.1 (-8.1,-1.9) |
| Tajikistan | 322282 (300325,346451) | 9924.7 (9264.6,10630.8) | 708588 (659872,761599) | 9851.2 (9246.7,10479) | -0.7 (-3.6,2.4) |
| Turkmenistan | 254957 (238341,273926) | 11011.1 (10342.1,11708.9) | 484074 (452902,515793) | 10909.7 (10221.7,11599.9) | -0.9 (-4.6,2.9) |
| Uzbekistan | 1462693 (1370470,1565221) | 10757.8 (10084.1,11467.1) | 3273806 (3046385,3512972) | 11069.1 (10338.8,11793.6) | 2.9 (-0.1,6.1) |
| **Central Europe** | 8932587 (8363724,9525233) | 6368.6 (5978,6772) | 11481266 (10766159,12145281) | 6205.2 (5841,6596.2) | -2.6 (-4.1,-1) |
| Albania | 152351 (142462,163786) | 6243.7 (5830.9,6667.4) | 230609 (214471,246180) | 6154 (5711.8,6544.9) | -1.4 (-4.3,1.4) |
| Bosnia and Herzegovina | 260939 (242972,279575) | 6132.4 (5735.3,6544.3) | 323305 (300335,346499) | 6233.5 (5827.2,6665.1) | 1.6 (-1.3,4.6) |
| Bulgaria | 673785 (626288,720194) | 6187.8 (5773,6611.1) | 726001 (674875,778060) | 6282.1 (5866.9,6704.4) | 1.5 (-3.2,6.1) |
| Croatia | 352794 (326765,378976) | 6188.9 (5755.5,6629) | 441010 (407740,471831) | 6109.1 (5670,6576.3) | -1.3 (-4.8,2.3) |
| Czechia | 777458 (723745,829773) | 6159.6 (5756.9,6580.7) | 1033665 (960274,1099534) | 5875 (5479.3,6268.4) | -4.6 (-7.4,-1.4) |
| Hungary | 816867 (760112,874417) | 6193.2 (5788.2,6630.4) | 965275 (898080,1028450) | 5992.9 (5610.7,6393.6) | -3.2 (-6.3,-0.5) |
| Montenegro | 40300 (37650,42973) | 6416 (6010.8,6835.5) | 54152 (50266,57833) | 6415 (5962.1,6863.8) | 0 (-2.6,2.6) |
| North Macedonia | 140331 (130960,149494) | 7386 (6902,7876.8) | 220103 (205048,234493) | 7377.4 (6929.9,7832.2) | -0.1 (-3.1,3.2) |
| Poland | 2866015 (2680925,3059816) | 6850.9 (6418.5,7310.9) | 3872502 (3620089,4134821) | 6415.3 (6012.6,6855.3) | -6.4 (-7.8,-4.8) |
| Romania | 1605525 (1495525,1715394) | 6180.4 (5775.4,6591.1) | 1993675 (1890808,2098153) | 6300.7 (5964.6,6652.5) | 1.9 (-2.1,5.8) |
| Serbia | 603591 (559791,648669) | 5667.2 (5283.7,6069) | 753941 (699986,811166) | 5593.3 (5189.2,6041) | -1.3 (-4.2,1.6) |
| Slovakia | 357483 (334696,382908) | 6221.6 (5843.7,6645) | 486371 (454761,518472) | 5960 (5575.9,6352.7) | -4.2 (-7.1,-1.3) |
| Slovenia | 142299 (133101,152114) | 6083.6 (5696.5,6507.4) | 213545 (199557,227477) | 5968.2 (5539.4,6369.4) | -1.9 (-5.4,1.8) |
| **Central Latin America** | 8628113 (8065183,9238847) | 8477 (7931,9062.8) | 22101801 (20701763,23422153) | 8642.9 (8089.3,9163.7) | 2 (0.1,3.9) |
| Colombia | 1697055 (1583361,1826064) | 7912.4 (7376.2,8461.8) | 4222423 (3939788,4478779) | 7695.6 (7194,8158.8) | -2.7 (-6.5,1.3) |
| Costa Rica | 197345 (183956,211905) | 9636.7 (8996.2,10298.8) | 517788 (484174,550601) | 9532.1 (8909.8,10127.1) | -1.1 (-5.4,4.4) |
| El Salvador | 239034 (222018,257099) | 6892.2 (6378.2,7415.7) | 460009 (431238,489587) | 7296.1 (6841.6,7771) | 5.9 (1.3,10.5) |
| Guatemala | 342544 (318619,365583) | 8065.5 (7574.1,8638.4) | 1096863 (1023152,1176256) | 8857.7 (8275.1,9478.3) | 9.8 (6,13.6) |
| Honduras | 209413 (194859,225041) | 8405.3 (7844.1,8968.8) | 633449 (591340,678996) | 8613 (8025.9,9216) | 2.5 (-0.9,6) |
| Mexico | 4594717 (4287702,4924515) | 8781.5 (8206.6,9393.8) | 11780174 (11009027,12569139) | 9052.2 (8453.8,9672.7) | 3.1 (1.5,4.6) |
| Nicaragua | 190029 (177519,203548) | 9675.2 (9075.4,10313.2) | 575347 (537918,614144) | 10511.2 (9794.3,11157.8) | 8.6 (3.6,14.9) |
| Panama | 142393 (132809,153036) | 8261.6 (7709.3,8864.8) | 373196 (347346,398963) | 8453.5 (7857.2,9031.7) | 2.3 (-2.4,6.4) |
| Venezuela (Bolivarian Republic of) | 1015584 (943619,1092889) | 8453 (7868.5,9056.9) | 2442552 (2275365,2599385) | 8317.3 (7756.4,8846.8) | -1.6 (-6,3.5) |
| **Central Sub-Saharan Africa** | 2633228 (2443845,2831336) | 9222 (8598.7,9806.7) | 6811951 (6363747,7313187) | 9165.2 (8640.8,9709.7) | -0.6 (-2.5,1.6) |
| Angola | 473841 (440001,510739) | 9090.8 (8510.8,9718.4) | 1482282 (1374318,1605870) | 9018.9 (8383.2,9636) | -0.8 (-3.2,1.7) |
| Central African Republic | 133406 (123251,143340) | 9207.4 (8552.5,9860.3) | 276101 (255353,297770) | 9141.2 (8513.3,9769.2) | -0.7 (-3.3,1.8) |
| Congo | 122987 (114132,132085) | 9370.1 (8763.1,9998) | 331895 (307907,357776) | 9319.6 (8696.9,9931.8) | -0.5 (-3.1,1.7) |
| Democratic Republic of the Congo | 1824071 (1691866,1955647) | 9255.3 (8613.5,9831.1) | 4529099 (4238150,4861233) | 9196.8 (8707.1,9726.6) | -0.6 (-3.3,2.3) |
| Equatorial Guinea | 20935 (19447,22604) | 9087.3 (8498.5,9734.5) | 75546 (69523,82103) | 9342.3 (8719.9,9945.4) | 2.8 (0.5,5.3) |
| Gabon | 57987 (53943,62001) | 9126 (8487.1,9779.8) | 117028 (108707,125772) | 9238 (8645.4,9881) | 1.2 (-1.6,3.7) |
| **East Asia** | 69797869 (64858255,75190145) | 7083.6 (6610.1,7593.5) | 122849232 (113607491,132118615) | 6258.1 (5823.1,6729.8) | -11.7 (-13.5,-10.2) |
| China | 67398525 (62608781,72614585) | 7091.9 (6614.3,7603.1) | 118403911 (109388671,127484286) | 6249.4 (5812.4,6720.2) | -11.9 (-13.7,-10.4) |
| Democratic People's Republic of Korea | 1150078 (1067243,1233674) | 6571.8 (6117.4,7043.5) | 2027239 (1883443,2173423) | 6390.8 (5955.9,6816.8) | -2.8 (-5.4,0) |
| Taiwan (Province of China) | 1249266 (1163816,1344723) | 7155.4 (6728.1,7647.1) | 2418082 (2270339,2579826) | 6571.2 (6152.2,7040.8) | -8.2 (-11,-5.4) |
| **Eastern Europe** | 24173788 (22444048,26062971) | 9327.5 (8680,10050.1) | 27312843 (25403078,29335270) | 9266.3 (8619.4,9989.5) | -0.7 (-1.5,0.2) |
| Belarus | 1162192 (1079543,1248012) | 9654.1 (8993.2,10364.9) | 1314740 (1220742,1405904) | 9704.3 (9000.4,10430.5) | 0.5 (-2.8,3.2) |
| Estonia | 181645 (168130,196037) | 9678.7 (8968.1,10435.8) | 206581 (192387,221597) | 9660.9 (9006.3,10389.7) | -0.2 (-2.3,2) |
| Latvia | 313358 (290679,336853) | 9660.9 (8967.6,10391.3) | 303626 (282328,323377) | 9785.8 (9110.5,10514.3) | 1.3 (-1.1,3.8) |
| Lithuania | 414295 (384463,446122) | 9790.2 (9086.7,10563.5) | 441211 (411206,472545) | 9837.2 (9134.9,10590) | 0.5 (-3,3.9) |
| Republic of Moldova | 469578 (435381,501649) | 10729.3 (10014.3,11456.7) | 596118 (553806,636138) | 11293.7 (10519.6,12048.1) | 5.3 (1.9,8.5) |
| Russian Federation | 15387961 (14274967,16655381) | 9062.2 (8429.2,9771.4) | 18056081 (16744178,19455656) | 8973.8 (8345.5,9704.8) | -1 (-1.7,-0.2) |
| Ukraine | 6244758 (5793184,6760948) | 9772.5 (9071.5,10538.3) | 6394485 (5969352,6867844) | 9814.1 (9113.9,10548.5) | 0.4 (-2.1,2.8) |
| **Eastern Sub-Saharan Africa** | 5981606 (5488507,6503182) | 5677.2 (5266.2,6113.1) | 15017846 (13741759,16422384) | 5821.3 (5404.5,6293.6) | 2.5 (1.7,3.3) |
| Burundi | 187278 (170970,204259) | 5868.1 (5412.1,6352.8) | 456223 (414259,502680) | 5921.6 (5472.5,6436.6) | 0.9 (-1.4,3.5) |
| Comoros | 15628 (14361,16936) | 5870.7 (5455.7,6305.4) | 35339 (32389,38509) | 5851.8 (5407.4,6323.6) | -0.3 (-2.6,2.3) |
| Djibouti | 13012 (11876,14272) | 5545.1 (5142.1,5961.8) | 55546 (50733,60942) | 5776.9 (5342.9,6232.5) | 4.2 (1.8,6.5) |
| Eritrea | 99470 (90769,109284) | 5529.9 (5117.9,5982.1) | 244600 (224167,268056) | 5676.6 (5255.1,6117.6) | 2.7 (0.2,4.8) |
| Ethiopia | 1542523 (1422919,1673239) | 5569.3 (5168.8,5985.1) | 3762262 (3443544,4113603) | 5649.3 (5230.3,6098.2) | 1.4 (-0.1,3) |
| Kenya | 700742 (642264,764896) | 5761.7 (5343.2,6221.9) | 1979251 (1807371,2167364) | 5900.7 (5470,6369) | 2.4 (1.8,3) |
| Madagascar | 389060 (359027,423079) | 5625.8 (5196.6,6061.5) | 994473 (908917,1089154) | 5685.3 (5246.2,6164.1) | 1.1 (-1.3,3.5) |
| Malawi | 326018 (298440,355273) | 5918.5 (5492,6409.4) | 705439 (644778,770229) | 6124.8 (5658.2,6619.3) | 3.5 (1,5.8) |
| Mozambique | 471644 (433799,513878) | 5918.7 (5479.6,6395.1) | 1066862 (978804,1170332) | 6162.6 (5705.7,6708.9) | 4.1 (1.8,6.6) |
| Rwanda | 234499 (214398,255853) | 5852.6 (5422.1,6320) | 513932 (471486,561218) | 5696.5 (5277.2,6164.1) | -2.7 (-5,-0.2) |
| Somalia | 224333 (205558,246035) | 5516.9 (5100.6,5954.8) | 603690 (549230,663805) | 5565 (5152.6,6029.6) | 0.9 (-1.3,3.1) |
| South Sudan | 193641 (177480,211370) | 5618.8 (5175.1,6089.5) | 323627 (296973,354259) | 5730.3 (5317.9,6182.2) | 2 (-0.4,4.3) |
| Uganda | 554191 (507911,603268) | 6119.9 (5653.8,6571.6) | 1414668 (1296989,1547642) | 6081.4 (5638.5,6582.4) | -0.6 (-2.9,1.6) |
| United Republic of Tanzania | 793891 (729869,860415) | 5368.9 (4960.7,5794.8) | 2186544 (2008467,2390011) | 5861.5 (5421.9,6348.6) | 9.2 (6.6,11.7) |
| Zambia | 231401 (212298,251949) | 5513.6 (5099.7,5947.9) | 662316 (603070,727722) | 5769.9 (5330.9,6256.6) | 4.6 (2.2,7.2) |
| **High-income Asia Pacific** | 16878162 (15709633,18083496) | 8566.1 (7979.5,9169.5) | 29276226 (27336162,31020670) | 7920.7 (7393.7,8457.4) | -7.5 (-8.5,-6.6) |
| Brunei Darussalam | 15220 (13998,16528) | 9497.7 (8852.2,10130.5) | 39451 (36520,42780) | 9461.9 (8796.6,10168.9) | -0.4 (-3.3,2.4) |
| Japan | 13889911 (12903717,14885294) | 8744.8 (8150.4,9364.1) | 22877631 (21341971,24294034) | 8264.3 (7717.7,8855.7) | -5.5 (-6.2,-4.7) |
| Republic of Korea | 2703535 (2505415,2935211) | 7789.4 (7280,8381.1) | 5620395 (5254764,6008210) | 6872.2 (6417,7364.4) | -11.8 (-15.2,-8.6) |
| Singapore | 269496 (247876,292201) | 9889.3 (9177.1,10490.2) | 738749 (680520,792459) | 9230 (8547.7,9877.1) | -6.7 (-9.9,-3.5) |
| **High-income North America** | 25125238 (23432071,26893288) | 7401.1 (6920.4,7900.3) | 42488578 (39404535,45165702) | 7434.7 (6959.3,7911.4) | 0.5 (-0.6,1.8) |
| Canada | 2229477 (2055472,2415616) | 7026.4 (6480.6,7603.7) | 3809329 (3540568,4060952) | 6524.4 (6091.5,7000.7) | -7.1 (-13.5,-2.3) |
| Greenland | 2958 (2729,3212) | 6970.7 (6515.6,7414.6) | 4412 (4094,4732) | 6988.2 (6509.1,7491.4) | 0.3 (-2.6,3.1) |
| United States of America | 22892228 (21306718,24521743) | 7438.3 (6947,7942.8) | 38674171 (35778672,41193631) | 7550.8 (7050.6,8067.4) | 1.5 (0.3,2.7) |
| **North Africa and Middle East** | 19739135 (18244354,21279746) | 9119.7 (8490.9,9769.6) | 49668756 (45835433,53616786) | 9180 (8523.5,9830.1) | 0.7 (-0.3,1.8) |
| Afghanistan | 611982 (569081,656341) | 8808.9 (8175.3,9452.3) | 1440765 (1317910,1571513) | 8934.9 (8262.8,9627.3) | 1.4 (-0.7,3.5) |
| Algeria | 1413887 (1302734,1527151) | 8946 (8267.2,9615.9) | 3553584 (3275804,3852065) | 8807.2 (8150,9515.2) | -1.6 (-3.7,0.9) |
| Bahrain | 31525 (28728,34958) | 9152.1 (8487.5,9866.9) | 130181 (119335,142831) | 9108.5 (8445.2,9765.8) | -0.5 (-2.7,1.9) |
| Egypt | 3069526 (2834187,3319651) | 8613.6 (8031.7,9206) | 7320032 (6762507,7887829) | 9167.8 (8515,9825.4) | 6.4 (4.3,8.7) |
| Iran (Islamic Republic of) | 3141831 (2920460,3371296) | 9513.7 (8874,10180.4) | 8145077 (7562399,8737333) | 9474.8 (8837.1,10118.4) | -0.4 (-1.5,0.6) |
| Iraq | 1026557 (949992,1108210) | 9536.9 (8851.5,10239.3) | 2977727 (2740400,3234895) | 9464.2 (8764.4,10188.2) | -0.8 (-3.1,1.6) |
| Jordan | 187654 (172497,203471) | 9249.5 (8606.8,9945.6) | 889238 (817131,968041) | 8989.7 (8318.6,9729.3) | -2.8 (-5.4,-0.1) |
| Kuwait | 106214 (96298,117759) | 8982.8 (8321.1,9651.6) | 393166 (356902,436552) | 8593.1 (7931.8,9269.4) | -4.3 (-6.9,-1.4) |
| Lebanon | 211783 (195737,228352) | 8827.6 (8183.8,9494.6) | 548008 (508010,589947) | 8988.2 (8309.1,9607.8) | 1.8 (-0.4,4.2) |
| Libya | 228746 (211473,246316) | 9024.4 (8360,9727.3) | 610601 (560616,667943) | 9269.1 (8567.5,10032.2) | 2.7 (0.4,5) |
| Morocco | 1521333 (1399644,1649317) | 8496.6 (7864,9155.1) | 3125307 (2880319,3372664) | 8498.4 (7864,9135) | 0 (-2.2,2.3) |
| Oman | 98358 (89680,108123) | 8427.7 (7838.2,9068.7) | 344284 (313617,379720) | 9024.5 (8385.7,9702.4) | 7.1 (4.6,9.6) |
| Palestine | 105072 (97202,113180) | 9224.6 (8569.8,9850.2) | 316240 (292025,342754) | 8942.6 (8314.5,9603.5) | -3.1 (-5.4,-0.4) |
| Qatar | 28926 (26054,32385) | 9257.3 (8540.3,9961.5) | 238389 (214245,267350) | 9111.2 (8448,9852.6) | -1.6 (-4.4,1.4) |
| Saudi Arabia | 854299 (786274,926817) | 9213.2 (8560.7,9892.3) | 3067523 (2798321,3367524) | 9245.5 (8568.2,9935.6) | 0.4 (-2.2,2.8) |
| Sudan | 1061226 (976894,1142517) | 8869.2 (8238.7,9502) | 2607644 (2388823,2834157) | 9227.5 (8545.5,9928.6) | 4 (1.6,6.4) |
| Syrian Arab Republic | 651505 (602699,705033) | 9137.9 (8502.5,9774.2) | 1213830 (1120670,1309607) | 8947.4 (8264.7,9632.4) | -2.1 (-5,0.7) |
| Tunisia | 522181 (483654,562934) | 8690.3 (8101.6,9339.4) | 1144106 (1055790,1229704) | 8690.2 (8056.9,9347.2) | 0 (-2.3,2.6) |
| Turkey | 4172206 (3844649,4504302) | 9813.7 (9115.6,10517.9) | 8863959 (8169195,9513046) | 9503.7 (8759.6,10189.4) | -3.2 (-5.6,-0.6) |
| United Arab Emirates | 123088 (111138,138046) | 9906.1 (9179.7,10668.9) | 940579 (846409,1053023) | 9512.7 (8796.6,10259.4) | -4 (-6,-1.6) |
| Yemen | 560437 (518019,609175) | 8318 (7725.8,8913.9) | 1752190 (1608715,1906736) | 8511.9 (7887.1,9137.9) | 2.3 (-0.1,4.5) |
| **Oceania** | 306814 (282467,332777) | 7742.4 (7231.4,8304.2) | 776661 (715870,846878) | 7950.7 (7408.8,8567.7) | 2.7 (0.7,4.4) |
| American Samoa | 2729 (2519,2958) | 8831.3 (8197.4,9501.6) | 4468 (4153,4802) | 9256.7 (8599,9943) | 4.8 (2.1,7.6) |
| Cook Islands | 1150 (1062,1242) | 8019.6 (7447.9,8644.5) | 1841 (1712,1970) | 8230 (7649.9,8830.4) | 2.6 (0.2,5.2) |
| Fiji | 43084 (39626,46792) | 8584.3 (7980.7,9231) | 74318 (68583,80409) | 8913.8 (8286.6,9600.9) | 3.8 (1.2,7) |
| Guam | 8188 (7532,8928) | 7935.1 (7378.4,8544.5) | 16160 (14994,17291) | 8480.2 (7890.5,9106) | 6.9 (4.2,9.6) |
| Kiribati | 4029 (3716,4354) | 8459.6 (7856.8,9093.5) | 7836 (7239,8460) | 8718.2 (8109.9,9329.4) | 3.1 (0.5,5.6) |
| Marshall Islands | 1905 (1760,2065) | 8175.3 (7594.6,8766.6) | 3728 (3432,4030) | 8476 (7879.7,9042) | 3.7 (1.3,6.2) |
| Micronesia (Federated States of) | 5110 (4740,5505) | 8313.3 (7738.6,8898.9) | 7394 (6849,8003) | 8744.1 (8145.4,9371.5) | 5.2 (2.8,7.7) |
| Nauru | 544 (502,592) | 8632.1 (8048,9264.7) | 698 (644,757) | 9105 (8473.2,9748.9) | 5.5 (3.3,7.8) |
| Niue | 186 (173,199) | 8670.5 (8041.9,9334) | 175 (162,188) | 8957.5 (8282.6,9663) | 3.3 (1.1,5.7) |
| Northern Mariana Islands | 2867 (2626,3141) | 8797.6 (8200.9,9441.4) | 4746 (4387,5099) | 9078.8 (8455.6,9714.1) | 3.2 (0.7,5.8) |
| Palau | 1027 (956,1114) | 8544.2 (7955.6,9159.8) | 1947 (1797,2108) | 8900.4 (8266.8,9595.2) | 4.2 (1.7,6.8) |
| Papua New Guinea | 179603 (165528,195405) | 7388.6 (6896.2,7935.3) | 538462 (494564,590048) | 7685.7 (7155,8282.9) | 4 (1.4,6.2) |
| Samoa | 8628 (8012,9299) | 8335.3 (7760,8940.8) | 13962 (12935,15079) | 8626.9 (8017.8,9290.7) | 3.5 (0.9,6) |
| Solomon Islands | 14747 (13720,15956) | 8066.7 (7526,8676.1) | 37933 (34962,41296) | 8231.6 (7603.5,8851.1) | 2 (-0.9,4.9) |
| Tokelau | 107 (99,114) | 8149.5 (7578,8739.1) | 121 (112,130) | 8484 (7876.5,9158.2) | 4.1 (1.7,6.9) |
| Tonga | 5372 (4996,5776) | 8442.8 (7847.4,9065.9) | 7502 (6986,8081) | 8763.8 (8180,9417.8) | 3.8 (1.3,6.2) |
| Tuvalu | 609 (566,656) | 8324.3 (7745.8,8960.2) | 948 (879,1026) | 8641.4 (8027.9,9325) | 3.8 (1.5,6.3) |
| Vanuatu | 7356 (6741,7986) | 8514.5 (7893.4,9206.9) | 19551 (18111,21165) | 8886.1 (8277.8,9532.4) | 4.4 (1.9,7.2) |
| **South Asia** | 72584182 (67087414,78502263) | 9936.6 (9252.6,10645.7) | 158803354 (147190732,171556959) | 9565.3 (8903.7,10265.7) | -3.7 (-4.7,-2.8) |
| Bangladesh | 5331909 (4950686,5772489) | 8395.6 (7832.8,8968.1) | 12882108 (11949483,13899210) | 8555.4 (7963.4,9177.3) | 1.9 (-0.5,4.5) |
| Bhutan | 31715 (29384,34240) | 9135.3 (8522.1,9749.9) | 64186 (59913,69314) | 9213.5 (8612.1,9876.6) | 0.9 (-1.5,3.3) |
| India | 60111283 (55460535,65038616) | 10258.7 (9547.9,10986.1) | 128031911 (118513994,138721278) | 9710.5 (9024.2,10420.3) | -5.3 (-6.4,-4.4) |
| Nepal | 1177468 (1101596,1257964) | 10501.2 (9843.1,11204.3) | 2762106 (2575557,3032129) | 10887.7 (10177.4,11883.1) | 3.7 (0.9,13) |
| Pakistan | 5931806 (5491196,6396491) | 8589.1 (7994.6,9202.4) | 15063043 (13945283,16289223) | 9164.6 (8522.8,9774.2) | 6.7 (5.2,8.3) |
| **Southeast Asia** | 33768821 (31068003,36689892) | 10225.2 (9474,11022.1) | 73561437 (67779796,79733436) | 10474.6 (9718.9,11301.7) | 2.4 (1.6,3.2) |
| Cambodia | 566875 (518696,615039) | 9455 (8749.4,10222.8) | 1378217 (1266270,1503301) | 9453 (8751.4,10231.3) | 0 (-2.2,2.5) |
| Indonesia | 13599739 (12496422,14810294) | 10447.7 (9661.9,11264.5) | 29413896 (26931317,32010165) | 10740.3 (9942.1,11598.8) | 2.8 (2,3.7) |
| Lao People's Democratic Republic | 277122 (254762,299243) | 10702 (9904.8,11504.6) | 621716 (570484,678042) | 10478.7 (9688,11293) | -2.1 (-4.4,0.4) |
| Malaysia | 1328704 (1222289,1442379) | 10828.3 (10076.2,11666.6) | 3443338 (3171290,3729769) | 11008.6 (10171.2,11849.4) | 1.7 (-1,4.4) |
| Maldives | 12689 (11699,13709) | 10424.9 (9701.4,11220.8) | 52048 (47116,57976) | 10202.7 (9432,11087) | -2.1 (-4.6,0.4) |
| Mauritius | 96542 (88907,105229) | 10712.2 (9939.1,11620.8) | 189008 (175935,203155) | 11411.6 (10649.1,12263.7) | 6.5 (3.3,10) |
| Myanmar | 2982783 (2758045,3235228) | 10357.2 (9596.6,11185.4) | 5373282 (4929525,5841826) | 10173 (9393.1,11017.3) | -1.8 (-4.2,0.8) |
| Philippines | 4286723 (3941958,4660864) | 10549.8 (9776.7,11356.1) | 10193697 (9395294,11071571) | 10530.4 (9775.3,11372.9) | -0.2 (-0.9,0.4) |
| Seychelles | 6521 (6032,7044) | 10613 (9840.2,11453.2) | 12837 (11856,13936) | 10884.5 (10070.5,11753) | 2.6 (-0.3,5.5) |
| Sri Lanka | 1386481 (1270757,1506959) | 10274.6 (9504.3,11118.7) | 2647885 (2443533,2855363) | 10450.5 (9660.4,11299.2) | 1.7 (-0.7,4.5) |
| Thailand | 4699377 (4333121,5101891) | 10448.1 (9717.1,11188.5) | 10328746 (9637500,11140578) | 10839.2 (10107.8,11724.9) | 3.7 (1.2,6.6) |
| Timor-Leste | 45151 (41110,49397) | 9936.4 (9194.9,10715.2) | 104170 (96163,112715) | 10410.8 (9601.8,11301.7) | 4.8 (2.3,7.3) |
| Viet Nam | 4431264 (4056446,4826467) | 9094.4 (8336.9,9858.1) | 9699996 (8886323,10558442) | 9152.1 (8434.8,9895.8) | 0.6 (-1.8,3.2) |
| **Southern Latin America** | 2695843 (2510441,2883801) | 5784.4 (5397.3,6182.9) | 4860631 (4530777,5214539) | 5970.4 (5543.1,6415) | 3.2 (0.7,6.1) |
| Uruguay | 221996 (206709,237075) | 6192.6 (5767.9,6613.6) | 301874 (280956,322913) | 6282.8 (5818,6738.2) | 1.5 (-2,4.6) |
| Argentina | 1774583 (1648138,1900776) | 5585.8 (5197.5,5980) | 3114327 (2893145,3345866) | 5876.1 (5442.4,6323.9) | 5.2 (1.8,9.7) |
| Chile | 699136 (650457,755320) | 6211 (5786.9,6653.9) | 1444161 (1343232,1545985) | 6106.6 (5659.6,6545.9) | -1.7 (-4.8,1.4) |
| **Southern Sub-Saharan Africa** | 2945199 (2740170,3170275) | 8946.4 (8370.4,9563.7) | 5972951 (5547456,6415524) | 9037.9 (8440.6,9647.1) | 1 (-0.2,2.4) |
| Botswana | 60835 (56449,65830) | 8684.7 (8075.9,9285.6) | 164894 (153184,177219) | 8867.3 (8244.1,9441.2) | 2.1 (-0.6,5) |
| Eswatini | 33811 (31458,36377) | 8783.2 (8183.6,9417.6) | 66871 (61736,72023) | 9047.6 (8414.1,9624.3) | 3 (0.3,5.8) |
| Lesotho | 79823 (74328,85461) | 8435.2 (7898.7,8992.7) | 114258 (105784,123018) | 8824.6 (8201.2,9408.8) | 4.6 (1.7,7.6) |
| Namibia | 68175 (63117,73308) | 8664.4 (8056.5,9233.5) | 144692 (133912,155904) | 8510.1 (7908.4,9112.4) | -1.8 (-4.1,0.9) |
| South Africa | 2257302 (2099020,2428551) | 9062.8 (8465.3,9681) | 4677573 (4347292,5025759) | 9097.4 (8501.9,9729.1) | 0.4 (-0.9,1.8) |
| Zimbabwe | 445253 (413595,477795) | 8575.2 (7995.1,9192.8) | 804662 (746942,870315) | 8832.9 (8243,9439.3) | 3 (0.4,5.7) |
| **Tropical Latin America** | 8408163 (7820461,9015639) | 7763 (7240.7,8281.3) | 19295703 (18022781,20680042) | 7576.2 (7081,8107.5) | -2.4 (-3.4,-1.4) |
| Brazil | 8215420 (7640129,8812335) | 7772.7 (7248.7,8291.2) | 18814692 (17566365,20174535) | 7576.6 (7078.5,8111.2) | -2.5 (-3.6,-1.5) |
| Paraguay | 192742 (179980,206867) | 7374.3 (6876.7,7891.9) | 481010 (448476,516329) | 7561.4 (7051.6,8091.3) | 2.5 (-0.8,6.7) |
| **Western Europe** | 29093594 (27230515,30911868) | 5469.9 (5132.6,5799.4) | 41591466 (39070950,43910754) | 5226.2 (4924.4,5544.2) | -4.5 (-6.2,-3) |
| Andorra | 3305 (3059,3549) | 5604.9 (5216.1,5996.5) | 7623 (7073,8129) | 5535 (5147.9,5919.3) | -1.2 (-4.2,1.4) |
| Austria | 611202 (567555,653621) | 5590.9 (5205.7,5978.5) | 892225 (838208,953902) | 5671 (5326.4,6084.5) | 1.4 (-2.8,5.8) |
| Belgium | 808609 (750019,867640) | 5722.1 (5342.5,6109.5) | 1120459 (1051828,1196504) | 5633.2 (5305.5,6009.4) | -1.6 (-5.9,2.8) |
| Cyprus | 47988 (44580,51332) | 6009 (5608.3,6395) | 112031 (104079,120582) | 5817.6 (5430.8,6247.9) | -3.2 (-6,-0.4) |
| Denmark | 415234 (386838,442513) | 5589.5 (5229.3,5953.9) | 564571 (524056,607172) | 5607 (5237.4,6009.8) | 0.3 (-3.9,4.8) |
| Finland | 368157 (345612,392962) | 5556.6 (5209.3,5940.6) | 555910 (513129,595333) | 5349.2 (4968.6,5714.9) | -3.7 (-6.7,-0.7) |
| France | 3209806 (2980224,3426824) | 4202.2 (3898.7,4488.2) | 5214795 (4858423,5614715) | 4368.8 (4085.6,4698.7) | 4 (-0.3,8.5) |
| Germany | 6261066 (5866849,6644060) | 5349.6 (5017.4,5675) | 8931944 (8467049,9441979) | 5278.4 (4985.5,5622.2) | -1.3 (-5.3,2.1) |
| Greece | 798820 (740666,853941) | 5762.3 (5361.4,6162.9) | 1143247 (1066167,1220356) | 5587.8 (5236.3,5992.8) | -3 (-6.9,0.8) |
| Iceland | 13378 (12516,14287) | 4739 (4420.2,5063.2) | 23128 (21625,24673) | 4528.9 (4231,4840.2) | -4.4 (-7.3,-1.6) |
| Ireland | 261985 (241344,282388) | 6681.7 (6202.5,7184.3) | 465353 (440528,488132) | 6594.1 (6237.5,6952) | -1.3 (-6.2,4.1) |
| Israel | 288923 (270285,308809) | 6007.8 (5618.9,6420.3) | 674873 (630913,722204) | 5866.8 (5487.9,6273.7) | -2.3 (-6.3,1.9) |
| Italy | 4457622 (4142484,4769497) | 5591.9 (5235.5,5978.8) | 5921997 (5501809,6294811) | 5061.3 (4746.2,5410.5) | -9.5 (-11.1,-7.5) |
| Luxembourg | 30243 (27852,32537) | 5937.6 (5491.4,6400.1) | 55107 (50921,59381) | 5775.6 (5340.1,6220.8) | -2.7 (-6.4,0.6) |
| Malta | 24308 (22689,26237) | 5881.7 (5499.5,6324.9) | 46498 (43037,49500) | 5665.8 (5292.2,6053.1) | -3.7 (-6.7,-0.6) |
| Monaco | 3295 (3048,3530) | 5539.9 (5150.2,5916.3) | 4350 (4042,4640) | 5497.2 (5141.2,5862.9) | -0.8 (-3.8,2.1) |
| Netherlands | 1147619 (1073580,1226033) | 6067.4 (5675.9,6498.2) | 1745668 (1634215,1877107) | 6048.2 (5674.3,6481.1) | -0.3 (-3.2,2.9) |
| Norway | 314586 (291897,335963) | 5207.1 (4866.3,5562) | 469377 (437256,500382) | 5534.4 (5167.8,5922.5) | 6.3 (5.1,7.5) |
| Portugal | 641583 (596339,685707) | 5124.5 (4804.7,5501.1) | 941606 (850524,1011350) | 4777 (4420.4,5132.3) | -6.8 (-12.1,-3.6) |
| San Marino | 1771 (1651,1899) | 5453.5 (5097.1,5832.3) | 3397 (3165,3648) | 5392.6 (5034.8,5768.8) | -1.1 (-3.7,1.6) |
| Spain | 2768974 (2588950,2935877) | 5464.9 (5131.1,5781.9) | 4288430 (3948305,4576051) | 5022.8 (4691,5348.8) | -8.1 (-14.4,-4.4) |
| Sweden | 821047 (759582,881515) | 6415.6 (5943,6890.7) | 1121779 (1040388,1207272) | 6424 (5973.5,6887.8) | 0.1 (-2.1,2.4) |
| Switzerland | 639460 (600031,681319) | 6582.8 (6179,7026.2) | 995630 (934109,1061548) | 6354 (5957.2,6788.6) | -3.5 (-6.5,-0.2) |
| United Kingdom | 5130703 (4779037,5480493) | 6119.8 (5722.5,6520.2) | 6254851 (5837368,6635678) | 5449.1 (5106.2,5788.7) | -11 (-12.2,-9.7) |
| **Western Sub-Saharan Africa** | 9003658 (8381320,9663357) | 8310.7 (7767.4,8851.6) | 22472497 (20882526,24191820) | 8324.3 (7782.3,8871) | 0.2 (-0.9,1.2) |
| Benin | 188015 (174766,201634) | 7343.4 (6857.5,7853.5) | 552484 (512421,599062) | 7482.3 (7003.2,8021.9) | 1.9 (-0.8,4.6) |
| Burkina Faso | 365246 (337985,392297) | 7076.2 (6568.6,7570.5) | 901049 (834507,973982) | 7181.8 (6699.9,7686.7) | 1.5 (-1,4) |
| Cabo Verde | 17443 (16294,18677) | 7283.3 (6802.8,7767.9) | 38020 (35307,41075) | 7366.7 (6831.1,7880.3) | 1.1 (-1.3,3.4) |
| Cameroon | 463067 (431742,496225) | 8270.7 (7742.1,8827.3) | 1594660 (1486358,1713045) | 9043.6 (8503.4,9609.8) | 9.3 (6.2,12.5) |
| Chad | 241571 (223146,258353) | 7195.1 (6688.4,7683.2) | 603512 (558192,650782) | 7271 (6764.7,7787.6) | 1.1 (-1.5,3.8) |
| Gambia | 36537 (33712,39478) | 7285.8 (6764.4,7776.6) | 103566 (95771,112154) | 7469.2 (6954.6,8002.9) | 2.5 (-0.2,5.3) |
| Ghana | 516871 (472923,562875) | 5943.1 (5509.3,6423.8) | 1422793 (1305227,1553890) | 6033.8 (5584.9,6513.2) | 1.5 (-0.7,4.2) |
| Guinea | 275726 (256995,296333) | 7313.9 (6820.2,7803.8) | 568301 (528813,612479) | 7498.3 (6998.8,7995.5) | 2.5 (0.2,5.1) |
| Guinea-Bissau | 40061 (37305,43253) | 7641.8 (7095.7,8167.3) | 85214 (78709,92411) | 7643.8 (7104.1,8145.4) | 0 (-2.4,2.5) |
| Liberia | 107783 (99699,116096) | 7548.2 (7002.1,8076.2) | 244755 (226016,265719) | 7551.2 (7049.8,8085.2) | 0 (-2.3,2.4) |
| Mali | 358053 (333871,381898) | 7349.8 (6875.5,7816.7) | 909702 (841914,979718) | 7419.9 (6929.5,7932.6) | 1 (-1.5,3.4) |
| Mauritania | 91156 (84420,98252) | 7479.4 (6957.7,8019.8) | 197970 (183860,213616) | 7285.5 (6794.6,7783.8) | -2.6 (-4.9,-0.3) |
| Niger | 265038 (246140,284905) | 6880.2 (6411.5,7331.4) | 790430 (731816,852136) | 6881.8 (6387.8,7357.1) | 0 (-2.9,2.6) |
| Nigeria | 4951044 (4609650,5305923) | 9402.3 (8798.4,10034.7) | 11730967 (10926433,12598672) | 9488.9 (8881.1,10113.3) | 0.9 (-0.2,2.1) |
| Sao Tome and Principe | 5810 (5424,6228) | 7967.4 (7434.1,8520.1) | 12272 (11408,13236) | 8194.6 (7639.2,8749.2) | 2.9 (0,5.9) |
| Senegal | 294300 (271578,318380) | 6986.3 (6472.3,7529.8) | 679249 (627366,734863) | 6614 (6185.7,7067.8) | -5.3 (-7.6,-2.6) |
| Sierra Leone | 191300 (177895,206334) | 7521.3 (7006.4,8070.4) | 405349 (372950,439223) | 7610.6 (7071.5,8155.3) | 1.2 (-1.1,3.7) |
| Togo | 131761 (121749,142772) | 7346 (6804,7869) | 382466 (353706,416128) | 7283.9 (6781.1,7815.9) | -0.8 (-3.4,1.5) |
| CÃ ́te d'Ivoire | 462576 (427133,500797) | 7562 (7049.4,8106.4) | 1249475 (1157292,1360894) | 7597.7 (7075.2,8139.2) | 0.5 (-2.1,2.9) |
