# Supplementary material for: Burden of chronic kidney disease and its attributable risk factors in 204 countries and territories, 1990–2021: Results from the Global Burden of Disease study 2021
Source: Medicine (Baltimore). 2026 Aug 7;105(32):e50111. doi: 10.1097/MD.0000000000050111 (PMC13456769; doi:10.1097/MD.0000000000050111)
Supplement: Supplementary file 4 [file medi-105-e50111-s006.docx]

**Table S4.** Deaths due to chronic kidney disease in 1990 and 2021 and the percentage change in the age-standardised rates (ASRs) per 100,000, by location.

|  | **1990** | | **2021** | | **Percentage change in the ASRs per 100,000** |
| --- | --- | --- | --- | --- | --- |
|  | **No (95% UI)** | **ASRs per 100,000 (95% UI)** | **No (95% UI)** | **ASRs per 100,000 (95% UI)** |  |
| **Global** | 552673 (513463,607915) | 14.9 (13.6,16.4) | 1527639 (1389377,1638914) | 18.5 (16.7,19.9) | 24.5 (10.2,33.5) |
| **Andean Latin America** | 5795 (5288,6456) | 28.5 (25.9,31.9) | 21619 (17903,25800) | 37.7 (31.3,44.9) | 32 (7.7,63.3) |
| Bolivia (Plurinational State of) | 1325 (1115,1675) | 43 (36.2,53.9) | 4731 (3675,6140) | 58.2 (45.7,75.3) | 35.5 (-0.3,86.9) |
| Ecuador | 1227 (1150,1287) | 23.7 (22.1,25) | 6502 (4898,8822) | 42.1 (32.2,56) | 77.5 (35.9,133.3) |
| Peru | 3242 (2796,3748) | 27 (23.3,31.4) | 10386 (7395,13295) | 30.9 (22.1,39.7) | 14.6 (-18.4,51.8) |
| **Australasia** | 1851 (1687,1959) | 8.5 (7.7,9) | 5968 (5025,6525) | 9.6 (8.2,10.5) | 13.7 (6.1,20.4) |
| Australia | 1580 (1436,1674) | 8.7 (7.9,9.3) | 5011 (4201,5515) | 9.4 (8,10.3) | 7.9 (0,15.4) |
| New Zealand | 271 (249,288) | 7.3 (6.6,7.7) | 957 (835,1041) | 10.7 (9.4,11.6) | 47.1 (37.1,58.7) |
| **Caribbean** | 4754 (4399,5437) | 18.7 (17.3,21.3) | 13888 (12086,16193) | 25.8 (22.4,30.1) | 37.6 (18.9,57.5) |
| Antigua and Barbuda | 17 (16,18) | 30.1 (27.9,32.2) | 46 (42,49) | 47.1 (43,50.5) | 56.3 (41.5,73.4) |
| Bahamas | 37 (34,40) | 23.1 (21.3,25.1) | 144 (117,178) | 37.5 (30.7,46.2) | 62.4 (30.1,104.3) |
| Barbados | 60 (57,64) | 20.4 (19.1,21.4) | 148 (117,182) | 29.3 (23.3,36) | 43.8 (14.3,78.1) |
| Belize | 24 (23,26) | 24.4 (22.8,26) | 134 (117,151) | 45.5 (39.5,51.1) | 86.3 (61.3,110.6) |
| Bermuda | 9 (9,10) | 15.5 (14.5,16.5) | 23 (19,27) | 15.7 (13.2,19) | 1 (-15,22) |
| Cuba | 831 (789,866) | 8.3 (7.9,8.7) | 3209 (2758,3638) | 16.1 (13.9,18.3) | 94.3 (69.1,121.4) |
| Dominica | 19 (16,21) | 32.4 (28.2,36.3) | 40 (32,49) | 50.3 (40.5,61.8) | 55.4 (25.3,92.7) |
| Dominican Republic | 621 (533,750) | 16.7 (14.3,20.3) | 2392 (1646,3064) | 24 (16.5,30.7) | 43.9 (-5.6,90.9) |
| Grenada | 27 (24,29) | 35.7 (32.5,39.2) | 62 (54,69) | 58.3 (51,64.9) | 63.2 (40,87.3) |
| Guyana | 117 (106,128) | 30.5 (27.7,33.4) | 349 (269,446) | 56.5 (43.8,71.2) | 85.3 (39.7,133.1) |
| Haiti | 962 (707,1555) | 28.7 (20.5,48.9) | 2177 (1207,4587) | 30.9 (17.2,64.8) | 7.6 (-31.2,52.4) |
| Jamaica | 434 (408,457) | 23.3 (21.9,24.6) | 935 (707,1205) | 29.2 (22,37.5) | 24.9 (-5.1,62.3) |
| Puerto Rico | 1091 (1031,1148) | 31.8 (29.9,33.4) | 2537 (2093,2995) | 32.1 (26.6,37.9) | 1.2 (-15.9,18.5) |
| Saint Kitts and Nevis | 16 (15,17) | 43.6 (40.5,46.8) | 34 (28,40) | 57.6 (48.5,65.8) | 32.1 (9.6,52.8) |
| Saint Lucia | 30 (29,32) | 37.3 (35.3,39.2) | 93 (77,111) | 40.3 (33.5,47.9) | 8 (-11.2,27.6) |
| Saint Vincent and the Grenadines | 19 (17,20) | 26.9 (24.9,28.7) | 54 (48,61) | 41.1 (36.2,46.4) | 52.7 (32.3,74.3) |
| Suriname | 71 (61,81) | 27.7 (24.2,31.8) | 253 (189,329) | 41.4 (31,53.6) | 49.6 (6.9,101.1) |
| Trinidad and Tobago | 192 (184,201) | 24.7 (23.5,25.8) | 755 (564,967) | 40.2 (30.1,51.3) | 62.6 (22,108.7) |
| United States Virgin Islands | 15 (12,19) | 19.9 (16.6,24) | 33 (23,43) | 20.5 (14.7,26.6) | 2.9 (-32.5,44) |
| **Central Asia** | 2536 (2250,2933) | 5 (4.4,5.9) | 9397 (8278,10515) | 12.1 (10.7,13.5) | 141.9 (101.3,186.6) |
| Armenia | 27 (22,31) | 1 (0.8,1.1) | 432 (354,523) | 10.3 (8.4,12.4) | 953.1 (706,1362.9) |
| Azerbaijan | 357 (277,452) | 6.6 (5.1,8.3) | 992 (717,1295) | 10.4 (7.6,13.4) | 57.8 (14.7,127.4) |
| Georgia | 197 (161,247) | 3.3 (2.7,4.1) | 713 (574,898) | 12.3 (9.9,15.5) | 272.5 (194,355.8) |
| Kazakhstan | 737 (644,832) | 5.3 (4.6,6) | 1982 (1631,2335) | 12.2 (10.1,14.4) | 129.1 (83,181.9) |
| Kyrgyzstan | 159 (141,180) | 4.6 (4,5.2) | 498 (415,596) | 10 (8.3,11.9) | 118.4 (74.7,169.5) |
| Mongolia | 173 (139,213) | 15.4 (12.4,18.9) | 296 (236,365) | 13.6 (10.8,16.8) | -11.5 (-34.5,15.1) |
| Tajikistan | 59 (48,83) | 1.8 (1.4,2.6) | 152 (106,220) | 2.4 (1.7,3.3) | 34.2 (-7.7,93.9) |
| Turkmenistan | 209 (191,228) | 8.9 (8.1,9.8) | 744 (574,947) | 18 (14,22.7) | 101.4 (53.2,162.9) |
| Uzbekistan | 617 (441,920) | 5 (3.4,7.9) | 3589 (3013,4204) | 14.2 (12.1,16.7) | 182.1 (79,327.7) |
| **Central Europe** | 14335 (13732,14892) | 10.5 (10,10.9) | 21635 (19445,23977) | 9.4 (8.4,10.5) | -10.4 (-18.3,-1.7) |
| Albania | 234 (196,277) | 12 (10,14.3) | 424 (322,541) | 10.6 (8.1,13.4) | -11.6 (-34.7,19.9) |
| Bosnia and Herzegovina | 361 (307,422) | 9.8 (8.3,11.4) | 686 (512,869) | 11 (8.3,13.9) | 12.5 (-17.4,45.7) |
| Bulgaria | 729 (668,785) | 7.6 (7,8.2) | 2428 (2049,2872) | 17.4 (14.6,20.8) | 127.8 (87.6,171.4) |
| Croatia | 502 (464,540) | 9.5 (8.8,10.2) | 1337 (1158,1549) | 13.5 (11.6,15.7) | 42.2 (21.9,68.5) |
| Czechia | 1096 (999,1200) | 8.2 (7.5,8.9) | 1562 (1326,1820) | 6.8 (5.8,8) | -16.8 (-30.1,-1.3) |
| Hungary | 842 (795,888) | 6.3 (5.9,6.6) | 2173 (1852,2483) | 10.1 (8.7,11.6) | 61.8 (40.6,86.1) |
| Montenegro | 76 (61,93) | 12.9 (10.4,15.8) | 165 (124,208) | 19.1 (14,23.9) | 47.8 (-0.5,103.1) |
| North Macedonia | 173 (145,208) | 10.1 (8.5,12.2) | 370 (282,484) | 13.6 (10.7,17.3) | 34.8 (0.7,78.6) |
| Poland | 5309 (5123,5460) | 12.8 (12.3,13.2) | 4981 (4384,5654) | 6.6 (5.8,7.6) | -48.5 (-53.7,-42) |
| Romania | 2551 (2408,2682) | 10.4 (9.8,10.9) | 3325 (2869,3831) | 8.6 (7.4,10) | -17.3 (-29.8,-3.2) |
| Serbia | 1464 (1214,1828) | 16.5 (13.6,20.8) | 2713 (2196,3252) | 15.9 (12.8,19.1) | -3.7 (-30.8,27.4) |
| Slovakia | 617 (530,708) | 10.6 (9.1,12.2) | 831 (677,1002) | 8.9 (7.3,10.7) | -16 (-32.9,3) |
| Slovenia | 152 (142,163) | 6.4 (5.9,6.9) | 327 (261,415) | 6.1 (4.9,7.8) | -4.3 (-21.2,18.1) |
| **Central Latin America** | 22182 (21513,22820) | 27.9 (26.8,28.8) | 104444 (94171,116461) | 42.4 (38.3,47) | 51.6 (37.5,66.7) |
| Colombia | 3340 (3177,3471) | 19.5 (18.4,20.4) | 8646 (7184,10116) | 15.5 (12.9,18.1) | -20.7 (-33,-8) |
| Costa Rica | 264 (247,279) | 15.3 (14.2,16.2) | 1605 (1391,1794) | 29 (25.2,32.3) | 89.7 (69.5,112.5) |
| El Salvador | 797 (702,1141) | 25.3 (22.1,36.3) | 4441 (3314,5504) | 69.8 (52.2,86.8) | 176.3 (60.6,259.3) |
| Guatemala | 1108 (1063,1158) | 33.7 (32,35.3) | 5448 (4533,6387) | 50.7 (42.3,59.3) | 50.4 (23.7,78) |
| Honduras | 259 (215,328) | 12.3 (10.1,16) | 1439 (1077,1889) | 24.6 (18.8,31.7) | 99.5 (27,172.3) |
| Mexico | 14133 (13701,14565) | 36.9 (35.4,38.4) | 67044 (58757,76983) | 54.3 (47.8,61.9) | 47 (29.7,64.9) |
| Nicaragua | 492 (444,663) | 29.4 (26.2,40.1) | 2686 (2148,3230) | 55.3 (44.7,66.5) | 88.4 (29.6,136) |
| Panama | 201 (188,215) | 13.5 (12.6,14.5) | 1381 (1090,1653) | 30.7 (24.3,36.8) | 127.6 (80.4,176.6) |
| Venezuela (Bolivarian Republic of) | 1587 (1501,1651) | 16.3 (15.2,17.1) | 11753 (8833,14984) | 40.5 (30.4,51.3) | 148.2 (90.2,213.8) |
| **Central Sub-Saharan Africa** | 8667 (7200,10419) | 42.7 (35.5,51) | 21011 (16114,27467) | 43.7 (33.3,56.3) | 2.4 (-22.2,33.4) |
| Angola | 1417 (1093,1793) | 37.6 (29.3,46.9) | 4073 (2988,5384) | 39.2 (27.9,50.3) | 4.1 (-24.4,40.1) |
| Central African Republic | 507 (420,615) | 47 (38.6,56.4) | 963 (698,1352) | 47.4 (35.4,63.9) | 0.9 (-22.7,29.8) |
| Congo | 531 (406,637) | 55.8 (41,67.2) | 1252 (847,1626) | 54.8 (35.6,69) | -1.9 (-22.8,21.9) |
| Democratic Republic of the Congo | 5868 (4671,7377) | 42.3 (34.5,52) | 13845 (9966,18759) | 42.9 (31.4,57.7) | 1.7 (-28.2,42.4) |
| Equatorial Guinea | 79 (62,98) | 43.8 (35,53.4) | 255 (156,372) | 54.7 (31.3,76.5) | 25 (-27.8,84.7) |
| Gabon | 265 (212,318) | 51.1 (40.7,61.6) | 621 (324,822) | 71.5 (35.5,93) | 40 (-15.4,87) |
| **East Asia** | 107701 (94477,126024) | 14.4 (12.7,17) | 217342 (178047,259057) | 11.1 (9.2,13.2) | -22.5 (-39.2,-5.5) |
| China | 102726 (89745,120908) | 14.2 (12.4,16.8) | 204230 (164736,246372) | 10.8 (8.8,13) | -23.4 (-41,-5.7) |
| Democratic People's Republic of Korea | 2021 (1438,2685) | 14 (10.1,19.3) | 4298 (3284,5596) | 14 (10.7,18.3) | -0.5 (-29.5,40) |
| Taiwan (Province of China) | 2954 (2802,3083) | 24.1 (22.5,25.4) | 8814 (7593,9771) | 20 (17.4,22.1) | -17.1 (-24.6,-9.8) |
| **Eastern Europe** | 9389 (9183,9574) | 3.6 (3.5,3.7) | 17821 (16126,19853) | 5.2 (4.7,5.8) | 45.9 (33.3,59.9) |
| Belarus | 100 (93,106) | 0.8 (0.8,0.9) | 360 (296,427) | 2.4 (1.9,2.8) | 182.4 (130,239.1) |
| Estonia | 120 (112,127) | 6.4 (6,6.8) | 527 (437,605) | 17 (14.2,19.7) | 166.6 (125.1,206.6) |
| Latvia | 95 (89,101) | 2.9 (2.7,3.1) | 321 (265,392) | 7.7 (6.4,9.5) | 166.7 (118.1,224.5) |
| Lithuania | 104 (97,109) | 2.5 (2.3,2.6) | 332 (281,400) | 5.8 (4.9,7.1) | 136 (98,182.8) |
| Republic of Moldova | 83 (79,87) | 2 (1.9,2.1) | 206 (179,242) | 3.6 (3.2,4.2) | 78.7 (56.2,109.6) |
| Russian Federation | 8804 (8612,8979) | 5.1 (5,5.2) | 14471 (13013,16149) | 6.2 (5.6,6.9) | 20.2 (9.3,32.5) |
| Ukraine | 84 (80,88) | 0.1 (0.1,0.1) | 1605 (1204,2049) | 2.4 (1.8,3.1) | 1714.7 (1257.3,2220.9) |
| **Eastern Sub-Saharan Africa** | 30615 (26919,35172) | 42.4 (37.1,50.2) | 60918 (53817,69935) | 40.1 (35.6,46) | -5.5 (-18.6,8.4) |
| Burundi | 909 (711,1148) | 39.5 (31,50.6) | 1515 (1109,2172) | 35 (25.8,49.9) | -11.4 (-34.1,18.6) |
| Comoros | 70 (54,88) | 39 (30.8,48) | 182 (132,235) | 42.3 (31,54.4) | 8.4 (-20.2,47.2) |
| Djibouti | 35 (26,47) | 29.3 (22.4,37.7) | 211 (152,285) | 41.8 (31.4,54.7) | 42.5 (-3,99.6) |
| Eritrea | 358 (267,498) | 32.6 (24.7,45.5) | 902 (610,1462) | 38.1 (26.6,60.6) | 16.7 (-8.6,54.5) |
| Ethiopia | 12912 (10097,14949) | 69 (57,79.6) | 16918 (14059,20068) | 42.4 (34.8,50.2) | -38.6 (-50.1,-25.5) |
| Kenya | 1950 (1548,3039) | 25.2 (19.6,40.8) | 7330 (5884,9860) | 37.6 (29.8,49.5) | 49.1 (12.8,92.1) |
| Madagascar | 1300 (1031,1793) | 26.7 (21,38.2) | 2660 (1941,3524) | 27 (19.7,35.4) | 1 (-28,34.3) |
| Malawi | 1438 (1170,1736) | 38.7 (31.2,49) | 3141 (2543,3818) | 45.4 (37.8,54.5) | 17.5 (-12.9,48.5) |
| Mozambique | 1433 (1145,1942) | 26 (20.8,37.4) | 3627 (2744,4922) | 35.7 (26.9,48.8) | 37 (-5.2,85.7) |
| Rwanda | 1301 (1059,1534) | 47.9 (39.1,57) | 2068 (1481,2765) | 38.7 (28,51.2) | -19.3 (-41.9,8.3) |
| Somalia | 1051 (755,1454) | 45.7 (33.4,63.2) | 2791 (1917,4130) | 47.9 (33.1,68.3) | 4.9 (-19.6,35.3) |
| South Sudan | 1009 (753,1391) | 40.9 (31,56.2) | 1949 (1428,2539) | 55 (39.9,71.2) | 34.3 (-14.7,86.9) |
| Uganda | 1868 (1404,2443) | 31 (23.5,40.7) | 5279 (4093,6903) | 38.8 (30.8,50.8) | 25.3 (-8,75) |
| United Republic of Tanzania | 3665 (3049,4635) | 36.1 (30.3,46.2) | 8860 (7121,11155) | 37.7 (30.1,46.9) | 4.4 (-22.1,38.5) |
| Zambia | 1293 (1071,1518) | 46.3 (39.5,54.3) | 3432 (2424,4962) | 52.2 (39.2,69.8) | 12.8 (-17.4,60.7) |
| **High-income Asia Pacific** | 22233 (20303,23333) | 12.6 (11.3,13.3) | 63459 (50432,70953) | 9.7 (8.1,10.7) | -22.8 (-28.7,-18.6) |
| Brunei Darussalam | 32 (26,42) | 37.5 (30.7,47.8) | 86 (73,100) | 36.3 (30.6,42.3) | -3.2 (-24.4,21) |
| Japan | 17679 (16010,18557) | 11.6 (10.4,12.3) | 52717 (41038,59205) | 9.5 (7.8,10.4) | -18.3 (-24.2,-14.1) |
| Republic of Korea | 4257 (3775,4740) | 18.6 (16.3,21.4) | 9823 (7902,11473) | 10.8 (8.7,12.7) | -41.8 (-53,-31.5) |
| Singapore | 264 (250,275) | 13.7 (12.8,14.4) | 833 (718,926) | 10.2 (8.7,11.3) | -25.9 (-33.4,-18.5) |
| **High-income North America** | 29949 (27171,31433) | 8.3 (7.6,8.7) | 143679 (124827,154757) | 20.6 (18.1,22) | 147 (139.1,155.4) |
| Canada | 2119 (1921,2236) | 6.8 (6.1,7.2) | 7789 (6588,8473) | 9.5 (8.2,10.3) | 40.1 (29.5,49.2) |
| Greenland | 3 (3,4) | 13.1 (11,15.3) | 8 (6,10) | 14.8 (10.7,18.6) | 12.9 (-13.9,46.8) |
| United States of America | 27826 (25245,29210) | 8.5 (7.7,8.9) | 135880 (118167,146606) | 21.9 (19.3,23.5) | 158.3 (149.6,167.9) |
| **North Africa and Middle East** | 45472 (37703,66491) | 31.2 (25.5,47) | 144687 (126153,162699) | 37.7 (32.7,42.4) | 20.8 (-23.7,50.5) |
| Afghanistan | 3009 (2175,4675) | 47.3 (34.5,75.9) | 4893 (2926,8625) | 53.9 (32.9,102.2) | 13.9 (-30.7,75.1) |
| Algeria | 2344 (1667,4128) | 27.1 (19,47.7) | 9785 (7909,12092) | 37.3 (30.6,45.5) | 37.6 (-25.2,107.8) |
| Bahrain | 48 (36,63) | 41.7 (31.3,55.7) | 259 (207,316) | 52.5 (42.1,63.4) | 25.9 (-22.3,87.8) |
| Egypt | 10610 (8478,16104) | 51.8 (40.3,82.6) | 33311 (27183,40816) | 71.7 (59.7,86.6) | 38.6 (-16.6,85.7) |
| Iran (Islamic Republic of) | 3718 (2816,5017) | 17.8 (13,24.7) | 13627 (11262,15175) | 20.2 (16.5,22.6) | 13.6 (-26.4,43.6) |
| Iraq | 2948 (2325,3775) | 36.7 (28.7,47.6) | 8279 (5635,10644) | 43.4 (30.5,54.8) | 18.3 (-29.7,66.6) |
| Jordan | 360 (293,470) | 31.6 (25.6,41.5) | 1876 (1492,2406) | 33.1 (26.2,41.8) | 4.8 (-29.2,41) |
| Kuwait | 160 (144,178) | 30.7 (26.6,34.3) | 374 (276,459) | 16 (11.7,19.7) | -47.9 (-59.7,-35.3) |
| Lebanon | 592 (455,790) | 32.3 (24.7,42.7) | 2102 (1733,2534) | 31.9 (26.3,38.5) | -1.2 (-28.7,36.4) |
| Libya | 503 (374,704) | 28.1 (20.5,40.1) | 2127 (1413,2796) | 46.9 (31.3,61.1) | 67.1 (-18.3,163.9) |
| Morocco | 3070 (2131,5653) | 23.1 (15.7,45.6) | 11890 (8911,15306) | 40 (30.3,51.3) | 73 (-2.2,152.9) |
| Oman | 140 (102,207) | 22.9 (16.6,33.8) | 616 (490,771) | 41.7 (33,50.4) | 82.1 (13.1,164.9) |
| Palestine | 348 (260,460) | 45.4 (34.2,61.6) | 838 (711,1010) | 43.2 (36.7,51.9) | -4.8 (-32.7,31.6) |
| Qatar | 28 (16,43) | 40.7 (22.7,66) | 198 (153,254) | 39.9 (32.4,49.2) | -2 (-40,73.3) |
| Saudi Arabia | 2408 (1798,3342) | 45.1 (33.5,64.1) | 12226 (9096,15498) | 79.3 (59.8,95.7) | 75.8 (-4,151.8) |
| Sudan | 2043 (1462,3607) | 22.7 (15.9,43.5) | 5602 (4127,7495) | 32.1 (24.3,43.8) | 41 (-21.9,121.7) |
| Syrian Arab Republic | 1951 (1524,2563) | 39.1 (30.4,52.8) | 4836 (3387,6504) | 47.7 (33.6,62.8) | 22 (-32.2,80.6) |
| Tunisia | 926 (706,1392) | 22.2 (17,33.3) | 3765 (2674,5162) | 32.1 (23.1,43.9) | 44.6 (-15,111.6) |
| Turkey | 9286 (7515,12586) | 30.6 (24.5,42.5) | 24805 (19764,30524) | 29.5 (23.3,36.4) | -3.5 (-35.5,26.8) |
| United Arab Emirates | 83 (57,119) | 23.1 (16,32.6) | 672 (427,914) | 37.5 (20.9,49.2) | 62.8 (-11.2,133.6) |
| Yemen | 871 (580,1629) | 19.1 (12.7,38.9) | 2471 (1678,4179) | 20.6 (14.2,34.6) | 7.8 (-26.8,59.5) |
| **Oceania** | 489 (342,661) | 17.2 (12.8,23.1) | 1546 (1265,1892) | 21.6 (18,26.4) | 25.2 (-11.7,78.6) |
| American Samoa | 7 (5,10) | 33.5 (24.6,48.1) | 31 (24,39) | 73.8 (57.4,91.7) | 120.1 (35,244.4) |
| Cook Islands | 2 (2,3) | 17.5 (13.8,23.2) | 5 (4,7) | 22 (16.6,27.2) | 25.8 (-19.8,81.4) |
| Fiji | 95 (67,136) | 27.7 (19.6,40.5) | 314 (231,410) | 48 (36.4,61.9) | 73.1 (-4.3,187.2) |
| Guam | 14 (12,17) | 22.9 (20,28.3) | 51 (42,58) | 24 (20.4,27.2) | 4.9 (-19.2,26.4) |
| Kiribati | 11 (9,13) | 30 (24.2,36.8) | 29 (18,42) | 43.1 (28,62.3) | 43.9 (-13.2,125.4) |
| Marshall Islands | 5 (3,9) | 32.5 (20.2,58.7) | 17 (6,49) | 54.8 (17.4,160.8) | 68.4 (-25.8,175.1) |
| Micronesia (Federated States of) | 16 (11,25) | 33.5 (22.8,54.5) | 38 (27,53) | 57.6 (42.1,80.9) | 71.7 (4.1,175.3) |
| Nauru | 2 (1,3) | 27.8 (20.8,43.6) | 4 (3,6) | 40.7 (29.7,58.9) | 46.5 (-1.6,111.4) |
| Niue | 1 (1,1) | 29.9 (23.1,40.3) | 1 (1,2) | 56.1 (33.1,86.2) | 87.8 (1.5,193.4) |
| Northern Mariana Islands | 6 (5,8) | 37.3 (30.1,47.4) | 25 (20,30) | 57.6 (47.3,68.4) | 54.7 (15.4,102) |
| Palau | 3 (2,4) | 29.5 (21.1,39.3) | 9 (6,12) | 50.2 (36.2,66.3) | 70 (5.7,162) |
| Papua New Guinea | 220 (145,312) | 11.3 (7.9,15.9) | 728 (561,945) | 13.6 (10.4,19) | 20.8 (-22.4,94.2) |
| Samoa | 24 (17,33) | 31 (22.2,41) | 58 (44,75) | 43.5 (33,55.5) | 40.2 (-4,111.8) |
| Solomon Islands | 31 (13,50) | 22.1 (11.6,34.6) | 90 (69,116) | 25 (20.1,31.5) | 13.1 (-30,124.8) |
| Tokelau | 0 (0,1) | 24.6 (17.1,42.5) | 1 (0,1) | 37.3 (27,54.7) | 51.7 (2.4,123.7) |
| Tonga | 7 (5,10) | 33.5 (24.6,48.1) | 31 (24,39) | 73.8 (57.4,91.7) | 120.1 (35,244.4) |
| Tuvalu | 2 (1,3) | 27.8 (20.8,43.6) | 4 (3,6) | 40.7 (29.7,58.9) | 46.5 (-1.6,111.4) |
| Vanuatu | 13 (8,22) | 22 (14.3,38.7) | 55 (40,85) | 34.3 (24.7,52.6) | 55.9 (4.7,130.7) |
| **South Asia** | 79714 (68086,89404) | 14 (12.1,15.9) | 226043 (192546,264155) | 16.5 (14,19.3) | 17.7 (-7.6,41.4) |
| Bangladesh | 9553 (6572,11510) | 14.9 (12.8,18.3) | 15175 (11740,20173) | 12.3 (9.5,16.2) | -17.6 (-36.9,6.2) |
| Bhutan | 42 (30,58) | 17 (12.4,24.2) | 120 (81,165) | 20.9 (14,28.5) | 22.8 (-14.3,68.8) |
| India | 58475 (50679,67536) | 13.1 (11.2,15.2) | 175637 (146072,208715) | 15.8 (13.1,18.7) | 21 (-10.1,47.6) |
| Nepal | 1249 (960,1663) | 13.5 (10.4,18.1) | 4048 (2947,5424) | 19.2 (14.1,25.1) | 42.5 (-12,100.6) |
| Pakistan | 10395 (8266,13167) | 18.8 (14.9,23.7) | 31063 (24330,39844) | 26.6 (21.4,33.9) | 41.8 (7.9,85.4) |
| **Southeast Asia** | 57881 (52199,66490) | 22.9 (20.6,26.7) | 170033 (148864,190537) | 28.5 (24.8,31.9) | 24.6 (2.4,43.3) |
| Cambodia | 1050 (857,1277) | 21.2 (17.5,25.7) | 2681 (2007,3484) | 23.5 (17.8,30.2) | 10.7 (-25,54.2) |
| Indonesia | 18490 (15846,22280) | 17.3 (14.9,21.8) | 51165 (41553,63408) | 23 (18.8,29) | 32.6 (2.9,60.8) |
| Lao People's Democratic Republic | 917 (677,1191) | 43.9 (33.2,56.9) | 1775 (1242,2408) | 40.9 (29.1,54.7) | -6.9 (-34.5,27.7) |
| Malaysia | 2075 (1795,2376) | 22.6 (19.4,25.9) | 7575 (6352,8581) | 29.2 (24.4,33) | 29.2 (0.5,52.4) |
| Maldives | 40 (34,51) | 48.4 (39.7,69.3) | 88 (71,105) | 28.9 (23.4,34.5) | -40.3 (-57.8,-21.8) |
| Mauritius | 302 (288,315) | 44.3 (42,46.4) | 1414 (1304,1494) | 80.1 (74.1,84.6) | 80.8 (68.7,92.9) |
| Myanmar | 6501 (4977,8336) | 26.6 (20.6,34.4) | 11244 (9001,14131) | 24.9 (20.3,31.1) | -6.1 (-33.4,31.9) |
| Philippines | 8626 (7839,9922) | 31.7 (28.9,35.6) | 32883 (27438,38113) | 41.7 (35,48.1) | 31.5 (8.5,54) |
| Seychelles | 17 (15,19) | 29.3 (25.5,33.4) | 50 (40,58) | 47.2 (37.9,55.4) | 61.2 (26.8,101.5) |
| Sri Lanka | 2614 (2219,3080) | 27.7 (23.3,32.8) | 5243 (3567,7155) | 21.2 (14.6,28.5) | -23.3 (-50.8,14.6) |
| Thailand | 7941 (6624,9995) | 24.3 (20.2,30.6) | 31734 (24197,39501) | 29.9 (22.8,37.1) | 23.2 (-13.4,63.9) |
| Timor-Leste | 76 (57,101) | 25.6 (19.7,35.6) | 238 (172,335) | 29.8 (21.8,41.4) | 16 (-19,61.6) |
| Viet Nam | 9150 (6657,11695) | 23.5 (17.1,30.1) | 23706 (16677,29759) | 27 (19.1,33.4) | 15.2 (-18.9,51) |
| **Southern Latin America** | 11256 (10698,11760) | 26 (24.6,27.2) | 21583 (19510,22986) | 23.8 (21.6,25.4) | -8.3 (-14.1,-3) |
| Uruguay | 598 (561,628) | 15.5 (14.5,16.2) | 1167 (1034,1270) | 17.9 (16.1,19.4) | 15.6 (6.1,25.2) |
| Argentina | 9185 (8697,9648) | 30.4 (28.6,31.9) | 15213 (13851,16266) | 26.3 (24,28.1) | -13.3 (-19,-7.6) |
| Chile | 1471 (1390,1543) | 16 (15,16.9) | 5202 (4582,5596) | 19.8 (17.5,21.3) | 23.8 (13.5,32.4) |
| **Southern Sub-Saharan Africa** | 5376 (4674,6619) | 20.9 (18,26.2) | 17365 (15612,19485) | 34.4 (31.1,38.3) | 65 (26.9,89.5) |
| Botswana | 109 (79,157) | 23.1 (17,32.1) | 348 (270,479) | 28.5 (23,39) | 23.3 (-9.5,70.9) |
| Eswatini | 100 (79,125) | 38.7 (30.5,48.3) | 274 (174,383) | 53.5 (36.5,71.6) | 38.4 (-3.2,91.9) |
| Lesotho | 153 (119,202) | 19.9 (15.3,26.5) | 445 (311,595) | 46.2 (32.8,59.4) | 132.6 (58.1,214.1) |
| Namibia | 119 (92,169) | 21.8 (16.7,31) | 313 (231,422) | 26.7 (20.3,35.2) | 22.3 (-8.7,61.9) |
| South Africa | 4020 (3508,4993) | 19.7 (16.8,25.1) | 13502 (12124,14916) | 33.2 (29.6,36.6) | 68.3 (27.7,94.4) |
| Zimbabwe | 874 (693,1148) | 26 (20.5,34.2) | 2483 (1893,3317) | 41.4 (31.8,53.6) | 59.3 (13.3,110.9) |
| **Tropical Latin America** | 15524 (14832,16076) | 17.9 (16.9,18.7) | 46925 (42508,49438) | 18.8 (17,19.9) | 5 (-0.5,9.1) |
| Brazil | 15168 (14505,15699) | 18 (16.9,18.8) | 45537 (41271,47987) | 18.7 (16.9,19.7) | 4 (-1.2,8.1) |
| Paraguay | 355 (301,418) | 16.1 (13.6,19.1) | 1389 (1036,1761) | 24.9 (18.7,31.5) | 54.4 (9.5,103.7) |
| **Western Europe** | 48662 (44430,50877) | 8.3 (7.5,8.7) | 133481 (109637,148166) | 10.7 (8.9,11.8) | 29 (18.4,37.6) |
| Andorra | 5 (4,7) | 12.1 (8.8,16.7) | 17 (12,23) | 9.3 (6.9,12.5) | -23.3 (-48.3,11.8) |
| Austria | 786 (724,834) | 6.6 (6.1,7) | 3411 (2801,3759) | 14.3 (11.9,15.6) | 117.1 (94.5,135) |
| Belgium | 1386 (1260,1478) | 8.8 (8,9.4) | 2816 (2274,3150) | 9 (7.5,10) | 2.1 (-7.4,10.6) |
| Cyprus | 188 (156,238) | 41.3 (34.1,51.5) | 343 (284,403) | 21.5 (18.1,25) | -47.8 (-58.9,-36.5) |
| Denmark | 413 (379,437) | 4.8 (4.5,5.1) | 1699 (1457,1893) | 12.1 (10.5,13.5) | 150.4 (127.7,173.3) |
| Finland | 214 (194,227) | 3 (2.7,3.2) | 818 (657,927) | 4.9 (4,5.5) | 61.4 (44.1,77) |
| France | 6888 (6157,7355) | 7.7 (6.9,8.3) | 15133 (12606,16843) | 7.3 (6.2,8.1) | -5.4 (-13.7,2.3) |
| Germany | 11051 (10128,11739) | 8.3 (7.6,8.8) | 39563 (31943,45262) | 15.4 (12.6,17.4) | 85 (64.7,105.6) |
| Greece | 2985 (2744,3174) | 21.3 (19.4,22.7) | 7053 (6077,7641) | 21.3 (18.7,23) | 0.1 (-6.4,6) |
| Iceland | 11 (10,12) | 3.6 (3.2,3.9) | 41 (33,46) | 5.8 (4.7,6.5) | 61.2 (44.6,80.1) |
| Ireland | 311 (288,330) | 8.2 (7.5,8.7) | 703 (587,792) | 8.2 (6.9,9.2) | -0.2 (-11.9,10.3) |
| Israel | 970 (893,1037) | 22.2 (20.2,23.8) | 2790 (2314,3075) | 19.7 (16.5,21.7) | -11.2 (-19.6,-4.7) |
| Italy | 7528 (6774,7922) | 8.8 (7.8,9.2) | 19086 (15314,21515) | 9.3 (7.6,10.5) | 6 (-3.6,15) |
| Luxembourg | 52 (49,55) | 10.3 (9.5,10.9) | 158 (130,184) | 12.6 (10.5,14.5) | 22.6 (6.4,40.3) |
| Malta | 46 (42,49) | 12.1 (10.9,12.9) | 141 (116,160) | 12.5 (10.4,14.1) | 3.1 (-9,15.6) |
| Monaco | 5 (4,6) | 6.1 (4.6,7.4) | 12 (9,14) | 9.6 (7.6,11.4) | 58.1 (24.1,109.6) |
| Netherlands | 1325 (1179,1417) | 6.6 (5.9,7.1) | 4435 (3715,4925) | 10.9 (9.2,12.1) | 63.8 (51.5,75.9) |
| Norway | 268 (241,282) | 3.5 (3.2,3.7) | 805 (673,878) | 6.5 (5.5,7) | 83.5 (71.4,92.4) |
| Portugal | 1597 (1486,1687) | 13 (12,13.8) | 4736 (3954,5301) | 14.5 (12.3,16.2) | 11.3 (-1.6,23.1) |
| San Marino | 2 (2,2) | 5.5 (4.5,6.6) | 4 (3,6) | 40.7 (29.7,58.9) | 46.5 (-1.6,111.4) |
| Spain | 6907 (6235,7340) | 13.3 (11.9,14.1) | 14802 (11854,16621) | 10.5 (8.6,11.8) | -21.2 (-29.2,-13.8) |
| Sweden | 636 (578,678) | 3.8 (3.5,4.1) | 2518 (2087,2834) | 8.9 (7.4,9.9) | 131.8 (106.5,154.6) |
| Switzerland | 808 (722,860) | 7.1 (6.4,7.6) | 2599 (2042,2973) | 10.2 (8.1,11.7) | 42.8 (26.7,60.9) |
| United Kingdom | 4239 (3908,4410) | 4.6 (4.2,4.8) | 9679 (8207,10541) | 6.2 (5.4,6.8) | 36 (28.4,42.2) |
| **Western Sub-Saharan Africa** | 28294 (24259,32687) | 33.6 (29,39.2) | 64796 (53512,76124) | 36.4 (31.1,42.8) | 8.2 (-9.3,24.9) |
| Benin | 782 (673,912) | 37.3 (32.3,44.7) | 1940 (1558,2414) | 39.4 (32.5,48.4) | 5.6 (-16.1,31.4) |
| Burkina Faso | 1555 (1281,1850) | 38.3 (31.8,45.8) | 3812 (3071,4633) | 43.7 (35.3,53) | 13.9 (-13.9,45.1) |
| Cabo Verde | 38 (32,44) | 15.4 (12.7,17.9) | 124 (84,155) | 28.9 (19.4,36) | 88 (26.6,142.9) |
| Cameroon | 2151 (1677,2797) | 52 (40,67.1) | 5928 (4179,8334) | 51.2 (37.1,71.1) | -1.6 (-24,32.5) |
| Chad | 751 (593,1013) | 26.4 (20.5,36.9) | 1811 (1330,2568) | 31.6 (23.4,45.6) | 19.7 (-6.2,53.1) |
| Gambia | 115 (90,143) | 33.7 (26.8,41.3) | 403 (305,513) | 43.2 (33.2,54.7) | 28.1 (-9.7,72.7) |
| Ghana | 1745 (1343,2490) | 29.5 (22.8,41.8) | 7076 (5563,8854) | 47.7 (37.9,59.3) | 61.5 (-0.9,121.8) |
| Guinea | 1055 (850,1342) | 32.3 (25.5,41.8) | 1874 (1414,2582) | 34.5 (25.9,47.8) | 6.8 (-19.3,41.1) |
| Guinea-Bissau | 201 (157,246) | 50.1 (40.3,60.7) | 316 (246,404) | 46.4 (37,58.9) | -7.5 (-28.5,22.2) |
| Liberia | 533 (427,673) | 46.4 (38,57.8) | 954 (706,1263) | 48.4 (36.3,63.3) | 4.3 (-26.5,42.5) |
| Mali | 1405 (1153,1734) | 38.3 (31.3,47.7) | 3001 (2407,3922) | 37.4 (30.3,48.1) | -2.3 (-22,24.5) |
| Mauritania | 394 (305,502) | 42.6 (33.2,54.3) | 774 (532,1095) | 40.4 (27.5,56.5) | -5.2 (-30.5,24.2) |
| Niger | 874 (706,1119) | 29.5 (23.9,38.7) | 2007 (1440,3038) | 27 (19.9,40.1) | -8.6 (-30.5,19.5) |
| Nigeria | 12895 (10282,15658) | 31 (25.1,37.6) | 25429 (19318,32599) | 31.3 (25,38.1) | 1 (-22,23.9) |
| Sao Tome and Principe | 34 (28,38) | 53.7 (45.3,60.8) | 69 (50,85) | 72.4 (51.4,86.9) | 34.8 (-5,65.3) |
| Senegal | 1300 (1084,1593) | 40.6 (34.1,50.3) | 3035 (2315,4175) | 43.5 (33.6,59.2) | 7.2 (-19.2,43.4) |
| Sierra Leone | 628 (495,784) | 30.1 (23.8,37.2) | 1048 (802,1392) | 29 (22.6,37.5) | -3.7 (-25,23) |
| Togo | 381 (314,467) | 31 (26.1,38) | 1136 (854,1520) | 34.5 (26.7,45.2) | 11.5 (-15.2,46.4) |
| CÃ ́te d'Ivoire | 1455 (1171,1787) | 38.9 (32,47.7) | 4060 (3151,5240) | 40.5 (33,51.2) | 4.1 (-17.2,32.9) |
