# Supplementary material for: Burden of chronic kidney disease and its attributable risk factors in 204 countries and territories, 1990–2021: Results from the Global Burden of Disease study 2021
Source: Medicine (Baltimore). 2026 Aug 7;105(32):e50111. doi: 10.1097/MD.0000000000050111 (PMC13456769; doi:10.1097/MD.0000000000050111)
Supplement: Supplementary file 5 [file medi-105-e50111-s007.docx]

**Table S5.** DALYs due to chronic kidney disease in 1990 and 2021 and the percentage change in the age-standardised rates (ASRs) per 100,000, by location.

|  | **1990** | | **2021** | | **Percentage change in the ASRs per 100,000** |
| --- | --- | --- | --- | --- | --- |
|  | **No (95% UI)** | **ASRs per 100,000 (95% UI)** | **No (95% UI)** | **ASRs per 100,000 (95% UI)** |  |
| **Global** | 20739895 (18843684,22588533) | 479.8 (439.2,523.8) | 44453684 (40840762,48508462) | 529.6 (486.2,577.4) | 10.4 (0.8,17.8) |
| **Andean Latin America** | 190986 (174177,211377) | 754 (688,838.7) | 524246 (434446,625013) | 872.4 (723.5,1037.9) | 15.7 (-4.2,41.2) |
| Bolivia (Plurinational State of) | 45375 (38779,56751) | 1123.7 (953.4,1408.5) | 121335 (92262,157620) | 1307.4 (1008.7,1688.7) | 16.4 (-13,59.1) |
| Ecuador | 40275 (37767,42623) | 619.3 (581.4,655.6) | 155833 (114778,216359) | 950.4 (705.5,1310.6) | 53.5 (14.7,108.7) |
| Peru | 105337 (92303,120681) | 713.7 (619.9,822.7) | 247079 (184433,311887) | 720.9 (537.4,910.6) | 1 (-25.5,30.4) |
| **Australasia** | 46405 (42217,50551) | 211.1 (192.4,229.6) | 113693 (101514,125301) | 216.6 (193.2,239.2) | 2.6 (-2.5,7.1) |
| Australia | 38617 (35222,42200) | 211.2 (193.1,230.1) | 93074 (82639,102916) | 207.1 (183.5,229) | -1.9 (-7.3,2.8) |
| New Zealand | 7788 (7047,8501) | 211.3 (190.8,230.6) | 20618 (18809,22453) | 263.6 (241.2,286.6) | 24.7 (17.4,32) |
| **Caribbean** | 161590 (149056,180712) | 565.1 (523.6,636.6) | 385286 (331558,454109) | 735.8 (631,867.9) | 30.2 (12.9,49.6) |
| Antigua and Barbuda | 436 (405,466) | 807.8 (751.9,862.9) | 1162 (1070,1242) | 1116.5 (1028.1,1190.1) | 38.2 (25,52.6) |
| Bahamas | 1314 (1209,1413) | 710.7 (653.6,766.6) | 4315 (3520,5314) | 1035.6 (849,1270.9) | 45.7 (17.8,83.4) |
| Barbados | 1555 (1455,1646) | 564.8 (528.5,598.1) | 3422 (2743,4181) | 732.6 (589.2,903) | 29.7 (4.1,61.2) |
| Belize | 848 (796,903) | 718.8 (674.5,763.4) | 4198 (3725,4677) | 1236.1 (1098.8,1377.9) | 72 (50.6,93.2) |
| Bermuda | 264 (244,284) | 421.4 (389.5,452.8) | 482 (414,570) | 395.4 (339.7,465.5) | -6.2 (-19.2,10) |
| Cuba | 29762 (27495,32222) | 285.6 (263.5,308.7) | 79000 (69453,88900) | 436.6 (383.8,494.2) | 52.9 (33.8,72.1) |
| Dominica | 502 (447,555) | 836.1 (744.6,925.4) | 1029 (819,1263) | 1295.5 (1035.6,1585.1) | 55 (24.1,90.6) |
| Dominican Republic | 25415 (22411,29374) | 515.4 (449.4,606.1) | 73332 (54273,90211) | 701.5 (518.5,864.8) | 36.1 (-3.5,74.4) |
| Grenada | 742 (678,812) | 1044.7 (958.2,1144) | 1684 (1467,1904) | 1487.2 (1300.8,1673.6) | 42.4 (22.4,63) |
| Guyana | 4232 (3811,4631) | 883.2 (800.5,971.4) | 10889 (8428,13860) | 1574.8 (1227.3,1986.4) | 78.3 (34.2,126.8) |
| Haiti | 40217 (31355,58452) | 909.5 (697.6,1415.1) | 82512 (50633,163742) | 916.4 (561.1,1807.6) | 0.8 (-33.2,39.2) |
| Jamaica | 11644 (10948,12362) | 617.4 (578.5,656.5) | 25773 (19982,32671) | 831.3 (642.4,1055.4) | 34.6 (4.7,73.8) |
| Puerto Rico | 28212 (26770,29738) | 793 (753.4,835.2) | 51201 (43010,59665) | 821.9 (692.5,956.4) | 3.6 (-12.6,20.8) |
| Saint Kitts and Nevis | 420 (392,454) | 1164.4 (1090.2,1254.5) | 914 (747,1082) | 1350.4 (1133.2,1575.8) | 16 (-5.1,35.5) |
| Saint Lucia | 885 (837,936) | 955 (904,1009.7) | 2376 (1996,2807) | 1041.6 (875.8,1230) | 9.1 (-9.4,29.4) |
| Saint Vincent and the Grenadines | 569 (534,610) | 737.2 (689.7,789) | 1457 (1294,1663) | 1083.5 (962.5,1234.4) | 47 (28.6,68.4) |
| Suriname | 2433 (2055,2721) | 831.3 (717.6,937.4) | 7429 (5840,9298) | 1181.2 (931.8,1473) | 42.1 (7.8,86) |
| Trinidad and Tobago | 6161 (5850,6493) | 681.8 (647.7,716.4) | 20236 (15316,25658) | 1101.3 (836.6,1394.7) | 61.5 (22.8,109.2) |
| United States Virgin Islands | 510 (432,606) | 563.5 (478.3,667.3) | 835 (611,1068) | 599.2 (452.3,758) | 6.3 (-24.9,42.4) |
| **Central Asia** | 176292 (152244,202201) | 326.1 (278.5,378.8) | 427633 (377524,490696) | 493.2 (436,565.5) | 51.3 (37.2,68) |
| Armenia | 4757 (3535,5893) | 168.2 (126.2,208.6) | 15239 (12675,18395) | 380.2 (316.2,457.4) | 126 (87.7,189.3) |
| Azerbaijan | 22175 (18576,26606) | 374.1 (313.5,447.8) | 47430 (37765,57959) | 451.9 (364.8,546.7) | 20.8 (-1.3,51) |
| Georgia | 15560 (12706,18459) | 264.2 (216.6,310.6) | 24983 (20472,30628) | 478.5 (394.3,587.7) | 81.1 (50,121.2) |
| Kazakhstan | 49636 (42866,56925) | 351.5 (300,403.8) | 80323 (68213,94683) | 448.3 (381.6,524.2) | 27.6 (10.2,46.5) |
| Kyrgyzstan | 12369 (10585,14240) | 346.9 (294.6,401.8) | 25313 (21875,29466) | 448.3 (386.9,523.7) | 29.2 (11.4,50.9) |
| Mongolia | 8119 (6668,9950) | 603.2 (498.7,719.3) | 13518 (11300,16158) | 508.1 (425.4,602.4) | -15.8 (-32.4,1.8) |
| Tajikistan | 6728 (5319,8239) | 198.2 (154.9,245.3) | 15292 (12007,19273) | 217.6 (169.3,274.6) | 9.8 (-3.5,28.7) |
| Turkmenistan | 12416 (11128,13743) | 475.9 (423.2,536.5) | 33906 (27087,41471) | 730.9 (586.8,893.3) | 53.6 (24,92.9) |
| Uzbekistan | 44533 (35798,54486) | 315.3 (247.4,392.7) | 171630 (147004,200979) | 580.8 (501.4,677.5) | 84.2 (51.6,124.4) |
| **Central Europe** | 469644 (436516,504841) | 342.8 (318.7,368.5) | 539982 (472193,612672) | 266.9 (233.4,306.2) | -22.1 (-28,-14.7) |
| Albania | 9416 (8198,10999) | 378.2 (328.6,441.4) | 11183 (9088,13561) | 303.9 (249.2,366.3) | -19.6 (-34.9,-1) |
| Bosnia and Herzegovina | 13424 (11787,15111) | 326.9 (287.9,366.2) | 17500 (14014,21236) | 311.2 (253.2,378.5) | -4.8 (-23.9,18.3) |
| Bulgaria | 27816 (24930,30650) | 274.1 (246.8,301.4) | 59631 (50184,71490) | 487.9 (408.3,590.7) | 78 (47.9,114.4) |
| Croatia | 15608 (14199,16944) | 284.1 (259,307) | 26558 (22898,31103) | 308.8 (265.4,364.9) | 8.7 (-5.1,28.4) |
| Czechia | 35113 (31942,38431) | 277 (253,303.2) | 39158 (33376,45630) | 197.7 (167.8,233.1) | -28.6 (-37.2,-18.1) |
| Hungary | 30171 (27070,33010) | 235.3 (211.2,256.8) | 46215 (39960,53280) | 249.3 (214.4,289) | 6 (-5.6,19.4) |
| Montenegro | 2342 (1990,2734) | 382.9 (325.9,447.3) | 3844 (3139,4690) | 440.5 (361.7,537.9) | 15.1 (-9.5,46.1) |
| North Macedonia | 6763 (5934,7869) | 365.5 (320.3,423) | 10889 (8783,13799) | 373.5 (303.8,461.8) | 2.2 (-16.1,24.5) |
| Poland | 166060 (154655,176751) | 405.7 (378.1,432.2) | 136197 (117984,158787) | 206.2 (177.9,241.2) | -49.2 (-53.7,-43) |
| Romania | 87976 (81527,94928) | 348.6 (324,375.7) | 92610 (78865,107339) | 278 (236.9,323.9) | -20.3 (-30.3,-8.8) |
| Serbia | 42655 (36005,50866) | 434.2 (367.7,519.7) | 58087 (48712,67910) | 374.3 (316.5,434.5) | -13.8 (-31.9,6.5) |
| Slovakia | 19699 (17271,22267) | 344.2 (302.5,388.6) | 22738 (19458,26211) | 265.3 (228.2,305.9) | -22.9 (-34.2,-10.2) |
| Slovenia | 5091 (4580,5635) | 220.2 (197.7,243.3) | 7512 (6152,9249) | 175.2 (142.3,217.9) | -20.4 (-31,-6.5) |
| **Central Latin America** | 779859 (736752,822191) | 767.9 (724.2,811.6) | 2993750 (2691663,3371708) | 1171.1 (1054.8,1316.3) | 52.5 (37.6,68.7) |
| Colombia | 122275 (114363,131312) | 573.8 (533.1,613.7) | 233106 (198873,269966) | 424.4 (362.5,491.3) | -26 (-35.5,-15.5) |
| Costa Rica | 9157 (8313,10118) | 468.5 (425.5,515.9) | 41809 (37382,46132) | 764.5 (683.8,843) | 63.2 (46.5,82.9) |
| El Salvador | 28536 (25800,38102) | 784.3 (704.6,1063.6) | 118020 (88527,144949) | 1904 (1419.6,2342.5) | 142.8 (50,214.4) |
| Guatemala | 44304 (42312,46649) | 920.2 (874.3,970.6) | 167359 (143276,194780) | 1387.5 (1182.2,1617.2) | 50.8 (26.4,75.7) |
| Honduras | 11630 (10042,13654) | 444.7 (379.4,536.2) | 46141 (35816,59163) | 682.7 (536.6,871) | 53.5 (11.7,99.6) |
| Mexico | 474218 (450937,498550) | 932.2 (884.9,983.3) | 1940953 (1680240,2260842) | 1489.7 (1295.2,1728) | 59.8 (40.5,82.9) |
| Nicaragua | 20446 (18371,26070) | 922.3 (832.9,1189.3) | 86099 (68224,102148) | 1596.8 (1282.9,1885.7) | 73.1 (21.4,111.9) |
| Panama | 7457 (6800,8224) | 442 (401.2,486.7) | 36062 (29067,42682) | 814.7 (656,963.9) | 84.3 (51.1,118.6) |
| Venezuela (Bolivarian Republic of) | 61836 (57241,66347) | 527.6 (484.6,568) | 324201 (249903,407035) | 1087 (843.3,1362.7) | 106 (59.7,161.4) |
| **Central Sub-Saharan Africa** | 349929 (293393,410516) | 1158.7 (983.4,1367) | 783700 (622595,996532) | 1124.7 (899,1436.1) | -2.9 (-23.8,24.9) |
| Angola | 60560 (47304,74043) | 1068.6 (834.9,1327) | 158051 (120480,207619) | 1011.8 (761.7,1315) | -5.3 (-29.3,26.7) |
| Central African Republic | 20433 (16924,24299) | 1327.3 (1106,1592.6) | 39107 (28354,53349) | 1303.3 (972.4,1758.2) | -1.8 (-24,27.7) |
| Congo | 19457 (15403,23362) | 1483.5 (1157.4,1769.5) | 44198 (31384,57320) | 1365.6 (964.3,1712.3) | -7.9 (-27.7,15.6) |
| Democratic Republic of the Congo | 238276 (194665,290653) | 1138.9 (935.7,1393.9) | 513717 (387253,677593) | 1112.3 (844.2,1452.8) | -2.3 (-28.4,35.2) |
| Equatorial Guinea | 3014 (2404,3713) | 1226.6 (976.6,1504.5) | 9444 (6004,13862) | 1323.1 (834.3,1860.8) | 7.9 (-31.5,57.5) |
| Gabon | 8190 (6669,9730) | 1304.8 (1072.4,1558) | 19184 (11205,25666) | 1670.8 (951.9,2188.5) | 28.1 (-15.3,68.2) |
| **East Asia** | 4372608 (3843678,4996867) | 461.3 (406.8,529.1) | 6486167 (5538060,7597380) | 322.4 (275.4,377.3) | -30.1 (-41.9,-18.5) |
| China | 4195631 (3673738,4813077) | 457.7 (402.1,526.9) | 6127923 (5184384,7208227) | 315.3 (266.6,371.5) | -31.1 (-43.2,-18.7) |
| Democratic People's Republic of Korea | 83478 (63914,105819) | 483.4 (373.1,605.9) | 153167 (124247,191899) | 479.2 (390.6,602.6) | -0.9 (-25.2,30.8) |
| Taiwan (Province of China) | 93499 (86835,100493) | 604.5 (560.6,649.3) | 205078 (181400,224760) | 502.7 (447,550.5) | -16.8 (-22.7,-11.2) |
| **Eastern Europe** | 521705 (463250,581791) | 207.4 (184.7,230.9) | 642467 (562943,726952) | 204.7 (180.4,232.1) | -1.3 (-7.9,7.1) |
| Belarus | 13038 (10184,15922) | 109.6 (86.1,132.8) | 21603 (17974,26090) | 149.8 (125.8,179.8) | 36.7 (22.2,53.3) |
| Estonia | 5689 (5107,6257) | 315.4 (284.6,344.8) | 11443 (9973,13008) | 455.8 (396.1,518.5) | 44.5 (28.4,63.9) |
| Latvia | 5859 (5024,6735) | 184.2 (159.9,210.5) | 9429 (7832,11172) | 271.3 (225.8,322.4) | 47.2 (28.9,72.3) |
| Lithuania | 7168 (6069,8288) | 172.3 (147.2,198) | 11654 (9803,13772) | 237.6 (201.7,283.9) | 37.9 (20.8,61.1) |
| Republic of Moldova | 7738 (6388,9225) | 184.4 (151.2,219.8) | 12608 (10418,14983) | 233.8 (193.3,279.2) | 26.7 (15.8,40.8) |
| Russian Federation | 428507 (391751,466416) | 259.2 (236.9,282) | 464004 (409800,529294) | 213.6 (189.1,243.6) | -17.6 (-23.5,- 9.3) |
| Ukraine | 53706 (38096,69878) | 84.8 (60.7,108.7) | 111725 (89366,135575) | 171.8 (138.9,207.4) | 102.6 (68.3,150.4) |
| **Eastern Sub-Saharan Africa** | 1117669 (975468,1248904) | 1095.2 (965.9,1255) | 2047629 (1792019,2374164) | 948.4 (838.7,1090.4) | -13.4 (-23,-0.9) |
| Burundi | 31943 (25440,39942) | 1032.6 (815.3,1304.7) | 52752 (39451,75471) | 837.3 (623.9,1184.7) | -18.9 (-39.5,10.3) |
| Comoros | 2494 (1867,3109) | 984.8 (763.6,1217) | 5242 (3955,6654) | 990.7 (748.1,1260.4) | 0.6 (-26.1,38.2) |
| Djibouti | 1395 (1048,1830) | 727.5 (561.1,939.8) | 6952 (5050,9547) | 959.1 (723.5,1275.5) | 31.8 (-9.9,84.5) |
| Eritrea | 15112 (11352,20868) | 900.2 (680.9,1231.1) | 32532 (22413,51729) | 930.2 (649.1,1463.5) | 3.3 (-18.9,35) |
| Ethiopia | 466721 (355203,540047) | 1765.7 (1400.4,2039.4) | 524752 (440481,620029) | 966 (806.9,1140.3) | -45.3 (-55.5,-31.9) |
| Kenya | 66436 (54525,94453) | 608.9 (488.9,921.4) | 230896 (188910,304540) | 863.7 (701.7,1139.4) | 41.8 (12,79.5) |
| Madagascar | 47382 (38780,60549) | 702.8 (568.5,945.4) | 100664 (76256,129965) | 678.7 (508.2,884.3) | -3.4 (-28.2,25.1) |
| Malawi | 56194 (46192,66313) | 1003.2 (830,1214.1) | 110135 (88010,133712) | 1114.9 (915.1,1338) | 11.1 (-13.4,38.5) |
| Mozambique | 59057 (48400,73912) | 681.4 (559,901.3) | 141035 (107847,186077) | 882.2 (684.6,1181.5) | 29.5 (-6.2,73.3) |
| Rwanda | 48445 (39539,56316) | 1255.6 (1043.6,1472.4) | 65862 (48112,87152) | 891.8 (663.5,1176.6) | -29 (-46.8,-5.2) |
| Somalia | 42592 (31540,57931) | 1182.7 (867,1615.4) | 109717 (77473,161379) | 1218.6 (853.7,1741) | 3 (-22,37.1) |
| South Sudan | 33407 (25693,43506) | 1014.3 (768.8,1354.6) | 69366 (50852,90453) | 1345 (1002.1,1742.3) | 32.6 (-11.3,83.8) |
| Uganda | 65235 (50387,82208) | 764.5 (582,980.3) | 183294 (144104,239789) | 919.2 (725.5,1193.4) | 20.2 (-12.3,64.7) |
| United Republic of Tanzania | 131082 (109800,160936) | 903.1 (755.1,1140.9) | 288849 (234235,364048) | 899.3 (731.7,1124.4) | -0.4 (-24.7,32.3) |
| Zambia | 49375 (39792,57931) | 1179.3 (995.9,1379.6) | 123798 (84564,188445) | 1289.7 (932.6,1817.4) | 9.4 (-22.8,69.5) |
| **High-income Asia Pacific** | 599581 (550356,643830) | 315.7 (289,339.2) | 1146613 (990343,1268312) | 235.6 (206,259.6) | -25.4 (-29.1,-22.3) |
| Brunei Darussalam | 1038 (881,1248) | 843.6 (710.9,1036.2) | 2607 (2282,2940) | 776.6 (676.5,884.2) | -7.9 (-25.2,11.5) |
| Japan | 460213 (416011,499612) | 290.4 (262.5,315) | 923022 (792680,1028099) | 239.9 (209.7,266.1) | -17.4 (-20.6,-14.7) |
| Republic of Korea | 130194 (117908,142679) | 429.6 (388.7,470.4) | 200278 (171976,226706) | 225.6 (193.7,255.8) | -47.5 (-54,-40.6) |
| Singapore | 8136 (7624,8708) | 355.8 (333.3,379.5) | 20705 (18435,23087) | 251.9 (223.9,280.3) | -29.2 (-34.7,-23.6) |
| **High-income North America** | 895097 (798640,986360) | 266.6 (238.3,293.2) | 3120983 (2864207,3351576) | 508.8 (467,545.4) | 90.8 (82.4,100) |
| Canada | 55398 (49172,61889) | 178.4 (158.9,198.9) | 150955 (134749,165748) | 224.7 (200.2,246) | 26 (17.7,35.5) |
| Greenland | 117 (102,135) | 335.7 (294.9,381.6) | 216 (178,257) | 352.2 (285.9,418.3) | 4.9 (-14.1,26) |
| United States of America | 839563 (749127,924830) | 276.1 (246.7,303.3) | 2969764 (2728422,3188334) | 543.3 (499.5,582.6) | 96.8 (87.8,106.5) |
| **North Africa and Middle East** | 1493457 (1272351,1963220) | 759.9 (642.7,1059.2) | 3925988 (3427227,4413622) | 846.6 (747.2,948) | 11.4 (-23.9,35.5) |
| Afghanistan | 94773 (70923,137292) | 1274.3 (956,1918.6) | 173983 (108001,286182) | 1343.2 (830.9,2358) | 5.4 (-36,59.1) |
| Algeria | 79125 (60711,119848) | 593.9 (442.4,962.7) | 241373 (197533,295747) | 724.2 (595.9,868.8) | 21.9 (-26.4,70.7) |
| Bahrain | 1611 (1272,2077) | 856.9 (670.3,1110.3) | 7698 (6382,9087) | 963.5 (793.3,1153.7) | 12.4 (-25.3,63.1) |
| Egypt | 344984 (287412,482687) | 1140.6 (932.6,1686.7) | 933318 (767956,1131670) | 1501.7 (1248.8,1804.3) | 31.7 (-17.2,73.6) |
| Iran (Islamic Republic of) | 139435 (116402,174468) | 463.6 (372.3,595.6) | 356124 (310077,393518) | 467.5 (406.8,515.1) | 0.8 (-27.7,21) |
| Iraq | 94335 (75090,120601) | 950.3 (757.7,1205.4) | 232396 (157615,296780) | 940.8 (660.6,1195) | -1 (-35.9,37.9) |
| Jordan | 12490 (10264,15613) | 749.9 (617.2,949.5) | 54300 (44489,67799) | 710.1 (581.4,884.9) | -5.3 (-33.3,24.9) |
| Kuwait | 5748 (5276,6312) | 716.8 (647.3,792.7) | 11585 (9417,13665) | 360.7 (286.1,427.9) | -49.7 (-58.7,-39.8) |
| Lebanon | 16012 (12337,21059) | 730.5 (569,955) | 39230 (33152,46228) | 630.3 (532.3,739.4) | -13.7 (-35.7,16.5) |
| Libya | 15078 (11908,19609) | 675.2 (524.4,903.5) | 58512 (37581,76498) | 1067 (712.7,1379.3) | 58 (-14.1,142.9) |
| Morocco | 94039 (70697,146372) | 572.7 (420.8,963.5) | 289479 (223424,366427) | 861.5 (664.2,1088.9) | 50.4 (-7.3,107) |
| Oman | 4965 (3836,6906) | 578.2 (442.1,815.4) | 19110 (15607,23751) | 879 (718.9,1088.5) | 52 (4.5,109) |
| Palestine | 9946 (7801,12853) | 979.4 (756.4,1283.1) | 23468 (20331,27681) | 885.7 (758.8,1049) | -9.6 (-34.1,21.8) |
| Qatar | 1101 (745,1589) | 821.9 (512.1,1248.9) | 7653 (6095,9741) | 757 (614.5,939.6) | -7.9 (-39.6,52.4) |
| Saudi Arabia | 80180 (61535,108291) | 1089.8 (832.6,1468.2) | 416093 (307761,529386) | 1762.8 (1351.1,2169.6) | 61.8 (-6.6,127.4) |
| Sudan | 76183 (58482,110063) | 624.9 (468.4,1022.6) | 180786 (136192,237944) | 770.7 (590,1012.6) | 23.3 (-24.6,83) |
| Syrian Arab Republic | 68209 (55197,84454) | 968.5 (772.3,1242.8) | 126862 (93695,168253) | 1020.6 (760,1328.9) | 5.4 (-31.5,50.9) |
| Tunisia | 28625 (23139,40343) | 534.6 (427.2,765.1) | 85581 (62643,113057) | 677.1 (500.9,889.4) | 26.7 (-24.8,83) |
| Turkey | 288241 (239747,364731) | 749.2 (624.5,967.7) | 552195 (453860,666416) | 619.4 (509.5,750) | -17.3 (-41.3,3.8) |
| United Arab Emirates | 3892 (2982,5153) | 586.6 (434.3,801.6) | 29680 (21617,37421) | 782.9 (522.7,997.8) | 33.5 (-14.7,75.7) |
| Yemen | 33670 (24098,53572) | 521.1 (367.1,927.4) | 82899 (60825,131865) | 510 (372.6,812.5) | -2.1 (-30,36.2) |
| **Oceania** | 22680 (16686,29125) | 583.7 (445.8,742) | 65815 (55558,77796) | 699 (597.9,821.3) | 19.8 (-9.5,60.4) |
| American Samoa | 260 (201,353) | 953.2 (740.8,1294.2) | 938 (740,1163) | 1950.9 (1545.9,2408.4) | 104.7 (32.9,208.9) |
| Cook Islands | 71 (59,89) | 535 (446.1,669.1) | 151 (120,180) | 631.4 (503.8,753) | 18 (-17,58.8) |
| Fiji | 3880 (2939,5283) | 849.7 (647.6,1147.1) | 10463 (7966,13388) | 1313 (1004.2,1661.1) | 54.5 (-3.9,137.7) |
| Guam | 535 (470,650) | 626.6 (552.2,754.4) | 1682 (1452,1875) | 846.8 (733.4,946.5) | 35.2 (8.8,58.9) |
| Kiribati | 460 (380,548) | 970 (797.7,1154.4) | 1109 (759,1633) | 1285.5 (892.4,1861.8) | 32.5 (-15.4,101) |
| Marshall Islands | 212 (144,351) | 990.7 (669.1,1662.6) | 669 (256,1739) | 1587.8 (613.1,4196.4) | 60.3 (-20.1,159) |
| Micronesia (Federated States of) | 595 (425,860) | 1017.5 (724.1,1495.1) | 1337 (979,1830) | 1629.5 (1205.4,2229.3) | 60.1 (0.9,147.2) |
| Nauru | 70 (47,100) | 1204.2 (807.1,1774.9) | 127 (95,167) | 1785 (1311.6,2476.4) | 48.2 (1.4,122.6) |
| Niue | 19 (16,25) | 880.2 (706,1130.8) | 32 (20,47) | 1612.3 (1011.2,2371.9) | 83.2 (5.4,174.2) |
| Northern Mariana Islands | 259 (200,334) | 1022.8 (833.2,1275.7) | 773 (644,914) | 1475 (1249.1,1715.8) | 44.2 (11,86.5) |
| Palau | 97 (72,125) | 880.1 (657.4,1119.2) | 293 (214,385) | 1382.2 (1017.8,1796) | 57.1 (4.2,133.1) |
| Papua New Guinea | 11577 (8052,15381) | 451.5 (332.1,589) | 36547 (30525,44537) | 515.1 (430.9,644.5) | 14.1 (-19.2,62) |
| Samoa | 868 (651,1147) | 891.3 (674.9,1163.8) | 1888 (1466,2394) | 1214.4 (948,1524.9) | 36.2 (-2.3,96.4) |
| Solomon Islands | 1408 (669,2226) | 774.7 (410.4,1191.8) | 4011 (3105,5040) | 870.9 (699.1,1073.3) | 12.4 (-25.8,105) |
| Tokelau | 10 (7,16) | 752.8 (553,1180.9) | 16 (12,22) | 1136.2 (878.9,1548.4) | 50.9 (4.9,111.2) |
| Tonga | 294 (223,388) | 481.5 (368.9,628.6) | 535 (402,689) | 639.2 (479.5,818.8) | 32.7 (0.1,77.2) |
| Tuvalu | 65 (51,93) | 888.1 (693.7,1286.5) | 127 (95,175) | 1178.5 (885,1617.2) | 32.7 (-6.3,81.9) |
| Vanuatu | 551 (376,866) | 696.8 (485.1,1113.2) | 2161 (1586,3205) | 1042 (776.4,1542.2) | 49.5 (7,111.1) |
| **South Asia** | 3674401 (3157460,4115106) | 509.8 (449.6,573.3) | 8443339 (7372315,9681828) | 540.6 (473.8,620.4) | 6 (-10.1,23.7) |
| Bangladesh | 551460 (306452,697970) | 604.3 (447.8,702) | 577364 (481335,715354) | 408 (343.9,506) | -32.5 (-44.8,-7.4) |
| Bhutan | 1915 (1398,2531) | 571.2 (433,749) | 3865 (2821,5112) | 600.1 (438.8,786.1) | 5.1 (-20.8,40.8) |
| India | 2650925 (2335452,3030444) | 481.6 (422.5,547.6) | 6487629 (5592187,7501487) | 521.8 (451.8,601.8) | 8.4 (-12,25.8) |
| Nepal | 65171 (52099,79837) | 548.6 (437.4,676.3) | 160662 (126887,204193) | 652.7 (513.5,826) | 19 (-10.6,50.1) |
| Pakistan | 404931 (332986,499986) | 590.5 (491.7,724.2) | 1213820 (972621,1508801) | 795.1 (645.5,990.2) | 34.6 (5.2,67.2) |
| **Southeast Asia** | 2408834 (2133712,2690991) | 751.9 (672.3,850.8) | 5703263 (5028657,6329025) | 846.3 (753.1,940.5) | 12.5 (-2.1,26.6) |
| Cambodia | 48373 (39570,58172) | 757.4 (633.3,895.9) | 101003 (77788,132516) | 733.6 (574.4,943.9) | -3.1 (-30.8,31.3) |
| Indonesia | 875942 (750362,1005150) | 664 (576,770.3) | 1990496 (1655069,2360240) | 760.4 (644.6,906.7) | 14.5 (-5.7,36.1) |
| Lao People's Democratic Republic | 36532 (27933,46903) | 1380.5 (1067.1,1764.2) | 63295 (45478,85994) | 1174.6 (854.1,1578.5) | -14.9 (-38,14.9) |
| Malaysia | 77325 (68494,86547) | 713.4 (633.7,796.6) | 238095 (207099,268811) | 817.7 (710.6,922.9) | 14.6 (-5.1,30.5) |
| Maldives | 1558 (1280,1852) | 1347.6 (1152.4,1701.1) | 2804 (2338,3335) | 719.8 (606.7,852.7) | -46.6 (-58.7,-34) |
| Mauritius | 10096 (9532,10683) | 1253.6 (1184.9,1323.6) | 38582 (35823,40737) | 2196.1 (2043.1,2318.9) | 75.2 (61.9,85.9) |
| Myanmar | 292701 (227943,364529) | 964.3 (759.6,1204.7) | 410628 (337703,500939) | 799.6 (662.6,966.4) | -17.1 (-37.1,11.7) |
| Philippines | 354857 (323905,395725) | 919.3 (837.5,1037.4) | 1136959 (958347,1307840) | 1232.7 (1049.6,1414.5) | 34.1 (10.4,56) |
| Seychelles | 522 (466,589) | 886.7 (790.4,1000.8) | 1424 (1193,1643) | 1226.4 (1031.4,1409.8) | 38.3 (11.7,67.3) |
| Sri Lanka | 91811 (79288,106066) | 769 (660,894.4) | 161739 (117194,209551) | 628.1 (460.8,813.2) | -18.3 (-42.4,10.8) |
| Thailand | 306169 (262369,368081) | 746.2 (641,897.8) | 865932 (699035,1045232) | 859.1 (698.5,1033.1) | 15.1 (-13.7,46) |
| Timor-Leste | 3510 (2766,4402) | 805.7 (634,1037.6) | 8029 (6032,10955) | 852 (645.3,1147.7) | 5.7 (-23.7,44.4) |
| Viet Nam | 305955 (223778,380082) | 671.5 (506.4,832.4) | 676321 (501845,849831) | 685.9 (514.2,847.4) | 2.1 (-24.4,32.9) |
| **Southern Latin America** | 281092 (266405,294525) | 612.7 (580.9,641.7) | 441138 (411345,467913) | 515.2 (481.8,546.8) | -15.9 (-20.3,-11.5) |
| Uruguay | 13717 (12910,14498) | 364.8 (343.3,385.1) | 21441 (19615,23134) | 387.1 (357.2,417.5) | 6.1 (-0.9,14.3) |
| Argentina | 223942 (212662,235443) | 705.3 (669.3,740.3) | 315536 (295283,335059) | 573.2 (537.7,608.5) | -18.7 (-23.6,-13.7) |
| Chile | 43420 (40600,46183) | 413.7 (384.8,440.4) | 104137 (95786,111766) | 415.1 (383.4,445.7) | 0.4 (-5.9,5.8) |
| **Southern Sub-Saharan Africa** | 202978 (179955,234849) | 623.9 (548.6,742.1) | 551466 (494226,622064) | 896 (807.3,997) | 43.6 (19.2,61.7) |
| Botswana | 4159 (3143,5808) | 645.6 (493,883.8) | 11766 (9282,16126) | 734.5 (596.1,978.2) | 13.8 (-15.4,51.2) |
| Eswatini | 3616 (2910,4479) | 1010.5 (813.5,1237.9) | 9663 (6172,13306) | 1431 (945,1925) | 41.6 (-0.4,94.7) |
| Lesotho | 5121 (4128,6439) | 560.6 (450.9,707.1) | 14547 (10549,19456) | 1221.5 (901,1605.1) | 117.9 (58.2,186.5) |
| Namibia | 4420 (3529,5944) | 613.6 (493.2,821.7) | 10401 (7883,13859) | 695.8 (539.9,902.1) | 13.4 (-13.5,46) |
| South Africa | 155415 (136560,179958) | 616.7 (540.4,733.3) | 415624 (377739,462537) | 862.3 (784.9,953.7) | 39.8 (19.3,55.8) |
| Zimbabwe | 30248 (24565,39272) | 664 (538,847) | 89465 (68552,116945) | 1084.3 (848.3,1401.9) | 63.3 (18.5,112) |
| **Tropical Latin America** | 594892 (560161,631009) | 562.7 (527.9,598.7) | 1311915 (1220675,1401164) | 517 (480.6,552.1) | -8.1 (-12,-4.7) |
| Brazil | 582735 (547944,618485) | 565.2 (529.7,600.8) | 1273112 (1182007,1361964) | 513.8 (477,550.1) | -9.1 (-12.8,-5.9) |
| Paraguay | 12158 (10659,13822) | 465.6 (403.4,536) | 38803 (31124,48290) | 643.4 (516,802.8) | 38.2 (4.7,79.1) |
| **Western Europe** | 1267566 (1125408,1409086) | 234.1 (206.8,260.1) | 2361342 (2067098,2638163) | 241.7 (209.8,271.1) | 3.2 (-1.7,8.5) |
| Andorra | 148 (115,187) | 286.9 (224.8,360.9) | 357 (285,435) | 229.2 (181.7,279.9) | -20.1 (-39.3,2.4) |
| Austria | 23090 (20228,25966) | 213.4 (187,238.5) | 56356 (49530,62593) | 288.2 (253.5,323.9) | 35.1 (25.2,44.5) |
| Belgium | 36155 (31529,40640) | 248.1 (215.3,279.2) | 55840 (47926,63208) | 234 (201.3,266.8) | -5.7 (-11.2,0.1) |
| Cyprus | 3922 (3338,4773) | 660.4 (562,797.9) | 6772 (5876,7764) | 377.8 (329.3,432.4) | -42.8 (-52.5,-33.1) |
| Denmark | 13497 (11412,15540) | 177.6 (150.6,205.1) | 31945 (28185,35763) | 266.7 (235,300.9) | 50.2 (38,65) |
| Finland | 8482 (7019,9973) | 126 (103.9,148) | 18425 (15613,21274) | 145.1 (120.5,168.5) | 15.2 (7.2,22.9) |
| France | 146507 (130176,162496) | 180.8 (160.5,199.7) | 266719 (232254,297859) | 174 (151.5,196.1) | -3.7 (-9.2,2) |
| Germany | 300086 (265594,331706) | 249.2 (219.7,276.9) | 635444 (555490,709238) | 302.9 (266,338.8) | 21.5 (12.6,31.2) |
| Greece | 59717 (54719,64585) | 424.9 (388.7,459.4) | 112985 (100431,122488) | 435.5 (396.1,475.3) | 2.5 (-2.4,7.8) |
| Iceland | 348 (298,399) | 120.5 (102.8,138.3) | 884 (755,1011) | 149.8 (127.2,172.4) | 24.3 (14.6,35) |
| Ireland | 9959 (8464,11701) | 256.4 (219,299.9) | 18990 (16118,22176) | 246.7 (207,289.4) | -3.8 (-11.2,4) |
| Israel | 22516 (20420,24257) | 480.7 (435.3,518.1) | 52713 (46207,58485) | 417.3 (366.3,463.5) | -13.2 (-18.5,-8.2) |
| Italy | 198890 (174312,221494) | 245.8 (215.5,275.2) | 328820 (282849,371176) | 217 (185,247.9) | -11.7 (-17,-5) |
| Luxembourg | 1331 (1185,1491) | 264.3 (234.3,295.6) | 3013 (2613,3410) | 276.4 (240,314) | 4.6 (-4.4,15.9) |
| Malta | 1203 (1076,1338) | 303 (271.3,336) | 2837 (2467,3202) | 305.7 (267.1,347.2) | 0.9 (-7.2,9.4) |
| Monaco | 121 (100,145) | 182.2 (150.3,216.1) | 228 (194,266) | 233.4 (198.8,273.1) | 28.1 (8.3,52) |
| Netherlands | 37878 (32720,42847) | 202.1 (174.6,228.7) | 82167 (72158,93046) | 238.5 (208.3,270.3) | 18 (10.4,26.3) |
| Norway | 9013 (7541,10541) | 137.2 (113.6,160.3) | 17052 (14870,19536) | 168.6 (144,194.1) | 22.9 (17,30.3) |
| Portugal | 40610 (36847,44376) | 330.5 (300.6,360.9) | 76569 (67526,85773) | 296.5 (262.7,332.8) | -10.3 (-17.5,-2.7) |
| San Marino | 56 (47,66) | 167.1 (138.9,195.7) | 113 (89,140) | 145.8 (113.8,182.3) | -12.7 (-26.7,1.8) |
| Spain | 156194 (141120,171158) | 309.6 (280.3,339) | 243084 (212322,273844) | 225.4 (196.3,255.5) | -27.2 (-32.5,-21.9) |
| Sweden | 19881 (16791,23071) | 141.2 (119.7,164.3) | 44455 (38202,50852) | 196.3 (169.2,224.9) | 39 (26.9,52.4) |
| Switzerland | 21014 (18209,23719) | 208.2 (180.6,235.2) | 44996 (38424,51285) | 228.6 (194.4,262.7) | 9.8 (1.7,20.1) |
| United Kingdom | 155905 (129827,180904) | 192.4 (160.6,223) | 258501 (216482,299277) | 213.4 (177.7,247.3) | 10.9 (6.6,16.1) |
| **Western Sub-Saharan Africa** | 1112629 (952298,1275995) | 928.7 (800.3,1067.1) | 2437269 (1992652,2893649) | 930.7 (788.4,1081.1) | 0.2 (-13.2,13.9) |
| Benin | 29653 (25048,34516) | 999.3 (868.4,1155.7) | 71721 (57427,87409) | 990.1 (808.6,1210.3) | -0.9 (-20,22) |
| Burkina Faso | 60272 (50415,71118) | 1014.2 (852.5,1190.6) | 140312 (112697,173625) | 1094.7 (897.5,1322.3) | 7.9 (-15.9,35) |
| Cabo Verde | 1204 (1015,1391) | 462.9 (389,534.5) | 3178 (2305,3934) | 677.4 (486.9,835) | 46.3 (5,85.6) |
| Cameroon | 80907 (63460,102530) | 1348.1 (1083,1721.9) | 224422 (163790,306794) | 1320 (973,1795.1) | -2.1 (-22.9,28) |
| Chad | 28291 (22969,36811) | 736.8 (598.1,968.7) | 73527 (56687,98420) | 840.5 (646.7,1175.4) | 14.1 (-9.7,44) |
| Gambia | 4626 (3620,5727) | 892.6 (714,1097.6) | 13852 (10535,17707) | 1074.8 (822.4,1368.1) | 20.4 (-11.9,62) |
| Ghana | 67702 (52675,92629) | 772.8 (608.9,1076.7) | 237458 (184981,297856) | 1145 (904.6,1420.1) | 48.2 (-1.5,98.7) |
| Guinea | 39480 (32723,48547) | 900.2 (741.3,1118.7) | 67059 (52117,90058) | 897 (697.8,1221.3) | -0.4 (-23.3,28.6) |
| Guinea-Bissau | 8272 (6561,10146) | 1414.4 (1129.6,1709.7) | 12715 (9952,16050) | 1220.6 (970.4,1525.5) | -13.7 (-33.3,14.3) |
| Liberia | 20964 (16740,25988) | 1241 (1006.8,1562.2) | 35243 (26717,46785) | 1197 (916.2,1548.1) | -3.5 (-29.9,30.8) |
| Mali | 56774 (47423,68232) | 1040.3 (873.6,1283.9) | 114837 (94129,145340) | 950.5 (780.4,1210.6) | -8.6 (-26,14.4) |
| Mauritania | 13123 (10398,16336) | 1080.7 (859.4,1353.4) | 23187 (16553,31976) | 937.8 (665.7,1292.5) | -13.2 (-32.7,12.4) |
| Niger | 41923 (33643,50607) | 842.5 (698.1,1076.1) | 80122 (59677,120176) | 687.8 (523.4,1007.5) | -18.4 (-37.6,7) |
| Nigeria | 506251 (409099,599064) | 881 (719.4,1053.3) | 1007285 (771422,1268598) | 824.4 (662.5,1015.2) | -6.4 (-24.9,14.4) |
| Sao Tome and Principe | 1102 (882,1290) | 1349 (1113.6,1530.2) | 2097 (1609,2634) | 1636.6 (1240,1979.7) | 21.3 (-9.9,54.3) |
| Senegal | 49306 (41083,60783) | 1065.1 (898.2,1309.7) | 95390 (73587,131487) | 1027.6 (799,1391.7) | -3.5 (-26.3,30.5) |
| Sierra Leone | 24287 (18928,30087) | 832.1 (664.2,1021.9) | 39388 (29974,52547) | 770.1 (604.3,1010.8) | -7.5 (-28,19.1) |
| Togo | 16010 (13190,19324) | 839.2 (706.9,1015.6) | 42127 (31777,54924) | 888.2 (691.8,1154.8) | 5.8 (-17.7,36.6) |
| CÃ ́te d'Ivoire | 62444 (50103,77072) | 1012.2 (827.3,1226.8) | 153321 (116483,194558) | 1019.7 (809.3,1277.2) | 0.7 (-18.7,29.2) |
